# Characterization, comparative phylogenetic, and gene transfer analyses of organelle genomes of *Rhododendron* × *pulchrum*

**DOI:** 10.3389/fpls.2022.969765

**Published:** 2022-09-21

**Authors:** Jianshuang Shen, Xueqin Li, Mingzhi Li, Hefeng Cheng, Xiaoling Huang, Songheng Jin

**Affiliations:** ^1^Jiyang College, Zhejiang A&F University, Zhuji, China; ^2^Department of Life Science and Health, Huzhou College, Huzhou, Zhejiang, China; ^3^Bio and Data Biotechnology Co., Ltd., Guangzhou, China

**Keywords:** *Rhododendron*, mitochondrial genome, chloroplast genome, gene transfer, phylogenetic

## Abstract

Rhododendron × *pulchrum*, an important horticultural species, is widely distributed in Europe, Asia, and North America. To analyze the phylogenetic and organelle genome information of *R.* × *pulchrum* and its related species, the organelle genome of *R.* × *pulchrum* was sequenced and assembled. The complete mitochondrial genome showed lineage DNA molecules, which were 816,410 bp long and contained 64 genes, namely 24 transfer RNA (tRNA) genes, 3 ribosomal RNA (rRNA) genes, and 37 protein-coding genes. The chloroplast genome of *R.* × *pulchrum* was reassembled and re-annotated; the results were different from those of previous studies. There were 42 and 46 simple sequence repeats (SSR) identified from the mitochondrial and chloroplast genomes of *R.* × *pulchrum*, respectively. Five genes (*nad1*, *nad2*, *nad4*, *nad7*, and *rps3*) were potentially useful molecular markers. The *R.* × *pulchrum* mitochondrial genome collinear alignment among five species of the Ericaceae showed that the mitochondrial genomes of these related species have a high degree of homology with *R.* × *pulchrum* in this gene region, and the most conservative genes were *trnC-GCA*, *trnD-GUC*, *trnM-CAU*, *trnN-GUU*, *trnY-GUA*, *atp4*, *nad4*, *nad2*, *nad5*, *ccmC*, and *rrn26*. The phylogenetic trees of mitochondrial genome showed that *R*. *simsii* was a sister to *R.* × *pulchrum*. The results verified that there was gene rearrangement between *R.* × *pulchrum* and *R*. *simsii* mitochondrial genomes. The codon usage bias of 10 Ericaceae mitochondrial genes and 7 *Rhododendron* chloroplast genes were influenced by mutation, while other genes codon usages had undergone selection. The study identified 13 homologous fragments containing gene sequences between the chloroplast and mitochondrial genomes of *R.* × *pulchrum*. Overall, our results illustrate the organelle genome information could explain the phylogenetics of plants and could be used to develop molecular markers and genetic evolution. Our study will facilitate the study of population genetics and evolution in *Rhododendron* and other genera in Ericaceae.

## Introduction

Plants contain two organelles: the plastid and mitochondrion. These organelles retain their own genomes, which originated independently from nuclear genomes (Gray, 2012). The plastid genomes of higher plants are conserved in size, gene content, gene structure, and gene order (Sugiura, 1995; Wicke et al., 2011). Most plastomes are a circular molecule of double-stranded DNA containing four typical regions, ranging from 72 to 217 kb in size and containing ∼130 genes (Jansen et al., 2011). Plastid genomes with conserved structures and high substitution rates are used to study phylogeny, biology, and photosynthetic gene degradation in plants (Wu and Ge, 2012; Thomas et al., 2017; Shen et al., 2020).

Compared with plastid genomes, plant mitochondrial genomes have a broad distribution in size, from 66 to 11,000 kb, multipartite genome variations, arrangements among species, gene sequence transfer or loss, and other unique features (Knoop, 2004; Parkinson et al., 2005; Sloan et al., 2012; Wu et al., 2022). The mitochondrial genome size, chromosome number, and copy number variations have been explored in plants (Alverson et al., 2011). For example, the *Arabidopsis thaliana* mitochondrial genome has a typical circular structure (Sloan et al., 2018), while in *Silene conica* and *Chenopodium album*, the mitochondrial genomes possess linear or multichromosomal architectures (Backert and Borner, 2000; Sloan et al., 2012; Sanchez-Puerta et al., 2017). The mitochondrial genome could be used to develop molecular markers and analyze mitochondrial genome expansion mechanisms (Zubaer et al., 2018). Comparing mitochondrial genomes with related species could provide a new way to explain evolutionary mechanisms and mitochondrial genome rearrangements and to identify species taxa (Li et al., 2018; Zubaer et al., 2018).

The whole organelle genomes of plants have been sequenced on an Illumina Hiseq Platform (Shen et al., 2020; Xu et al., 2021). However, Illumina read lengths often do not span longer repeats; these regions are incompletely assembled and thus influence the accuracy of the length and content of the genome. With the development of this technique, third-generation sequencing (TGS) methods, such as Oxford Nanopore and PacBio sequencing with long-read length, could improve the coverage and assembly accuracy of previously unassembled genomic regions and is a useful tool to understand plant organelle genome information (Shearman et al., 2016). Thousands of plant plastid genomes and hundreds of complete land plant mitochondrial genomes are currently available, with most of these coming from crop species (Wu et al., 2022). Ericales comprises about 25 families (Anderberg et al., 2002), and only 10 plant species from 5 families have mitochondrial genome data published in the NCBI GenBank database. Few mitochondrial genomes of *Rhododendron* have been published, only report simple gene annotation information (Xu et al., 2021). Genes from mitochondrial genomes could provide a new explanation for the phylogenetic relationships among species in Ericales. Phylogenetic interrelationships in Ericales need to be further investigated using molecular data accumulated from multiple genes (Anderberg et al., 2002). The complete plastome sequences of 11 species of the genus *Rhododendron* belonging to Ericaceae are currently available in the NCBI GenBank database; these include five species (*Rhododendron delavayi* var. Delavayi, *R*. *griersonianum*, *R*. *henanense* subsp. lingbaoense, *R*. *platypodum*, and *R*. *delavayi*.) belonging to the Subgen. Hymenanthes, one species (*R*. *molle*) belonging to Subgen. Pentanthera, and five species (*R*. *kawakamii*, *R*. *datiandingense*, *R*. *micranthum*, *R*. *concinnum*, and *R*. *simsii*) belonging to Subgen. Tsutsusi. Most of these *Rhododendron* chloroplast genomes were assembled by second-generation sequencing, analyzing the genome structural and phylogeny (Thomas et al., 2017; Li et al., 2018; Zubaer et al., 2018). The results of these studies were quite different. Such as whether exit IRs region, and the results are surprisingly differences among *Rhodordendron* species. Compared with plastome sequences, *Rhodordendron* species mitochondrial genome-related studies on genome variations, arrangements, chloroplast-to-mitochondrial gene transfer have been poorly understood, depending on limited genomic data. With the development of new techniques, increasing organelle genome data have been published and analyzed, and chloroplast-to-mitochondrial gene transfer has been considered a characteristic feature of long-term evolution (Gui et al., 2016; Nguyen et al., 2020). Previous studies mainly focused on the gene transfer of nuclear DNA from the organelle in angiosperms (Smith, 2011; Park et al., 2014). Thus, the mitochondrial and plastid genome information of *Rhodordendron* species need to be further studies.

*Rhododendron* × *pulchrum* Sweet (Hirado azalea, *R.* × *pulchrum*), an important horticultural species, is widely distributed in the temperate regions of Europe, Asia, and North America (Galle, 1985). *R.* × *pulchrum* is considered as horticultural cultivar of “Omurasaki” and Hirado azalea cultivars. Recent study revealed the genetic relationship of *R.* × *pulchrum* and its related cultivars with putative ancestral species (including *R. ripense* Makino, *R. macrosepalum*, *R. scabrum*, *R.* × *pulchrum* “Ômurasaki,” and *R.* × *mucronatum* “Shiro-ryûkyû) using F3”5’H gene sequences and AFLP technique (Scariot et al., 2007; Meanchaipiboon et al., 2021). In addition, the chloroplast (cpDNA) origin of “Omurasaki” and Hirado azalea cultivar groups were reported in Kobayashi et al. (2021). “Omurasaki” and the most of Hirado azalea cultivar owned cpDNA of *R. ripense*, Japanese wild azalea (Shirasawa et al., 2021). So far, there has been no report on the mitochondrial genome of *R.* × *pulchrum*. Further studies on the mitochondrial and plastid genome information of *Rhodordendron* species are needed, which will facilitate the study of population genetics and evolution in *Rhododendron* and help to understand the evolutionary mechanisms and identifying species taxa in Ericaceae. The previously published plastid genome of *R.* × *pulchrum* was sequenced with the Illumina Hiseq Platform (Shen et al., 2019, 2020). Herein, we used TGS methods combined with second-generation sequencing technology to detect the complete mitochondrial and plastid genomes of *R.* × *pulchrum*. We assembled and annotated the complete mitochondrial and plastid genomes of *R.* × *pulchrum* and analyzed the genome content, organization, and phylogenetic analysis. We performed a comparative mitogenomic analysis of the *Rhododendron* species to identify regions of variation, conservation, and rearrangement across the genomes. We also analyzed gene transfer between the mitochondrial and plastid genomes of *R.* × *pulchrum*. The mitochondrial and plastid genome information could explain the phylogenetic and evolutionary relationships of plants and could be used to develop molecular markers and genetic engineering.

## Materials and methods

### DNA extraction and sequencing

The young green leaves of cutting clones of *Rhododendron* × *pulchrum* Sweet cultivars with purple large flowered type (Supplementary Figure 1) were collected from the nursery of Zhejiang A&F University (stored in the Institute of Botany, Chinese Academy of Sciences Mem, and the specimen accession number is PE00820836) and stored immediately at −80°C. Total genomic DNA was extracted from the young leaves using the modified Cetyltrimethylammonium Bromide (CTAB) method (Doyle, 1987). High-quality DNA was used for subsequent library preparation and sequencing using PromethION and BGISEQ-500 platforms (Bio & Data Biotechnologies Co., Ltd., Guangzhou, China). To obtain long non-fragmented sequence reads, ∼15 μg of genomic DNA was sheared and size-selected (30–80 kb) with a BluePippin (Sage Science, Beverly, MA, USA). The selected fragments were processed using the Ligation Sequencing 1D Kit (Oxford Nanopore, Oxford, UK) according to the manufacturer’s instructions and sequenced using the PromethION DNA sequencer (Oxford Nanopore, Oxford, UK) for 48 – 72 h.

Following DNA extraction, we fragmented 1 μg of purified DNA and used it to set up 300 bp short-insert libraries. These qualified libraries were sequenced with PE150 bp on a BGISEQ-500 sequencer according to the manufacturer’s instructions. Sequencing was performed using SPAdes v-3.13.0 software (Bankevich et al., 2012), and TGS data were assembled individually using Canu v-1.5 software (Koren et al., 2017). The assembled contigs were aligned with all the manufacturer’s instructions detailed in the literature (Huang et al., 2017).

### Preprocessing of sequenced reads

For the long reads, adapter trimming was performed using Porechop v0.2.4^1^ and removing reads with quality score < 7 was performed using Guppy. For the short reads, raw reads were preprocessed by Fastp v.0.20.1 with default parameters (Chen et al., 2018) in order to trim adaptors and remove the low-quality reads (Phred quality scores < 20).

### Assembly of the mitochondrial genome

Mixed *de novo* assembly for third- and second-generation sequencing was performed using SPAdes v-3.13.0 software (parameters −k 21,33,55,77,89 –careful, orther default) (Bankevich et al., 2012), and third-generation (long) sequencing data were assembled individually using Canu v-1.5 software with settings of (1) genome size of 0.8 Mb and (2) corrected Error Rate = 0.03 (Koren et al., 2017). The assembled contigs were aligned with all mitochondrial sequences of the Ericales species from the NCBI, and candidate mitochondrial contigs were extracted. The candidate mitochondrial contigs were then polished using Pilon software (Walker et al., 2014), and the second-generation (short) sequencing read extensions were performed on the contigs using Geneious prime software (Kearse et al., 2012). The repeats at the ends of the selected mitochondrial contigs were identified using Geneious Prime software. Contigs were linked based on the terminal repeats, and the assembled long contigs were subjected to short read comparison and end extension until no reads could be further extended (Kearse et al., 2012). The assembly was identified by comparing the mitochondrial genome of 4 species from Ericales, namely *R*. *simsii* (NC053763), *Vaccinium macrocarpon* (NC023338), *Monotropa hypopitys* (MK990822), and *Arctostaphylos glauca* (MZ779111), as a reference.

### Assembly of the chloroplast genome

The TGS reads were aligned to all chloroplast genome data of the Ericales species from NCBI using Minimap2 software (Li, 2018), and reads with alignment lengths greater than 5,000 bp were extracted for subsequent assembly. The second-generation sequencing reads were download from Genbank and aligned with the company’s (Bio and Data Biotechnology Co., Ltd., Guangzhou, China) self-built chloroplast genome database using Bowtie2 software (Langmead and Salzberg, 2012), and the aligned reads were used for subsequent assembly. The chloroplast candidate third- and second-generation reads extracted above were used for chloroplast genome assembly using Unicycler version: v0.4.8 software with default parameters (Wick et al., 2017). The chloroplast genome of *R.* × *pulchrum* was reassembled and re-annotated in our study; all new reads were deposited to the NCBI Sequence Read Archive (SRA) under accession number MN182619.2.

Complete chloroplast genome collinear alignment compared between MN182619.1 and MN182619.2 of *Rhododendron* × *pulchrum* had been compared using LASTZ software (version 1.02.00) (Harris, 2007; Kearse et al., 2012). The breakage and inversion sites were randomly selected to design primers for Polymerase Chain Reaction (PCR) experiments to verified the accuracy of the chloroplast genome.

### Annotation of the mitochondrial and chloroplast genomes

Mitochondrial and chloroplast genome annotation was performed using GeSeq software (Tillich et al., 2017), and annotation results were manually corrected using Geneious prime (Kearse et al., 2012). The genome map was drawn using the Organellar Genome DRAW tool (OGDRAW) v.1.3.1 for further comparison of gene order and content (Greiner et al., 2019). The relative synonymous codon usage (RSCU) was calculated following Sharp and Li (1986).

### Identification of repeats

Simple sequence repeats (SSR) using MISA (MIcroSAtellite identification tool) software for SSR analysis (Thiel et al., 2003), and parameters were set as follows (unit_size, min_repeats): 1–10 2–6 3–5 4–5 5–5 6–5, interruptions (max_difference_between_2_SSRs): 100. Using a tandem repeat finder to analyze tandem repeat sequences, the parameters were set as 2, 7, 7, 80, 10, 50, 500, −f, −d, and −m (Benson, 1999).

### Phylogenetic analysis

Phylogenies were constructed by maximum likelihood (ML) using Fasttree software (Price et al., 2010). The sequences were initially aligned using MAFFTv7.313 (Katoh and Standley, 2013), and the ML tree was constructed using Fasttree 2 software under the GTR + Gamma model (Price et al., 2010).

The mitochondrial phylogenetic tree selected 8 coding gene fragments (*atp1, atp4, atp9, ccmC, matR, nad3, nad6*, and *rps12*) shared by the mitochondrial genomes of 13 related species: *R.* × *pulchrum* (OM283814), *R. simsii* (NC053763), *Vaccinium macrocarpon* (NC023338), *Arctostaphylos glauca* (MZ779111), *Monotropa hypopitys* (MK990822), *Aegiceras corniculatum* (NC056358), *Argania spinosa* (MZ151883), *Camellia sinensis* var. Assamica (MK574877), *Camellia sinensis* (NC043914), *Actinidia eriantha* (MZ959063), *A*. *chinensis* (MZ959061), and *A*. *arguta* (MH559343). *Vitis vinifera* (NC012119) was used as an outgroup sample for sequence alignment. The mitochondrial evolutionary tree of Ericales was constructed in this study. The length of the homologous part for the 8 genes in 13 related species had been shown in Supplementary Table 1.

The chloroplast evolutionary tree selected 57 coding gene fragments (*atpA, atpB, atpE, atpH, atpI, cemA, matK, ndhA, ndhB, ndhC, ndhD, ndhE, ndhF, ndhH, ndhI, ndhJ, petA, petB, petD, petG, petL, petN, psaA, psaB, psaC, psaI, psbA, psbB, psbC, psbD, psbE, psbF, psbJ, psbK, psbL, psbM, rbcL, rpl2, rpl14, rpl22, rpl23, rpl32, rpl33, rpl36, rpoA, rpoC1, rpoC2, rps2, rps3, rps4, rps7, rps8, rps11, rps12, rps14, rps15*, and *rps18*) shared by the genomes of closely related species for sequence alignment, and the involved 15 species, namely *Rhododendron delavayi* var. delavayi (NC047438), *R*. *griersonianum* (NC050162), *R*. *henanense* subsp. lingbaoense (MT239363), *R*. *platypodum* (MT985162), *R*. *delavayi* (MN711645), *R*. *molle* (MZ073672), *R*. *kawakamii* (NC058233), *R. datiandingense* (NC057644), *R*. *micranthum* (MT239365), *R*. *concinnum* (MT239366), *R. ripense* (DRR298903-DRR298907), *R. ovatum* (SRR12917131-SRR12917132), *R. latoucheae* (SRR13425299), *R.* × *pulchrum* (MN182619.2) and *R*. *simsii* (MT239364). *Actinidia deliciosa* (NC026691), and *A*. *chinensis* (NC026690) were used as outgroup samples for sequence alignment. The *Rhododendron* genus has extensive chloroplast data; thus, only the *Rhododendron* chloroplast evolutionary tree was constructed in our study. The length of the homologous part for the 57 genes 15 related species had been shown in Supplementary Table 2.

### Codon usage bias patterns analysis

Codon usage bias parameters RSCU and Effective number of codons (ENC) were calculated using CondonW 1.4.4 software (Peden and John, 2000). ENC is used to analyze the effect of gene base composition on codon usage bias (Wright, 1990). These two parameters were used to describe the patterns of codon usage bias. Five mitochondrial genomes from Ericaceae were selected to calculated parameters of codon usage bias, including *Monotropa hypopitys* (MK990822), *Arctostaphylos glauca* (MZ779111), *Vaccinium macrocarpon* (NC023338), *Rhododendron simsii* (NC053763), and *R.* × *pulchrum* (OM283814). The 15 chloroplast genomes of *Rhododendron* were selected to calculated parameters of codon usage bias, including *Rhododendron delavayi* var. delavayi (NC047438), *R*. *griersonianum* (NC050162), *R*. *henanense* subsp. lingbaoense (MT239363), *R*. *platypodum* (MT985162), *R*. *delavayi* (MN711645), *R*. *molle* (MZ073672), *R*. *kawakamii* (NC058233), *R. datiandingense* (NC057644), *R*. *micranthum* (MT239365), *R*. *concinnum* (MT239366), *R. ripense* (DRR298903-DRR298907), *R. ovatum* (SRR12917131-SRR12917132), *R. latoucheae* (SRR13425299), and *R*. *simsii* (MT239364), and *R.* × *pulchrum* (MN182619.2).

### Genomic comparison of related species and horizontal gene transfer between chloroplast and mitochondrial genomes

The mitochondrial genome of *R.* × *pulchrum* was used as a reference, and it was compared with *R*. *simsii* (NC_053763) to determine the mitochondrial rearrangement. Mauve software was used to analyze mitochondrial rearrangement and draw structural variation (Darling et al., 2004).

The mitochondrial genomes of *R.* × *pulchrum* (OM283814) and *R*. *simsii* (NC_053763) were analyzed for collinearity using Dottup software (Rice et al., 2000). LASTZ version 1.02.00 was used to perform a whole-gene collinear alignment to detect the collinear segment (Harris, 2007; Kearse et al., 2012). The parameters were set as follows: step = 20 and seed pattern = 12 of 19. The results were displayed using the *R.* × *pulchrum* mitochondrial genome as a reference and compared with four other genomes belonging to the Ericaceae family (*R*. *simsii*, *V*. *macrocarpon*, *A*. *glauca*, and *M*. *hypopitys*). Horizontal gene transfer (HGT) between the chloroplast and mitochondrial genomes was determined using LASTZ version 1.02.00 (Harris, 2007).

## Results

### Mitochondrial genome organization and features

The mitochondrial genome assembly had an average read coverage higher than 325 × in this study. The genebank accession number of the *R.* × *pulchrum* mitochondrial genome is OM283814. Whole mitochondrial genome (mitogenome) sequencing produced 36,533,204 clean reads (SRA accession number SRR17758703) from the second-generation sequencing platform and 5,364,975 pass reads (SRA accession number SRR17758704) from the TGS platform. The sequencing and assembly statistics are summarized in Supplementary Table 3.

**FIGURE 1:**
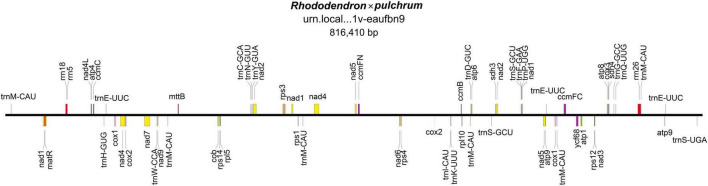
Gene map of the *Rhododendron* × *pulchrum* mitochondrial genome.

The mitochondrial genome was composed of lineage DNA molecules and assembled at 816,410 bp, with GC contents of 45.7% for *R.* × *pulchrum* (Figure 1 and Supplementary Figure 4). The complete mitochondrial genome of *R.* × *pulchrum* contained 64 annotated genes, namely, 24 transfer RNA (tRNA) genes, 3 ribosomal RNA (rRNA) genes, and 37 protein-coding genes (mRNA) (Table 1). These gene positions and arrangements are depicted in Figure 1.

**TABLE 1:** Annotated genes in the *Rhododendron* × *pulchrum* mitochondrial genome.

Group of genes	Name of gene
Ribosomal RNA	*rrn5*, *rrn18*, *rrn26*
Transfer RNA	*trnC-GCA*, *trnD-GUC*, *trnE-UUC*^2^, *trnF-GAA*, *trnG-GCC*, *trnH-GUG*, *trnI-CAU*, *trnK-UUU*, *trnM-CAU*^3^, *trnN-GUU*, *trnP-UGG*, *trnQ-UUG*, *trnS-UGA*, *trnS-GCU*^1^, *trnW-CCA*, *trnY-GUA*,
Small subunit of ribosome	*rps1*, *rps3*^4^, *rps4*, *rpl5*, *rpl10*, *rps12*, *rps14*^4^,
Subunits of ATP synthase	*atp1*, *atp4*, *atp6*, *atp8*, *atp9*^1^
NADH dehydrogenase (complex I)	*nad1*^6^, *nad2*^6^, *nad3*, *nad4*^1^,^5^, *nad4L*, *nad5*^6^, *nad6*, *nad7*^6^, *nad9*
Cytochrome c oxidase (complex IV)	*cox1*^1^, *cox2*^4^, *cox3*,
Ubichinol cytochrome c reductase (complex III)	*cob*
Maturases	*matR*
Succinate dehydrogenase (complex II)	*sdh3*, *sdh4*
Other genes	*ccmB*, *ccmC*, *ccmFC*^4^, *ccmFN*, *mttB*, *ycf68*

^1^Two gene copies; ^2^three gene copies; ^3^six gene copies; ^4^gene containing a single intron; ^5^gene containing two introns; ^6^gene containing two introns.

In total, 9 intron-containing genes (*rps14*, *Cox2*, *ccmFC*, *rps3*, *nad1*, *nad 2*, *nad4*, *nad5*, and *nad7*) were annotated (Table 1), among which *nad4* had protein-coding genes with two introns, *nad1*, *nad 2*, *nad5*, and *nad7* protein-coding genes had four introns, and the others had one intron.

We then estimated the codon usage frequency based on the protein-coding and tRNA genes. As shown in Supplementary Table 5 and Supplementary Figure 2, UUC, AUU, and UUU were the most frequent codons, contributing to the high AU content of the *R.* × *pulchrum* mitochondrial genome (54.3%). The mitochondrial genome was composed of 11,196 codons (65 different types) encoding 20 amino acids, among which leucine (Leu) was the most frequently used amino acid (11.59%, 1,298), and cysteine (Cys) was the least abundant (1.46%, 163) (Supplementary Table 5). The results suggest that the *R.* × *pulchrum* mitochondrial genome prefers synonymous codons ending with A or T with a RSCU > 1, excepting UUG ending with G.

Forty-two SSRs were identified from the *R.* × *pulchrum* mitochondrial genome, with 36 mononucleotides (63.35%), 4 dinucleotides (2.71%), and 2 trinucleotides (26.24%) (Supplementary Table 6). Two SSRs contained guanine (G) or cytosine (C), whereas the remaining 34 SSRs had either polyadenine (poly A) or polythymine (poly T). Six SSR markers were located in gene coding regions, including AG (6) and CT (6) located in the *nad4* gene, two A (10) located in the *nad1* and *nad2* genes, and two A (12) located in the *nad7* and *rps3* genes. A total of 28 tandem repeats (TR) were identified in the *R.* × *pulchrum* mitochondrial genome (Supplementary Table 7). Among these, only one TR (742,682–742,724 bp) was located in the *rrn26* gene; the others were all located in intergenic spaces.

### Mitochondrial genome comparison

**FIGURE 2:**
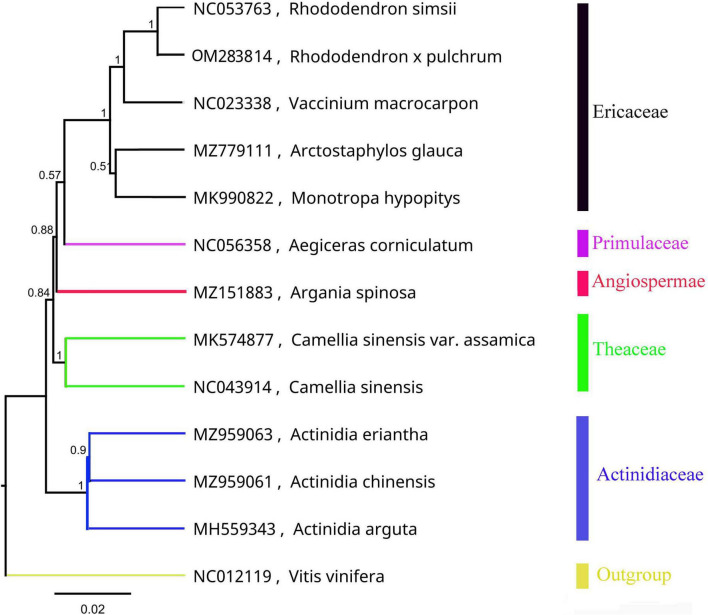
Phylogenetic tree of Ericales species based on mitochondrial genomes. *Vitis vinifera* (NC012119) was used as the outgroup.

The mitochondrial phylogenetic tree from Ericales showed that the 8 species were clustered into four groups, which is consistent with their taxonomic information. Five species in the Ericaceae family were clustered into one clade: *R*. *simsii*, *V*. *macrocarpon*, *A*. *glauca*, *M*. *hypopitys*, and *R.* × *pulchrum*. Furthermore, *R*. *simsii* was found to be a sister to *R.* × *pulchrum* (Figure 2).

**FIGURE 3:**
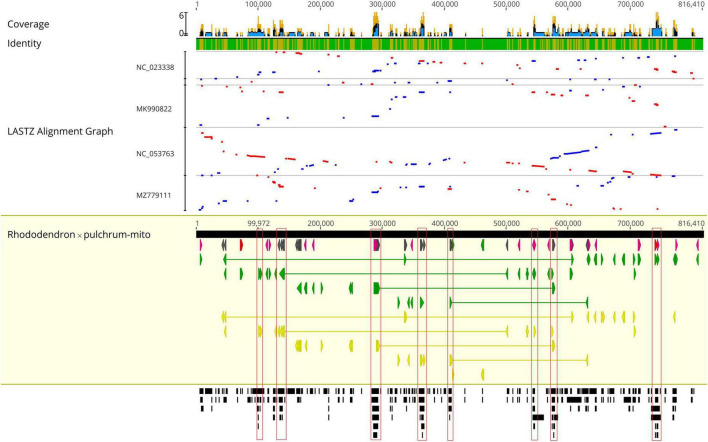
Complete mitochondrial genome collinear alignment compared between five species from the Ericaceae family. (*Rhododendron* × *pulchrum* as the reference; blue bars represent homologous high-scoring segment pairs in a codirectional orientation, whereas red bars represent reversed pairs. Yellow arrows represent coding sequences, red arrows represent ribosomal RNA genes, purple arrows represent transfer RNA genes, green arrows represent protein-coding genes, and gray arrows represent exonic regions. Black squares represent homology; the larger the number, the larger the area of the black squares, and the more closely related species have homologous fragments. Red wireframes are the most conservative regions. These regions, marked from left to right in the figure, are distributed from left to right and contain the following genes: *atp4* and *ccmC*; *nad4*; *trnC-GCA*, *trnN-GUU*, *trnY-GUA*, and *nad2*; *nad4*; *nad5*; *trnM-CAU* and *trnD-GUC*; *nad2*; *rrn26*, and *trnM-CAU*).

The *R.* × *pulchrum* mitochondrial genome was used as a reference, and a whole-genome collinear alignment was compared among five species from the Ericaceae family, as shown in Figure 3. The mitochondrial genome of these related species had a high degree of homology with *R.* × *pulchrum* in the gene region, in which the most conservative genes were *atp4*, *ccmC*, *nad4*, *trnC-GCA*, *trnN-GUU*, *trnY-GUA*, *nad2*, *nad5*, *trnM-CAU*, *trnD-GUC*, and *rrn26*. *R.* × *pulchrum* and *R*. *simsii* mitochondrial genomes had more homologous coverage than the other three species of Ericaceae. Furthermore, the mitochondrial genome organization between *R.* × *pulchrum* and *R*. *simsii* mitochondrial genomes showed that there was still complex variation (Supplementary Figure 3).

**FIGURE 4:**
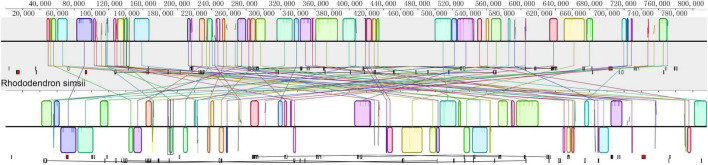
Mauve visualization of mitochondrial genome-wide comparison between *Rhododendron* × *pulchrum* and *R*. *simsii*. [The progressive mauve alignment (in the Mauve program) shows the homologous blocks shared among the mitochondrial genomes, and it also connected these blocks with lines, indicating the corresponding position among the homologous blocks to visualize the gene arrangement].

The mitochondrial genome of *R.* × *pulchrum* was used as a reference, and it was compared with *R*. *simsii* (NC_053763) to analyze mitochondrial genome rearrangement and collinearity using the Mauve and Lastz programs. The mitochondrial genomes of *R.* × *pulchrum* and *R*. *simsii* were divided into 63 locally collinear blocks (LCB). These LCBs differed substantially in the size and relative position of the two *Rhododendron* species studied (Figure 4). Moreover, most LCBs contained gene sequences, with 42 genes involved in total (Supplementary Table 8). The rearrangement analysis indicated that many genes had rearrangements between *R.* × *pulchrum* and *R*. *simsii* mitochondrial genomes (Supplementary Table 8).

### Chloroplast genome organization and features

**FIGURE 5:**
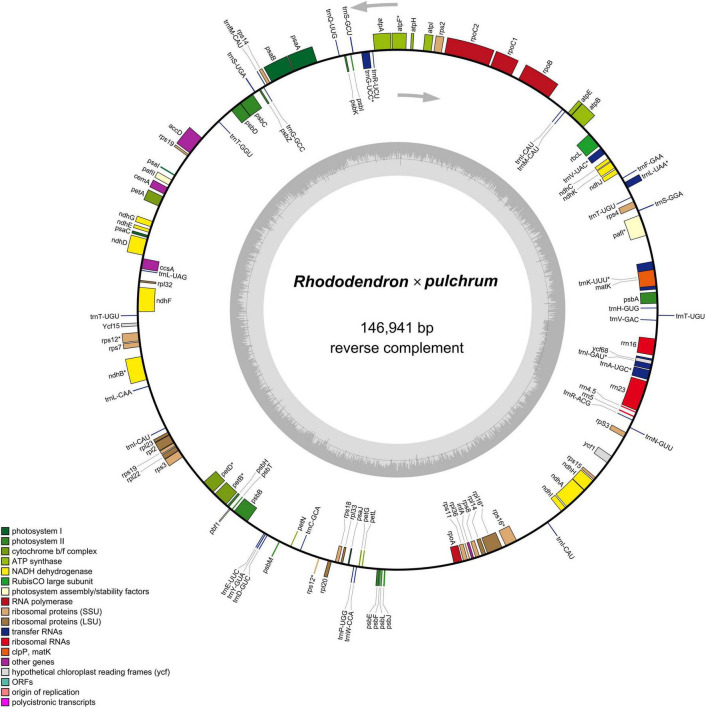
Gene map of the *Rhododendron* × *pulchrum* chloroplast genome. (Genes residing in the inside and outside of the outer circle are in the forward and reverse directions, respectively. The dark and light gray bars in the inner circle denote the G + C and A + T contents, respectively).

The results showed that the complete *R.* × *pulchrum* chloroplast genome was 146,941 bp in length (Figure 5) and did not take the form of a typical quadripartite structure, due to the lack of inverted repeats (IR). The GC content of the chloroplast genome of *R.* × *pulchrum* was 35.8%. The complete chloroplast genome of *R.* × *pulchrum* contained 119 genes that were annotated, namely 34 tRNA genes, 4 rRNA genes, and 81 protein-coding genes (mRNA) (Table 2 and Figure 5). In total, 7 protein-coding genes (*ndhB3*, *petB*, *petD*, *atpF*, *rpl16*, *rps12*, and *rps16*) and 6 tRNA (*trnA-UGC*, *trnK-UUU*, *trnI-GAU*, *trnG-UCC*, *trnL-UAA*, and *trnV-UAC*), which were intron-containing genes, were annotated (Table 1). Among these, only *pafI4* protein-coding genes contained two introns, and the others had one intron.

**TABLE 2:** Annotated genes of the *Rhododendron* × *pulchrum* chloroplast genome.

Function	Genes
RNAs, transfer	*trnA-UGC*^3^, *trnC-GCA*, *trnD-GUC*, *trnE-UUC*, *trnF-GAA*, *trnfM-CAU*, *trnG-GCC*, *trnG-UCC*^3^, *trnH-GUG*, *trnI-CAU*^2^, *trnI-GAU*^3^, *trnK-UUU*^3^, *trnL-CAA*, *trnL-UAA*^3^, *trnL-UAG*, *trnM-CAU*, *trnN-GUU*, *trnP-UGG*, *trnQ-UUG*, *trnR-ACG*, *trnR-UCU*, *trnS-GCU*, *trnS-GGA*, *trnS-UGA*, *trnT-GGU*, *trnT-UGU*^2^, *trnV-GAC*, *trnV-UAC*^3^, *trnW-CCA*, *trnY-GUA*
RNAs, ribosomal	*rrn4*.*5*, *rrn5*, *rrn16*, *rrn23*
Transcription and splicing	*rpoA*, *rpoB*, *rpoC1*, *rpoC2*
Small subunit	*rps2*, *rps3*^1^, *rps4*, *rps7*, *rps8*, *rps11*, *rps12*^3^, *rps14*, *rps15*, *rps16*^3^, *rps18*, *rps19*^1^
Large subunit	*rpl2*, *rpl14*, *rpl16*^3^, *rpl20*, *rpl22*, *rpl23*, *rpl32*, *rpl33*, *rpl36*
ATP synthase	*atpA*, *atpB*, *atpE*, *atpF*^3^, *atpH*, *atpI*
Photosystem I	*psaA*, *psaB*, *psaC*, *psaI*, *psaJ*, *ycf1*, *ycf15*, *ycf68*, *pafI*^4^, *pafII*, *pbf1*
Photosystem II	*psbA*, *psbB*, *psbC*, *psbD*, *psbE*, *psbF*, *psbH*, *psbI*, *psbJ*, *psbK*, *psbL*, *psbM*, *psbT*, *psbZ*
Calvin cycle	*rbcL*
Cytochrome complex	*petA*, *petB*^3^, *petD*^3^, *petG*, *petL*, *petN*
NADH dehydrogenase	*ndhA*, *ndhB*^3^, *ndhC*, *ndhD*, *ndhE*, *ndhF*, *ndhG*, *ndhH*, *ndhI*, *ndhJ*, *ndhK*
Translational initiation factor	*infA*
Maturase	*matK*
Envelope membrane protein	*cemA*
Subunit of acetyl-CoA	*accD*
C-type cytochrome synthesis gene	*ccsA*

^1^Genes with two copies; ^2^Genes with three copies; ^3^Genes with one intron; ^4^Genes with two introns.

The codon usage frequency based on the protein-coding and tRNA genes is shown in Supplementary Table 9 and Supplementary Figure 4. The chloroplast genome was composed of 19,278 codons (65 different types) encoding 20 amino acids, among which leucine (Leu) was the most frequently used amino acid (10.68%, 2,058) and cysteine (Cys) was the least abundant (1.12%, 215) (Supplementary Table 8). The results suggest that the *R.* × *pulchrum* chloroplast genome prefers synonymous codons ending with A or U with a RSCU > 1 (Supplementary Table 9 and Supplementary Figure 4).

Forty-six SSRs were identified from the *R.* × *pulchrum* chloroplast genome, including 41 mononucleotides and 5 dinucleotides (Supplementary Table 10). All SSRs contained only polyadenine (poly A) or polythymine (poly T), without guanine (G) or cytosine (C).

### Chloroplast genome comparison

**FIGURE 6:**
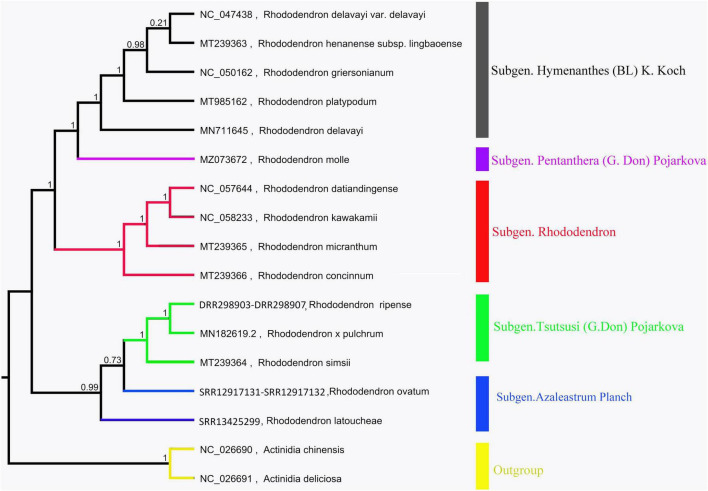
Phylogenetic tree of *Rhododendron* species based on chloroplast genomes. *Actinidia deliciosa* and *Actinidia chinensis* were used as the outgroups.

The chloroplast genome phylogenetic tree of 15 species from *Rhododendron* showed that these species were clustered into three groups, which is consistent with their taxonomic information. Five species in Subgen. Hymenanthes were clustered into one clade: *R*. *delavayi* var. Delavayi, *R*. *griersonianum*, *R*. *henanense* subsp. lingbaoense, *R*. *platypodum*, and *R*. *delavayi*. *R*. *molle* belonging to Subgen. Pentanthera was clustered into one clade. The other 6 species belonging to Subgen. Tsutsusi were clustered into one clade. Among these 6 species, *R*. *kawakamii* and *R*. *datiandingense* belonged to Sect. Vireya, *R*. *micranthum* and *R*. *concinnum* belonged to Sect. Rhododendron, and *R.* × *pulchrum* and *R*. *simsii* belonged to Sect. Tsutsusi. Furthermore, *R. ripense* was found to be a sister to *R.* × *pulchrum* (Figure 6).

### Codon usage bias patterns and evolution

Codon usage frequency of five mitochondrial genomes from Ericaceae had been shown in Supplementary Table 11, there have 30∼32 codons with a RSCU > 1 among these five species, these codons ending with A or T, excepting UUG (Leu) ending with G. Codons GCU (Ala), CAU (His), and UAA (Ter) were the most frequent codons (RSCU > 1.5), CAS (His) was the low frequent codons (RSCU < 0.5) among these five species. Codon usage frequency of 15 chloroplast genomes of *Rhododendron* had been shown in Supplementary Table 12, there have 30∼31 codons with a RSCU > 1 among these 15 species, these codons ending with A or T, excepting UUG (Leu) ending with G. Further, 14∼19 codons were the most frequent codons (RSCU > 1.5), 16∼20 codons were the low frequent codons (RSCU < 0.5) among these 15 species.

**FIGURE 7:**
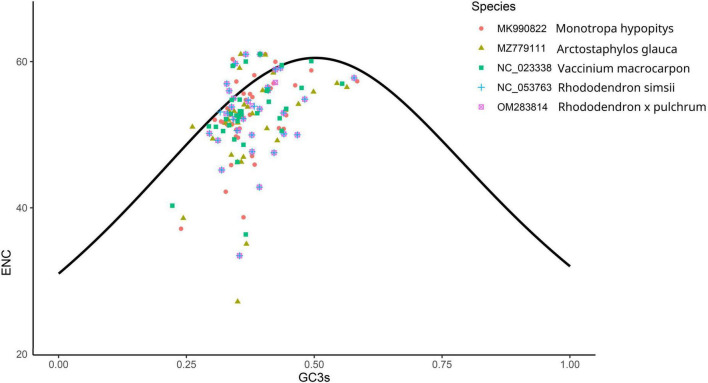
ENC Plot analysis of genes in 5 Ericales species mitochondrial genomes. (Y-axis: Effective number of codons value of gene; X-axis: GC3 content of gene; Standard curve line is calculated as follows: ENC = 2 + GC3 + 29/(GC3^2^ + (1-GC3)^2^).

**FIGURE 8:**
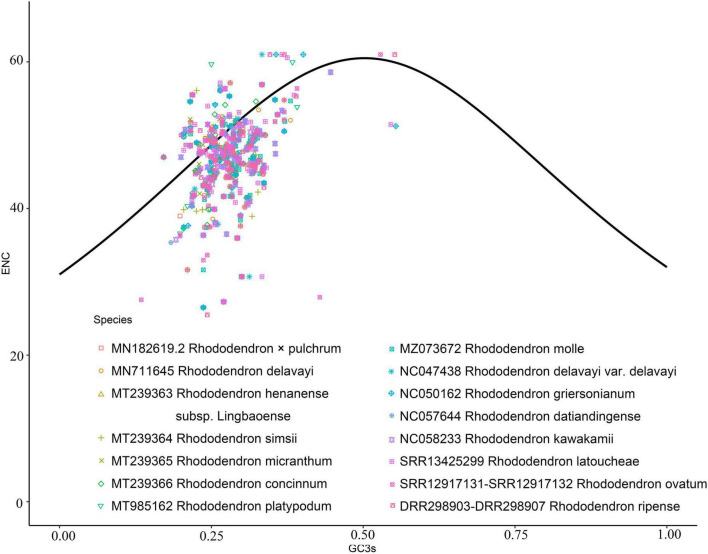
ENC Plot analysis of genes in 12 *Rhododendron* species chloroplast genomes. (Y-axis: Effective number of codons value of gene; X-axis: GC3 content of gene; Standard curve line is calculated as follows: ENC = 2 + GC3 + 29/(GC3^2^ + (1-GC3)^2^).

The number of genes of ENC value below 35 in *M. hypopitys, V. macrocarpon, A. glauca, R.* × *pulchrum*, and *R. simsii* mitochondrial genomes are 0, 0, 1 (*ccmC*), 1 (*atp9*), and 1 (*atp9*) (Supplementary Table 13). NEC plot analysis had shown that there have 10 genes on or above standard curve line in 5 Ericaceae species mitochondrial genomes, including *atp4, atp6, ccmFc, nad1, nad9, rpl10, rps3, rps10, rps12, rps19*, the other genes below standard curve line (Figure 7 and Table 3). Twenty of these genes in the five species are all below standard curve line (Figure 7 and Table 3). There had found three genes with ENC value below 35 in 15 *Rhododendron* species chloroplast genomes, including *petN* (found in 15 species), *psbI* (found in 14 species, except MT239363), *rpl36* (found in 11 species, except MT239365, MT239366, NC_057644, and NC_058233) (Supplementary Table 15). Genes *atpH, ndhH, petL, psaC, psbM, rpl23*, and *rps11* were found all on or above NEC standard curve in 15 *Rhododendron* species chloroplast genomes (Figure 8 and Supplementary Table 14).

**TABLE 3:** Plot of ENC of genes in 5 Ericaceae species mitochondrial genomes.

Genus	Species	Genebank number	ENC plot analysis			
			
			ENC values of gene on or		ENC values of gene below	
			above standard curve line		standard curve line	
				
			Gene number	Gene list	Gene number	Gene list
*Monotropa*	*Monotropa hypopitys*	MK990822	5	*atp4, nad9, rps10, rps19, rps3*	33	*atp1, atp6, atp8, atp9, ccmB, ccmC, ccmFc, ccmFn, cob, cox1, cox2, cox3, matR, mttB, nad1, nad2, nad3, nad4, nad4L, nad5, nad6, nad7, rpl2, rpl5, rpl10, rpl16, rps1, rps4, rps12, rps13, rps19, sdh3, sdh4*
*Arctostaphylos*	*Arctostaphylos glauca*	MZ779111	4	*atp4, nad1, nad9, rpl10*	29	*atp1, atp8, atp9, ccmB, ccmC, ccmFc, ccmFn, cox1, cox2, cox3, cytB, matR, mttB, nad2, nad3, nad4, nad5, nad6, nad7, rpl2, rpl16, rps1, rps4, rps10, rps12, rps13, rps15, rps19, sdh4*
*Rhododendron*	*Rhododendron* × *pulchrum*	OM283814	5	*atp4, atp6, nad9, rpl10, rps12*	28	*atp1, atp8, atp9, ccmB, ccmC, ccmFc, ccmFn, cob, cox1, cox2, cox3, matR, mttB, nad1, nad2, nad3, nad4, nad4L, nad5, nad6, nad7, rpl5, rps1, rps3, rps4, rps14, sdh3, sdh4*
	*Rhododendron simsii*	NC_053763	5	*atp4, atp6, nad9, rpl10, rps12*	28	*atp1, atp8, atp9, ccmB, ccmC, ccmFc, ccmFn, cob, cox1, cox2, cox3, matR, mttB, nad1, nad2, nad3, nad4, nad4L, nad5, nad6, nad7, rpl5, rps1, rps3, rps4, rps14, sdh3, sdh4*
*Vaccinioideae*	*Vaccinium macrocarpon*	NC_023338	4	*atp4, ccmFc, nad9, rpl10*	29	*atp1, atp8, atp9, ccmB, ccmC, ccmFn, cob, cox1, cox2, cox3, matR, mttB, nad1, nad2, nad3, nad4, nad5, nad6, nad7, rpl2, rpl16, rps1, rps4, rps10, rps12, rps13, rps15, rps19*, *sdh4*

### Gene transfer among mitochondrial and chloroplast genomes

The length of the mitochondrial genome sequence (816,410 bp) was found to be approximately 5.6 times longer than the chloroplast genome (146,941 bp) of *R.* × *pulchrum*. Thirteen homologous fragments containing gene sequences were identified between the chloroplast and mitochondrial genomes of *R.* × *pulchrum* (Table 4). The fragments ranged from 68 to 1,799 bp and retained > 70% of their sequence identity with their original chloroplast counterparts. These fragments had a total length of 4,447 bp, accounting for 0.54 and 3.03% of the mitochondrial and chloroplast genomes, respectively. Three mitochondrial genes were identified: *trnM-CAU*, *trnD-GUC*, and *ycf68*. A total of 13 chloroplast genes were identified, including *trnA-UGC*, *trnD-GUC*, *trnI-GAU*, *trnM-CAU*, *trnT-UGU*, *psbF*, *psbL*, *psbD*, *ndhA*, *ndhF*, *rrn16*, *rrn23*, and *ycf68*.

**TABLE 4:** Gene transfer among mitochondrial and chloroplast genomes of *Rhododendron* × *pulchrum.*

Sequence name	Mitochondrial genome		Chloroplast genome		Identity (%)
		
	Gene	Sequence posison (bp)	Gene	Sequence posison (bp)	
S0	*trnM-CAU*	6,870–6,937	*trnT-UGU*	104,61–10,530	72.86
S1	*trnM-CAU*	544,505–544,570	*trnM-CAU*	20,294–20,361	89.71
S2	*trnD-GUC*	98,655–98,779	*trnD-GUC*	97,859–97,983	86.4
S3	/	580,657–580,726	*psbF*	108,602–108,671	90
S4	/	580,735–580,862	*psbF*; *psbL*	108,685–108,812	90.63
S5	*ycf68*	669,585–671,383	*ycf68*; *trnA-UGC* (exon 1); *trnI-GAU* (exon 2); *trnI-GAU* (intron 1); *trnA-UGC* (intron 1)	140,647–142,445	100
S6	/	57,645–75,825	*psbD*	52,426–53,035	99.34
S7	/	478,324–478,417	*ndhF*	73,473–73,566	85.11
S8	/	439,289–439,591	*ndhA* (intron 1)	127,789–128,091	99.67
S9	/	438,679–439,302	*ndhA*; *ndhA* (intron 1)	128,097–128,720	99.68
S10	/	341,227–341,397	*rrn23*	139,234–139,404	95.91
S11	/	71,461–71,598	*rrn16*	143,386–143,523	89.13
S12	/	70,854–71,050	*rrn16*	143,938–144,134	78.17

## Discussion

### Mitochondrial genome structure and size variations

Plant mitochondrial genomes usually have a typical circular structure (Sloan et al., 2018), while in some species, the mitochondrial genomes showed linear or multichromosomal architecture, such as *Silene conica* and *Chenopodium album* (Backert and Borner, 2000; Sloan et al., 2012; Sanchez-Puerta et al., 2017). Plant mitochondrial genomes have a broad distribution in size, usually ranging from 66 to 11,000 kb (Wu et al., 2022). The mitochondrial genome of *R.* × *pulchrum* was identified as a lineage DNA molecule with 816,410 bp. The mitochondrial genome of *R*. *simsii* showed a lineage DNA molecule with 802,707 bp (Xu et al., 2021), while *V*. *macrocarpon* showed a single circular scaffold with 468,115 bp (Luis et al., 2019). In plant taxonomy, *R*. *simsii* and *R.* × *pulchrum* belong to the *Rhododendron* genus of Ericaceae, and *V*. *macrocarpon* belongs to the *Vaccinioideae* of Ericaceae. The size of the assembly mitochondrial genomes for the 11 species of Ericales ranged from 425,282 to 816,410 nucleotides; in the 5 species of Ericaceae, they ranged from 459,678 to 816,410 nucleotides. Previous studies have shown that plant mitochondrial genomes can exhibit wide variations among species (Wu et al., 2022). These results showed that the changes in the structure and size of the mitochondrial genomes of the Ericales species varied widely, which may be related to species evolution and phylogenetic affinities. The structure and size of mitochondrial genomes among the closely related species are closer, such as between *R*. *simsii* and *R.* × *pulchrum* (Xu et al., 2021) and between *C*. *sinensis* and *C*. *sinensis* var. Assamice (Zhang et al., 2019), indicating that the mitochondrial genome may be a potential strategy for studying the evolution of plants and identifying species taxa (Li et al., 2018; Zubaer et al., 2018).

The mitochondrial genome of *R.* × *pulchrum* is the longest genome reported thus far for species of the Ericales, containing 36 protein coding genes, 3 rRNA genes, and 24 tRNA genes, similar to *R*. *simsii* (Xu et al., 2021) and *C*. *sinensis* var. Assamice (Zhang et al., 2019). However, multiple copies of genes are completely different in the mitochondrial genomes of the three different species from Ericales: *R.* × *pulchrum*, *R*. *simsii*, and *C*. *sinensis* var. Assamice. Moreover, there were more annotated genes in the *C*. *sinensis* var. Assamice mitochondrial genome than in *R.* × *pulchrum*, with related genes including *rrn16*, *rpl2*, *rpl16*, *rps7*, *rps13*, *rps19*, and *trnfM*. Multichromosomal genomes in plants have been found in variable copy numbers (Alverson et al., 2011; Wu et al., 2015), while mitochondrial gene copy numbers have no more detailed data, which may be due to the limited data of plant mitochondria until now. The mitochondrial genes *atp1* and *matR* are conserved and have been used to study phylogenetic interrelationships in Ericales (Anderberg et al., 2002). These results were consistent with our study. Thus, plant mitochondrial genomes are needed for further research and will provide novel insights into genome evolution and molecular markers of these diversely structured genomes.

### Chloroplast genome structure and size variations

**FIGURE 9:**
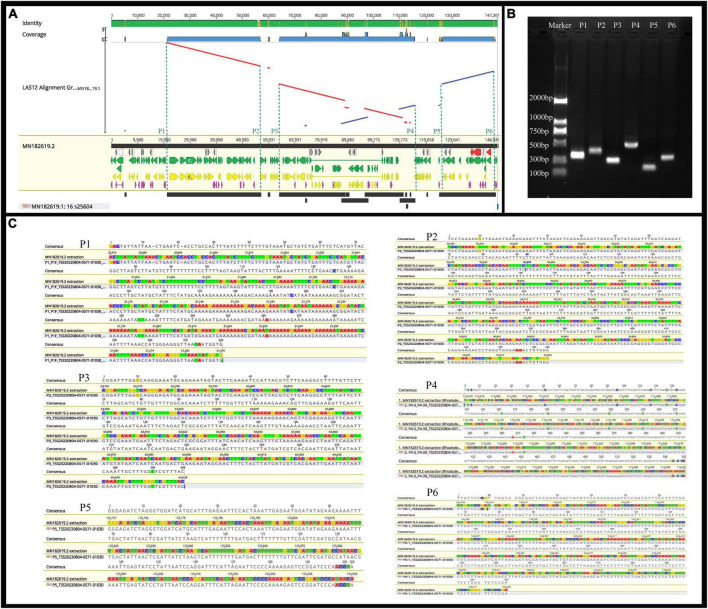
The identified of the results between MN182619.1 and MN182619.2. **(A)** Complete chloroplast genome collinear alignment compared between MN182619.1 and MN182619.2 of *Rhododendron* × *pulchrum*. MN182619.1 as the reference; blue bars represent homologous high-scoring segment pairs in a codirectional orientation, whereas red bars represent reversed pairs. Yellow arrows represent coding sequences, red arrows represent ribosomal RNA genes, purple arrows represent transfer RNA genes, green arrows represent protein-coding genes, and gray arrows represent exonic regions. Black squares represent homology; the larger the number, the larger the area of the black squares, and the more closely related species have homologous fragments. Red wireframes are the most conservative regions. The P1∼P6 sites located in 21,232, 56,637, 63,979, 115,230, 125,853, and 145,493 bp position of MN182619.2. **(B)** PCR products detection. (The P1∼P6 sites located in 21,232, 56,637, 63,979, 115,230, 125,853, and 145,493 bp position of MN182619.2; the expected size of the P1∼P6 are 355, 444, 300, 581, 201, and 348 bp, respectively). **(C)** Results of PCR compared with plastid genomes fasta (MN182619.2) that assembled with third-generation combined with second-generation sequencing methods. [A∼E (P1∼P6): Six broken sites were randomly selected from the result of collinearly aligned (LASTZ) of MN182619.1 and MN182619.2, as shown in Figure 5. The upper numbers indicate PCR results, the lower numbers indicate the point of plastid genomes fasta that assembled with third-generation combined with second-generation sequencing methods].

Previous studies have confirmed that whole chloroplast genome sequencing data using second-generation sequencing technology (Illumina Hiseq Platform) are reliable (Zhang et al., 2019; Xu et al., 2021). Using second-generation sequencing technology, the complete chloroplast genome of *R.* × *pulchrum* was found to be 136,249 bp in length, without a typical quadripartite structure due to missing inverted repeats, and the GC content was 35.98%. In total, 73 functional genes were identified, including 2 rRNA genes, 29 tRNA genes, and 42 protein-coding genes (Shen et al., 2020). In this study, second- and TGS technologies were used to reassemble and reannotate the complete chloroplast genome of *R.* × *pulchrum*. Our new results showed that the complete *R.* × *pulchrum* chloroplast genome was 146,941 bp in length (Figure 5); it lacked a typical quadripartite structure, and the GC content showed no significant change compared with the results of previous research. However, the complete chloroplast genome of *R.* × *pulchrum* contained 119 annotated genes; 46 more genes were annotated than in previous studies, including 5 tRNA genes, 2 rRNA genes, and 39 protein-coding genes (mRNA) (Table 1). The previously published plastid genome of *R.* × *pulchrum* was sequenced with the Illumina Hiseq Platform (Shen et al., 2019, 2020). In this study, we used TGS methods combined with second-generation sequencing technology to detect the complete plastid genomes of *R.* × *pulchrum*. The sequencing results of the two methods were compared using Lastz software for investing the accuracy of the results. Complete chloroplast genome collinear alignment compared between MN182619.1 and MN182619.2 of *Rhododendron* × *pulchrum* had been shown in Figure 9A. The comparison results found there have multiple breakage and inversion sites, 6 of this breakage sites with long segment (10,000–30,000 bp) were selected to design primers for PCR experiments (Supplementary Table 16). The PCR results meet the expected size (Figure 9B) and the sequencing results were consistent with MN182619.2 (Figure 9C). Our study illustrated that TGS technology (Oxford Nanopore Platform) could improve the accuracy of coverage and assembly of previously unassembled genomic regions, and guarantee the accuracy of the length and content of the genomes. Third-generation sequencing technology may be a useful tool to explain plant organelle genome information (Shearman et al., 2016) for TGS methods with a long-read length.

According to the published data, the plastid genomes of most plants are display the typical quadripartite structure by showing the LSC, LSC, and two IRs (Wicke et al., 2011; Shen et al., 2020). Our results indicate that the *R.* × *pulchrum* cp genome lacks the IRs, this is inconsistent with *Rhododendron delavayi* (Li et al., 2020). The plastid genomes of *Rhodordendron* species are display different structure. These illustrating that the plastid genome may be a potential strategy for studying the evolution and identifying species taxa of *Rhodordendron* species (Li et al., 2018; Zubaer et al., 2018).

### Phylogenetic and mitochondrial genome comparison

The mitochondrial and chloroplast phylogenetic trees of 8 species from Ericales and 15 species from *Rhododendron* showed results consistent with their taxonomic information. These results illustrate that organelle genome sequence data are suitable for studying phylogeny in plants (Wu and Ge, 2012; Thomas et al., 2017; Shen et al., 2020). The phylogenetic trees in this study showed that *R. ripense* was a sister to *R.* × *pulchrum* (Figures 2, 6). Previous studies indicated *R.* × *pulchrum* cultivar owned cpDNA of *R. ripense* (Shirasawa et al., 2021), and *R. ripense* is considered as one of the putative ancestral species of *R.* × *pulchrum* (Scariot et al., 2007; Meanchaipiboon et al., 2021), These findings are consistent with our study. The *R.* × *pulchrum* mitochondrial genome was used as a reference; a whole-genome collinear alignment compared among five species from the Ericaceae family found a high degree of homology with *R.* × *pulchrum* in the gene regions compared with interval regions. This illustrates that gene regions are more conserved than interval regions in the mitochondrial genomes of the Ericaceae species. The 11 conservative genes (*atp4*, *ccmC*, *nad4*, *trnC-GCA*, *trnN-GUU*, *trnY-GUA*, *nad2*, *nad5*, *trnM-CAU*, *trnD-GUC*, and *rrn26*) identified in this study will be useful for understanding the evolutionary mechanisms and identifying species taxa in Ericaceae (Li et al., 2018; Zubaer et al., 2018).

The mitochondrial genomes of *R.* × *pulchrum* and *R*. *simsii* were identified as 63 LCBs; these LCBs differed substantially in size and relative position (Figure 4). Moreover, most LCBs contain gene sequences. Our results indicate that gene sizes are not significantly different (Xu et al., 2021). Although many genes have rearrangements between *R.* × *pulchrum* and *R*. *simsii* mitochondrial genomes, there are very complex variations in their mitochondrial genomes, which may be resulting in hybridization and genome doubling events. These results are consistent with a previous study that showed high variation and recombination in plant mitochondrial genomes (Galtier, 2011). Moreover, gene order in plant mitochondrial genomes is highly variable (Fischer et al., 2006; Schoch et al., 2015; Li et al., 2017). The arrangement of genes in mitochondria may be used to assess phylogenetic relationships among different species (Li et al., 2017).

### Codon usage bias patterns and evolution

Codon usage bias are usually used in animals and insects to analysis phylogenetic and evolution of different species, however, there are few reports on plants (Sun et al., 2009; Huang and Ma, 2018). The gene codon usage bias of mitochondrial chloroplast genes with low ENC values (ENC < 35; Supplementary Tables 13, 14) maybe caused by mutation, while the codon usage bias of the other genes are caused by choice or other factors (Wright, 1990). Thus, we can infer the gene codon usages bias of only 10 Ericaceae mitochondrial genes (*atp4, atp6, ccmFc, nad1, nad9, rpl10, rps3, rps10, rps12*, and *rps19*) and 7 *Rhododendron* chloroplast genes (*atpH, ndhH, petL, psaC, psbM, rpl23*, and *rps11*) were influenced by mutation, while other genes codon usage bias had undergone natural or artificial selection. NEC plot analysis of mitochondrial genes showed the codon usages of the genes between *R.* × *pulchrum* and *R. simsii* are consistent, the gene codon usages in *Rhododendron* species from the same subgenus or subsection species in chloroplasts genomes are also consistent. These results showed NEC plot analysis of mitochondrial and chloroplast genomes maybe suitable for evolutionary analysis of Ericaceae plants.

### Gene transfer between mitochondrial and chloroplast genomes

Previous research has detected intracellular gene transfer between different genomes, which has been disclosed through sequencing analysis of nuclear, mitochondrial, and chloroplast genomes (Timmis et al., 2004; Nguyen et al., 2020). Most of these studies focused on the gene transfer of nuclear DNA from the organelle in angiosperms (Smith, 2011; Park et al., 2014). With the development of new techniques, increasing organelle genome data have been published and analyzed, and chloroplast-to-mitochondrial gene transfer has been considered a characteristic feature of long-term evolution (Gui et al., 2016; Nguyen et al., 2020). In this study, 13 homologous fragments containing gene sequences were identified between the chloroplast and mitochondrial genomes of *R.* × *pulchrum* (Table 4), which may be the result of horizontal gene transfer between organelle genomes. In mitochondrial genomes of *R.* × *pulchrum*, 10 chloroplast genes have not been annotated, including 3 tRNA (*trnA-UGC*, *trnI-GAU*, and *trnT-UGU*), 2 ribosomal genes (*rrn16* and *rrn23*), 3 photosynthetic genes (*psbF*, *psbL*, and *psbD*), and 2 NADH dehydrogenase genes (*ndhA* and *ndhF*), but contain their homologous fragments (Table 4). Ribosomal genes participate in the synthesis of the ribosomal complex, which participates in the proteins required for normal cell function (Depamphilis and Palmer, 1990; Wang et al., 2019). The photosynthetic genes and NADH dehydrogenase genes participate in the synthesis of photosynthetic system II and chloroplast NADH dehydrogenase complex, which are involved in photosynthesis (Sazanov et al., 1998; Cai et al., 2021). It is speculated that these genes related to photosynthesis, such as *psbF*, *psbL*, *psbD*, *ndhA*, and *ndhF*, may be the result of chloroplast genes transferred to mitochondria in *R.* × *pulchrum*. Further research on plant mitochondrial and chloroplast genomes is needed and will provide novel insight into genome evolution, phylogenetic relationships, and molecular markers of these diversely structured genomes.

## Conclusion

This study detected the complete mitochondrial genome and reassembled the chloroplast genome of *R.* × *pulchrum*. Genome organization features, genome comparison with related species, and gene transfer among mitochondrial and chloroplast genomes were researched. Phylogenetic relationships, codon usage bias patterns and evolution were compared with related species. Our results *R.* × *pulchrum* revealed that the organelles genome variation of Ericaceae species is very complex. Our results will be useful for understanding the evolutionary mechanisms and identifying species taxa in Ericaceae.

## Data availability statement

The datasets presented in this study can be found in online repositories. The names of the repository/repositories and accession number(s) can be found in the article/Supplementary material.

## Author contributions

JS and SJ: conceptualization, validation, resources, data curation, and writing—original draft preparation, review and editing. XL and ML: methodology. ML, XH, and HC: software. SJ: funding acquisition. All authors have read and agreed to the published version of the manuscript.

## Footnotes

1
https://github.com/rrwick/Porechop
