# Supplementary material for: Characterization, comparative phylogenetic, and gene transfer analyses of organelle genomes of Rhododendron × pulchrum
Source: Front Plant Sci. 2022 Sep 21;13:969765. doi: 10.3389/fpls.2022.969765 (PMC9532937; doi:10.3389/fpls.2022.969765)
Supplement: Supplementary file 1 [file Data_Sheet_1.docx]

Supplementary Material

# Supplementary Figures and Tables

**
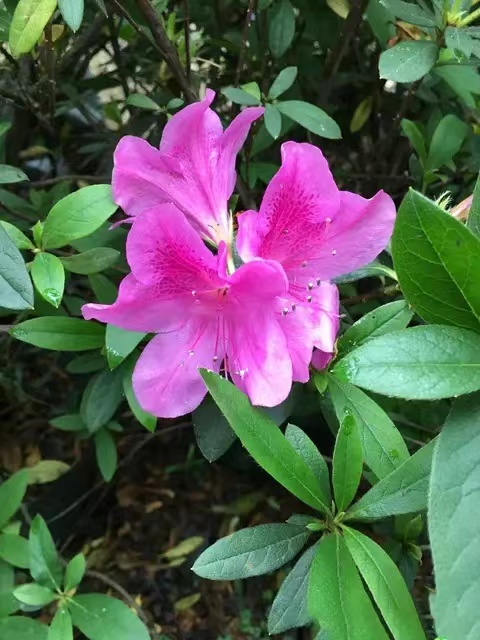
**

**Supplementary Figure 1.** *Rhododendron × pulchrum* cultivars with purple large flowers.

**
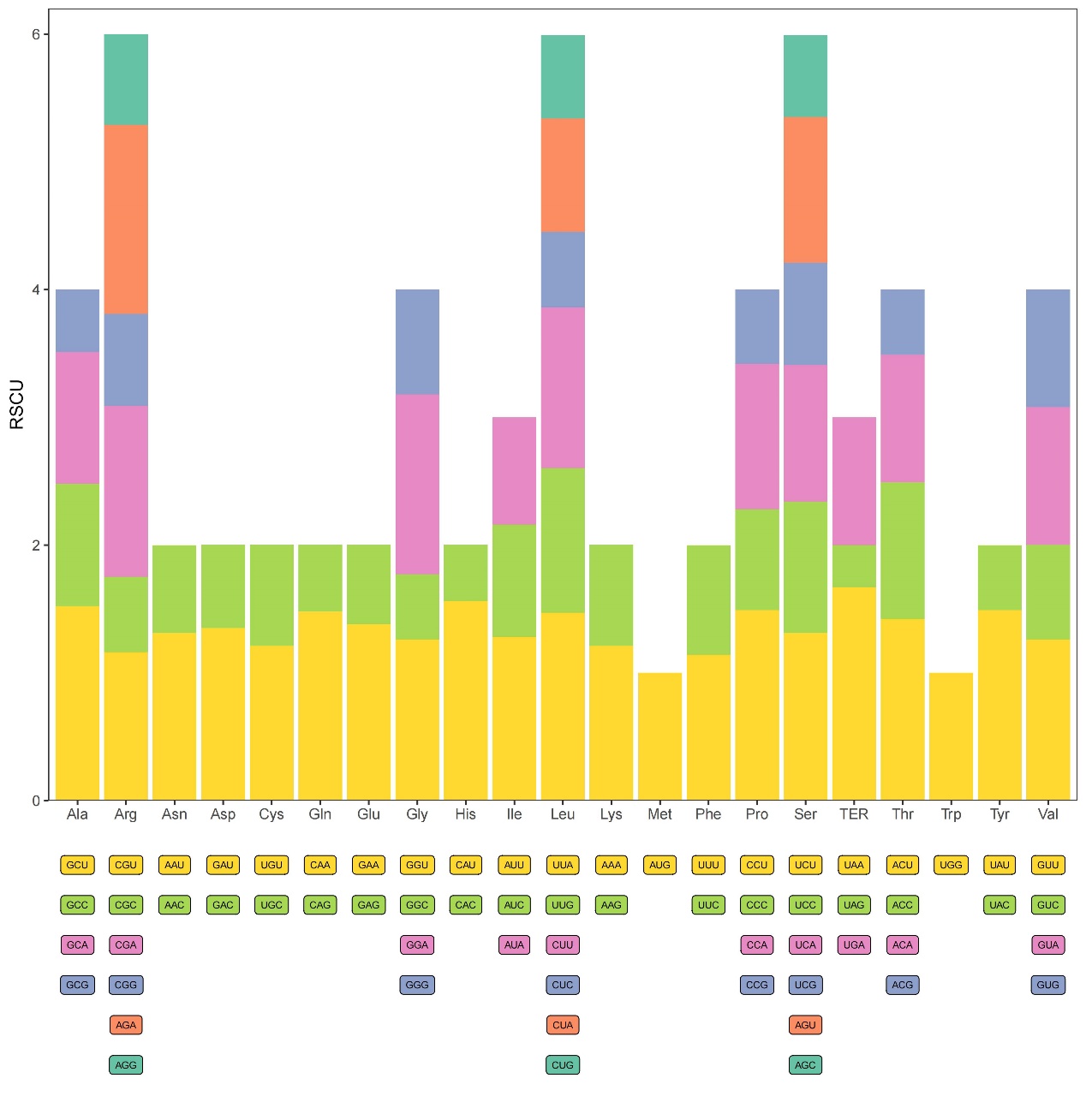
**

**Supplementary Figure 2.** Codon usage of the *Rhododendron × pulchrum* mitochondrial genome.

(RSCU, relative synonymous codon usage value)

**
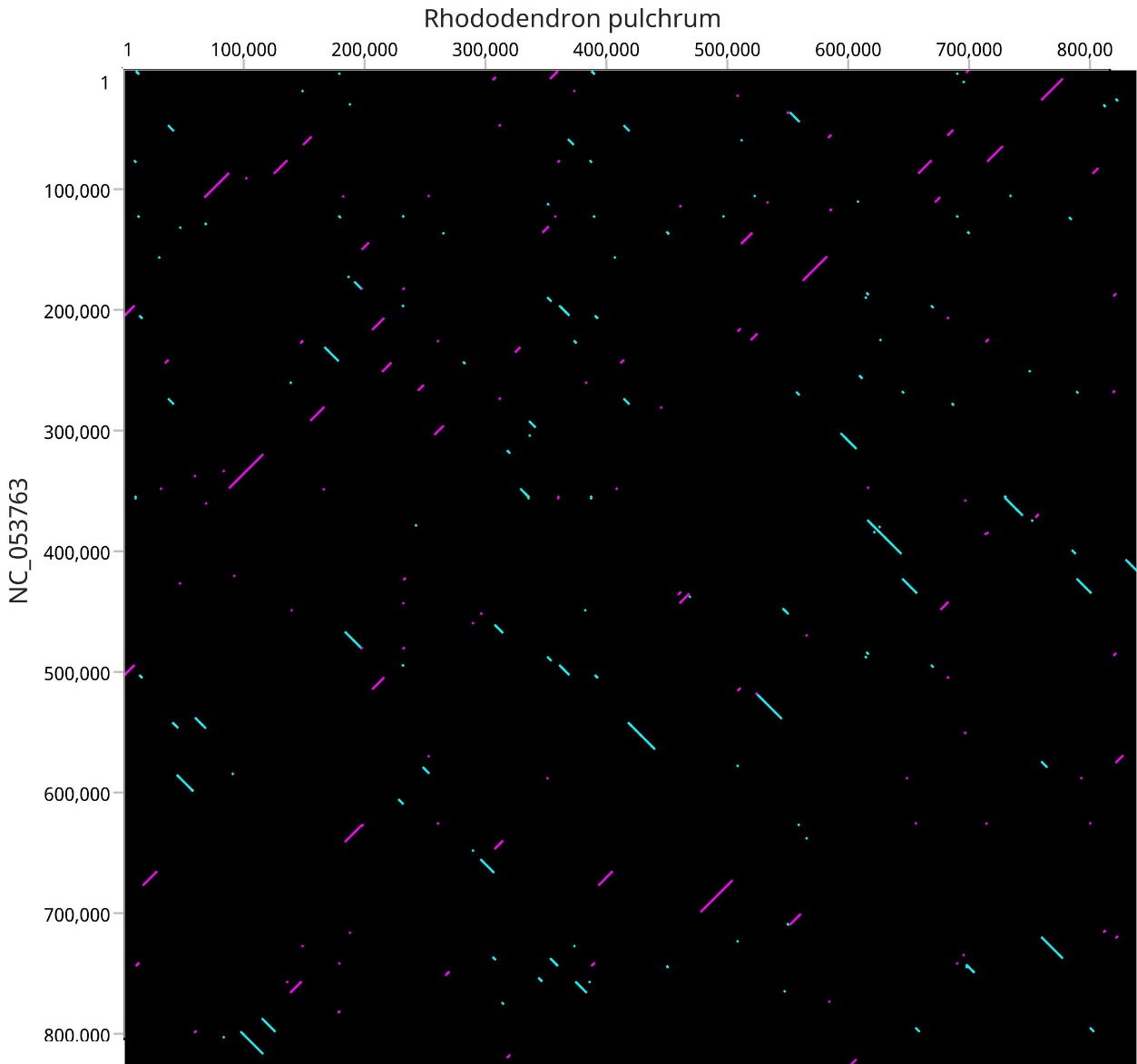
 Supplementary Figure 3.** Complete mitochondrial genome collinearity analysis between *Rhododendron × pulchrum* and *R*. *simsii*.

Blue bars represent homologous high-scoring segment pairs in a codirectional orientation, whereas purple bars represent reversed pairs.

**
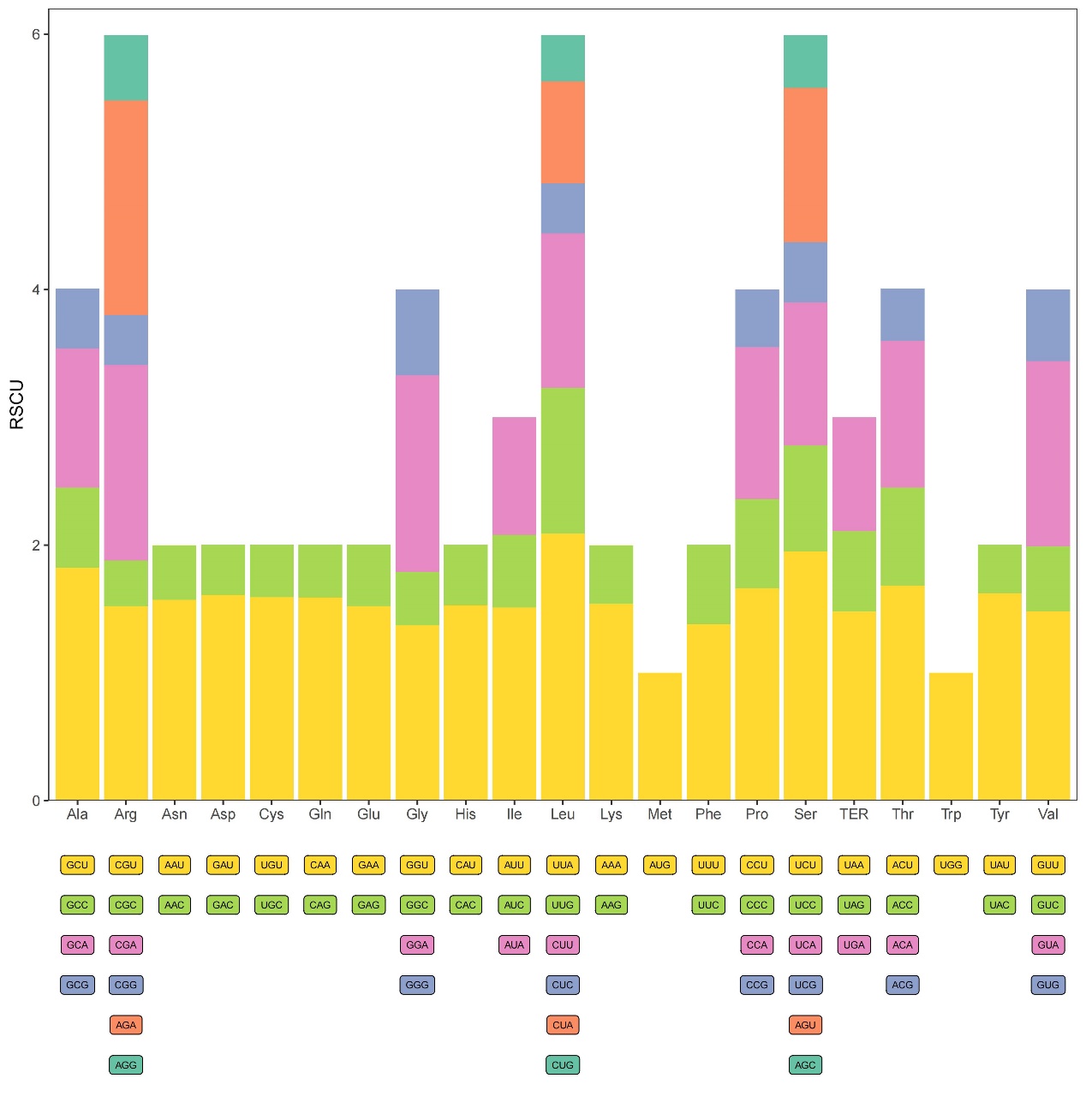
 Supplementary Figure 4.** Codon usage of the *Rhododendron × pulchrum* chloroplast genome.

(RSCU, relative synonymous codon usage value)

| **Sample name** | **Next-generation sequencing platform** | | | | | **Third-generation sequencing platform** | | | | | |
| --- | --- | --- | --- | --- | --- | --- | --- | --- | --- | --- | --- |
|  | **Clean reads (bp)** | **Clean bases** | **Read length (bp)** | **Q30 (%)** | **GC (%)** | **Total pass reads** | **Total pass bases (GB)** | **Maxlength (bp)** | **Meanlength (bp)** | **N50 length (bp)** | **Mean QV** |
| *Rhododendron × pulchrum* | 36,533,204 | 5,479,980,600 | 150 | 93.18 | 39.60% | 5,364,975 | 50.883 | 438,985 | 9,484.35 | 29,463 | 11.83 |

**Supplementary Table 3.** Summary of Sequencing statistics

**Supplementary Table 4.** Base composition of the *Rhododendron × pulchrum* mitochondrial genome.

| **Statistics** | **A** | **T (U)** | **C** | **G** | **G + C** | **Total** |
| --- | --- | --- | --- | --- | --- | --- |
| Frequency | 221,453 | 221,801 | 187,046 | 186,110 | 373,156 |  |
| Percentage （%） | 27.10 | 27.20 | 22.90 | 22.80 | 45.70 | 100.00 |

**Supplementary Table 5.** Codon-anticodon recognition patterns and codon usage of the *Rhododendron × pulchrum* mitochondrial genome.

| **Amino Acid** | **Codon** | **Percentage of the same acid** (%) | **Percentage of all codon (%)** | **Frequency** | **relative synonymous codon usage value (RSCU)** |
| --- | --- | --- | --- | --- | --- |
| Ala | GCA | 25.70 | 1.63 | 182 | 1.03 |
| Ala | GCC | 24.00 | 1.52 | 170 | 0.96 |
| Ala | GCG | 12.30 | 0.78 | 87 | 0.49 |
| Ala | GCU | 37.90 | 2.39 | 268 | 1.52 |
| Cys | UGC | 39.30 | 0.57 | 64 | 0.79 |
| Cys | UGU | 60.70 | 0.88 | 99 | 1.21 |
| Asp | GAC | 32.40 | 0.98 | 110 | 0.65 |
| Asp | GAU | 67.60 | 2.05 | 229 | 1.35 |
| Glu | GAA | 69.00 | 2.82 | 316 | 1.38 |
| Glu | GAG | 31.00 | 1.27 | 142 | 0.62 |
| Phe | UUC | 43.00 | 3.06 | 343 | 0.86 |
| Phe | UUU | 57.00 | 4.06 | 454 | 1.14 |
| Gly | GGA | 35.30 | 2.52 | 282 | 1.41 |
| Gly | GGC | 12.70 | 0.90 | 101 | 0.51 |
| Gly | GGG | 20.60 | 1.46 | 164 | 0.82 |
| Gly | GGU | 31.50 | 2.24 | 251 | 1.26 |
| His | CAC | 21.80 | 0.55 | 62 | 0.44 |
| His | CAU | 78.20 | 1.99 | 223 | 1.56 |
| Ile | AUA | 28.10 | 2.22 | 248 | 0.84 |
| Ile | AUC | 29.40 | 2.32 | 260 | 0.88 |
| Ile | AUU | 42.50 | 3.36 | 376 | 1.28 |
| Lys | AAA | 60.50 | 2.29 | 256 | 1.21 |
| Lys | AAG | 39.50 | 1.49 | 167 | 0.79 |
| Leu | CUA | 14.90 | 1.72 | 193 | 0.89 |
| Leu | CUC | 9.90 | 1.15 | 129 | 1.16 |
| Leu | CUG | 10.90 | 1.26 | 141 | 0.65 |
| Leu | CUU | 21.10 | 2.45 | 274 | 1.26 |
| Leu | UUA | 24.60 | 2.85 | 319 | 1.47 |
| Leu | UUG | 18.60 | 2.16 | 242 | 1.13 |
| Met | AUG | 100.00 | 2.77 | 310 | 1 |
| Asn | AAC | 34.30 | 1.06 | 119 | 0.69 |
| Asn | AAU | 65.70 | 2.04 | 228 | 1.31 |
| Pro | CCA | 28.40 | 1.43 | 160 | 1.14 |
| Pro | CCC | 19.70 | 0.99 | 111 | 0.79 |
| Pro | CCG | 14.60 | 0.73 | 82 | 0.58 |
| Pro | CCU | 37.30 | 1.88 | 210 | 1.49 |
| Gln | CAA | 74.10 | 1.95 | 218 | 1.48 |
| Gln | CAG | 25.90 | 0.68 | 76 | 0.52 |
| Arg | AGA | 24.60 | 1.46 | 164 | 1.48 |
| Arg | AGG | 11.80 | 0.71 | 79 | 0.71 |
| Arg | CGA | 22.30 | 1.33 | 149 | 1.34 |
| Arg | CGC | 9.90 | 0.59 | 66 | 0.59 |
| Arg | CGG | 12.00 | 0.71 | 80 | 0.72 |
| Arg | CGU | 19.30 | 1.15 | 129 | 0.59 |
| Ser | AGC | 10.70 | 0.96 | 107 | 0.64 |
| Ser | AGU | 19.00 | 1.70 | 190 | 1.08 |
| Ser | UCA | 17.80 | 1.59 | 178 | 1.07 |
| Ser | UCC | 17.20 | 1.54 | 172 | 1.03 |
| Ser | UCG | 13.40 | 1.20 | 134 | 0.8 |
| Ser | UCU | 21.90 | 1.96 | 219 | 1.31 |
| Thr | ACA | 24.90 | 1.28 | 143 | 1 |
| Thr | ACC | 26.80 | 1.38 | 154 | 1.07 |
| Thr | ACG | 12.70 | 0.65 | 73 | 0.51 |
| Thr | ACU | 35.50 | 1.82 | 204 | 1.42 |
| Val | GUA | 27.00 | 1.70 | 190 | 1.14 |
| Val | GUC | 18.40 | 1.16 | 130 | 0.74 |
| Val | GUG | 23.10 | 1.46 | 163 | 0.92 |
| Val | GUU | 31.50 | 1.98 | 222 | 1.26 |
| Trp | UGG | 100.00 | 1.56 | 175 | 1 |
| Tyr | UAC | 25.70 | 0.85 | 95 | 0.51 |
| Tyr | UAU | 74.30 | 2.45 | 274 | 1.49 |
| TER | TAA | 55.60 | 0.18 | 20 | 1.67 |
| TER | TAG | 11.10 | 0.04 | 4 | 0.33 |
| TER | TGA | 33.30 | 0.11 | 12 | 1 |
| Total |  |  | 100.00 | 11196 |  |

**Supplementary Table 6.** Simple sequence repeats in the *Rhododendron × pulchrum* mitochondrial genome

| **Sequences** | **Number of repeats** | | | | | | | | | | |
| --- | --- | --- | --- | --- | --- | --- | --- | --- | --- | --- | --- |
|  | **5** | **6** | **7** | **8** | **9** | **10** | **11** | **12** | **13** | **14** | **total** |
| A/T | - | - | - | - | - | 22 | 7 | 3 | 1 | 1 | 34 |
| C/G | - | - | - | - | - | 1 | 1 | - | - | - | 2 |
| AG/CT | - | 3 | 1 | - | - | - | - | - | - | - | 4 |
| AAG/CTT | 2 | - | - | - | - | - | - | - | - | - | 2 |

**Supplementary Table 7.** Tandem repeats identified from the *Rhododendron × pulchrum* mitochondrial genome

| **Indices** | **PeriodSize** | **CopyNumber** | **ConsensusSize** | **PercentMatches** | **PercentIndels** | **Score** | **A** | **C** | **G** | **T** | **Entropy (0-2)** | **Sequence 1** | **Sequence 2** |
| --- | --- | --- | --- | --- | --- | --- | --- | --- | --- | --- | --- | --- | --- |
| 84080-84111 | 16 | 2 | 16 | 93 | 0 | 55 | 9 | 25 | 28 | 37 | 1.87 | GTTGCCCTATAGTTGC | GTTGCCCTATAGTTGCGTTGCCCTGTAGTTGC |
| 141865-141929 | 21 | 3.1 | 21 | 66 | 24 | 55 | 15 | 35 | 9 | 40 | 1.79 | TCAGCATTCTCCTTCCGCATT | TCAGCATTCTCCTTCCTCATTTCCGCAATCATCTGCTTGATTTCAGCATTCTCCTTCCGCATTTC |
| 141865-141947 | 42 | 2 | 42 | 97 | 0 | 157 | 16 | 33 | 10 | 38 | 1.84 | TCAGCATTCTCCTTCCGCATTTCCGCAATCATCTGCTTGATT | TCAGCATTCTCCTTCCTCATTTCCGCAATCATCTGCTTGATTTCAGCATTCTCCTTCCGCATTTCCGCAATCATCTGCTTGAT |
| 142034-142060 | 13 | 2.1 | 13 | 100 | 0 | 54 | 70 | 7 | 22 | 0 | 1.12 | AAGACGAAAGAAA | AAGACGAAAGAAAAAGACGAAAGAAAA |
| 156460-156492 | 15 | 2.1 | 16 | 88 | 5 | 50 | 27 | 27 | 30 | 15 | 1.96 | TCATAGCAGCGAGAGC | TCATAGCAGCGAGAGCTCATAGCCGGAGAGCTC |
| 172327-172362 | 16 | 2.2 | 16 | 90 | 0 | 54 | 41 | 27 | 0 | 30 | 1.56 | CTCATAACTCAACTAA | CTCATAACTCAACTAACTCATAATTCAATTAACTCA |
| 180817-180870 | 28 | 1.9 | 28 | 85 | 7 | 74 | 18 | 11 | 46 | 24 | 1.81 | GAGTGAGCTAGGTGAGTAGCTCGAGTTG | GAGTGAGCAGGGTGGGTAGCTCGAGTTGGAGTGAGCTAGGTGAGTTGCTCGAGT |
| 221749-221773 | 12 | 2.1 | 12 | 100 | 0 | 50 | 0 | 32 | 28 | 40 | 1.57 | GTTCCTGTGCCT | GTTCCTGTGCCTGTTCCTGTGCCTG |
| 273021-273050 | 12 | 2.5 | 12 | 94 | 0 | 51 | 30 | 20 | 30 | 20 | 1.97 | CAGCTATAAGGG | CAGCTATAAGGGCAGCTATAAGGGCTGCTA |
| 359809-359835 | 13 | 2.1 | 13 | 100 | 0 | 54 | 0 | 22 | 7 | 70 | 1.12 | TTTTCTTTCGTCT | TTTTCTTTCGTCTTTTTCTTTCGTCTT |
| 359922-360004 | 42 | 2 | 42 | 97 | 0 | 157 | 38 | 10 | 33 | 16 | 1.84 | ATCAAGCAGATGATTGCGGAAATGAGGAAGGAGAATGCTGAA | ATCAAGCAGATGATTGCGGAAATGCGGAAGGAGAATGCTGAAATCAAGCAGATGATTGCGGAAATGAGGAAGGAGAATGCTGA |
| 359922-359986 | 21 | 3.1 | 21 | 68 | 24 | 64 | 38 | 12 | 30 | 18 | 1.88 | ATCAAGCAGATGATTGCGGAA | ATCAAGCAGATGATTGCGGAAATGCGGAAGGAGAATGCTGAAATCAAGCAGATGATTGCGGAAAT |
| 417731-417775 | 17 | 2.7 | 17 | 86 | 3 | 56 | 37 | 24 | 6 | 31 | 1.81 | ACAACCTATTCTATGAA | ACAACCTATTCGATTAAACAACCTATTCTATGAAAGAACCTTTCT |
| 428945-428994 | 16 | 3.1 | 16 | 91 | 0 | 82 | 44 | 24 | 16 | 16 | 1.86 | TAGCAACAGGAACTAC | TAGCAACAAGAACTACTAGCTACAGGAACTACTAGCAACAGGAACTACTA |
| 433225-433303 | 29 | 2.7 | 29 | 94 | 0 | 131 | 39 | 26 | 7 | 26 | 1.83 | GACTAGTAATTCCAAAACCAATTCACTCC | GACTAGTAATTCCAAATTCAATTCACTCCGACTAGTAATTCCAAAACCAATTCACTCTGACTAGTAATTCCAAAACCAA |
| 465657-465694 | 20 | 1.9 | 19 | 89 | 5 | 58 | 18 | 23 | 21 | 36 | 1.95 | TCAGTTCAAGACTTGGTTC | TCAGTTCAAGACTTCGGTTCTCAGTTCACGACTTGGTT |
| 495772-495869 | 24 | 4.1 | 24 | 74 | 2 | 72 | 13 | 31 | 24 | 30 | 1.93 | CTGCCCTTGATCGCACTCATCGAG | CTGCCCTTGATTGGACTGATCGAGCTGCTCTTGGTCGCACTCATCGAGCTGCCCTTCTTCGGAGTCATGGAACTGCCCTTGTTCGTCCTCATCGAGCT |
| 496161-496255 | 48 | 2 | 48 | 82 | 0 | 118 | 21 | 26 | 30 | 22 | 1.98 | TCGGACACATCGAGCAGGCCTTGTCCAGACATCAGGAGCGTACCATGG | TCGGACAGATCGAGCAGGCCTTGTCTAGTCATCAGGAGCGTACCATGGTCGGACTCATGGAGCTGGCCTTGTCCAGACGTCAGGAGCTTACCATG |
| 600673-600707 | 14 | 2.5 | 14 | 95 | 0 | 61 | 34 | 28 | 11 | 25 | 1.91 | TCAACTCAACGATG | TCAACTCAATGATGTCAACTCAACGATGTCAACTC |
| 605529-605607 | 39 | 2 | 39 | 97 | 0 | 149 | 31 | 18 | 27 | 21 | 1.97 | AATATCATGATCGGGTCGACCAGGCCAGATCATGAGTGA | AATATCATGATTGGGTCGACCAGGCCAGATCATGAGTGAAATATCATGATCGGGTCGACCAGGCCAGATCATGAGTGAA |
| 618833-618879 | 18 | 2.5 | 19 | 83 | 13 | 62 | 12 | 25 | 12 | 48 | 1.77 | CTAGTAGCTTTTTCTTCCT | CTAGTAGCTTTCTTCTTTCCTAGTAGCTTTTCTTCCTCTAGTAGCTT |
| 650606-650649 | 20 | 2.3 | 19 | 84 | 11 | 54 | 22 | 22 | 2 | 52 | 1.58 | TTTACTTAACTGACTCTTT | TTTACTAACTGACATCTTTCTTTACTTAACTTACTCTTTTTTAC |
| 651895-651927 | 17 | 1.9 | 17 | 100 | 0 | 66 | 54 | 18 | 12 | 15 | 1.71 | AAAAAACCCATTGGAAT | AAAAAACCCATTGGAATAAAAAACCCATTGGAA |
| 729155-729192 | 17 | 2.2 | 17 | 100 | 0 | 76 | 18 | 18 | 34 | 28 | 1.95 | AGCTTTCATTGGCGGGA | AGCTTTCATTGGCGGGAAGCTTTCATTGGCGGGAAGCT |
| 730100-730146 | 15 | 3.1 | 15 | 100 | 0 | 94 | 27 | 21 | 38 | 12 | 1.9 | ACAGTGTCAGGCGGA | ACAGTGTCAGGCGGAACAGTGTCAGGCGGAACAGTGTCAGGCGGAAC |
| 742682-742724 | 18 | 2.4 | 18 | 81 | 18 | 54 | 18 | 20 | 25 | 34 | 1.96 | CATAGTCGCGAGCTGTTT | CATAGTCGCGAGTCTGTTTATAGTCGCGACTGTTGTCATAGTC |
| 758397-758443 | 18 | 2.5 | 19 | 83 | 13 | 62 | 12 | 25 | 12 | 48 | 1.77 | CTAGTAGCTTTTTCTTCCT | CTAGTAGCTTTCTTCTTTCCTAGTAGCTTTTCTTCCTCTAGTAGCTT |
| 796667-796691 | 6 | 4.2 | 6 | 100 | 0 | 50 | 32 | 20 | 16 | 32 | 1.94 | CAGTAT | CAGTATCAGTATCAGTATCAGTATC |

**Supplementary Table 8.** The genes information contained in locally collinear blocks in mitochondrial genomes of Rhododendron × pulchrum

| ***Rhododendron ×*** mitogenome pot / start (bp) -end (bp) | **Gene** |
| --- | --- |
| 42041-54214 | *nad1* |
| 42041-54214 | *matR* |
| 64215-82776 | *rrn18* |
| 64215-82776 | *rrn5* |
| 83843-102496 | *nad4L* |
| 83843-102496 | *atp4* |
| 110120-119891 | *trnH-GUG* |
| 110120-119891 | *trnE-UUC* |
| 163391-170431 | *nad7* |
| 189678-190318 | *trnM-CAU* |
| 248459-254539 | *cob* |
| 248459-254539 | *rps14* |
| 248459-254539 | *rpl5* |
| 285121-295004 | *trnC-GCA* |
| 285121-295004 | *trnN-GUU* |
| 285121-295004 | *trnY-GUA* |
| 285121-295004 | *nad2* |
| 324006-327710 | *rps3* |
| 334768-338539 | *nad1* |
| 406026-425596 | *nad5* |
| 406026-425596 | *ccmFN* |
| 461302-485735 | *nad6* |
| 461302-485735 | *rps4* |
| 501490-505600 | *cox2* |
| 505609-525191 | *trnI-CAU* |
| 505609-525191 | *trnK-UUU* |
| 533266-541007 | *ccmB* |
| 533266-541007 | *rpl10* |
| 543082-561484 | *trnM-CAU* |
| 543082-561484 | *trnD-GUC* |
| 543082-561484 | *atp6* |
| 573400-584933 | *nad2* |
| 573400-584933 | *sdh3* |
| 594764-620945 | *nad1* |
| 594764-620945 | *trnS-GCU* |
| 594764-620945 | *trnF-GAA* |
| 594764-620945 | *trnP-UGG* |
| 653256-658476 | *ccmFC* |
| 673794-679383 | *atp1* |
| 689472-690682 | *rps12* |
| 689472-690682 | *nad3* |
| 704531-718048 | *atp8* |
| 704531-718048 | *cox3* |
| 704531-718048 | *sdh4* |
| 704531-718048 | *trnG-GCC* |
| 704531-718048 | *trnQ-UUG* |
| 801449-816410 | *trnS-UGA* |

**Supplementary Table 9.** Codon-anticodon recognition patterns and codon usage of the *Rhododendron × pulchrum* chloroplast genome.

| **Amino Acid** | **Codon** | **Percentage of the same acid (%)** | **Percentage of all codon (%)** | **Frequency** | **relative synonymous codon usage value (RSCU)** |
| --- | --- | --- | --- | --- | --- |
| Ala | GCA | 27.2 | 1.69 | 325 | 1.09 |
| Ala | GCC | 15.7 | 0.98 | 188 | 0.63 |
| Ala | GCG | 11.6 | 0.72 | 139 | 0.47 |
| Ala | GCU | 45.4 | 2.82 | 543 | 1.82 |
| Cys | UGC | 20.5 | 0.23 | 44 | 0.41 |
| Cys | UGU | 79.5 | 0.89 | 171 | 1.59 |
| Asp | GAC | 19.5 | 0.72 | 139 | 0.39 |
| Asp | GAU | 80.5 | 2.98 | 574 | 1.61 |
| Glu | GAA | 76.2 | 3.87 | 746 | 1.52 |
| Glu | GAG | 23.8 | 1.21 | 233 | 0.48 |
| Phe | UUC | 31.2 | 1.75 | 338 | 0.62 |
| Phe | UUU | 68.8 | 3.86 | 745 | 1.38 |
| Gly | GGA | 38.4 | 2.82 | 543 | 1.54 |
| Gly | GGC | 10.6 | 0.78 | 150 | 0.42 |
| Gly | GGG | 16.7 | 1.22 | 236 | 0.67 |
| Gly | GGU | 34.3 | 2.51 | 484 | 1.37 |
| His | CAC | 23.5 | 0.53 | 103 | 0.47 |
| His | CAU | 76.5 | 1.74 | 335 | 1.53 |
| Ile | AUA | 30.6 | 2.66 | 512 | 0.92 |
| Ile | AUC | 19.3 | 1.66 | 320 | 0.57 |
| Ile | AUU | 50.3 | 4.36 | 841 | 1.51 |
| Lys | AAA | 77.1 | 4.00 | 772 | 1.54 |
| Lys | AAG | 22.9 | 1.19 | 229 | 0.46 |
| Leu | CUA | 13.4 | 1.43 | 276 | 0.8 |
| Leu | CUC | 6.6 | 0.70 | 135 | 0.39 |
| Leu | CUG | 5.9 | 0.63 | 121 | 0.36 |
| Leu | CUU | 20.1 | 2.15 | 414 | 1.21 |
| Leu | UUA | 34.9 | 3.73 | 719 | 2.09 |
| Leu | UUG | 19.1 | 2.04 | 393 | 1.14 |
| Met | AUG | 99.1 | 2.42 | 466 | 1 |
| Asn | AAC | 21.6 | 0.91 | 175 | 0.43 |
| Asn | AAU | 78.4 | 3.29 | 634 | 1.57 |
| Pro | CCA | 29.7 | 1.25 | 241 | 1.19 |
| Pro | CCC | 17.6 | 0.74 | 143 | 0.51 |
| Pro | CCG | 11.2 | 0.47 | 91 | 0.45 |
| Pro | CCU | 41.5 | 1.75 | 337 | 1.66 |
| Gln | CAA | 79.4 | 2.72 | 525 | 1.59 |
| Gln | CAG | 20.6 | 0.71 | 136 | 0.41 |
| Arg | AGA | 27.9 | 1.68 | 323 | 1.68 |
| Arg | AGG | 8.6 | 0.51 | 99 | 0.51 |
| Arg | CGA | 25.5 | 1.53 | 295 | 1.53 |
| Arg | CGC | 6.1 | 0.36 | 70 | 0.36 |
| Arg | CGG | 6.6 | 0.39 | 76 | 0.39 |
| Arg | CGU | 25.3 | 1.52 | 293 | 1.52 |
| Ser | AGC | 6.8 | 0.47 | 90 | 0.41 |
| Ser | AGU | 20.2 | 1.39 | 268 | 1.21 |
| Ser | UCA | 18.7 | 1.29 | 249 | 1.12 |
| Ser | UCC | 13.9 | 0.96 | 185 | 0.83 |
| Ser | UCG | 7.9 | 0.54 | 105 | 0.47 |
| Ser | UCU | 32.6 | 2.25 | 433 | 1.95 |
| Thr | ACA | 28.8 | 1.53 | 295 | 1.15 |
| Thr | ACC | 19.1 | 1.02 | 196 | 0.77 |
| Thr | ACG | 10.2 | 0.54 | 104 | 0.41 |
| Thr | ACU | 41.9 | 2.23 | 429 | 1.68 |
| Val | GUA | 36.3 | 2.11 | 406 | 1.45 |
| Val | GUC | 13.2 | 0.75 | 145 | 0.7 |
| Val | GUG | 14.1 | 0.81 | 156 | 0.56 |
| Val | GUU | 37 | 2.14 | 413 | 1.48 |
| Trp | UGG | 100 | 1.77 | 342 | 1 |
| Tyr | UAC | 19 | 0.70 | 135 | 0.38 |
| Tyr | UAU | 81 | 2.98 | 575 | 1.62 |
| TER | UAA | 48.8 | 0.20 | 39 | 1.48 |
| TER | UAG | 21.3 | 0.09 | 17 | 0.63 |
| TER | UGA | 30 | 0.12 | 24 | 0.89 |
| Total |  |  | 100.00 | 19278 |  |

**Supplementary Table 10.** Simple sequence repeats in the *Rhododendron × pulchrum* chloroplast genome

| **Sequences** | **Number of repeats** | | | | | | | | | | | |
| --- | --- | --- | --- | --- | --- | --- | --- | --- | --- | --- | --- | --- |
|  | 5 | 6 | 7 | 8 | 9 | 10 | 11 | 12 | 13 | 14 | 15 | total |
| A/T | - | - | - | - | - | 20 | 13 | 4 | 2 | 1 | 1 | 41 |
| AT/AT | - | 3 | 1 | 1 | - | - | - | - | - | - | - | 5 |

**Supplementary Table 11.** Relative synonymous codon usage values of the 5 Ericacea species mitochondrial genomes.

| **MK990822** | | | **MZ779111** | | | **NC_023338** | | | **NC_053763** | | | **OM283814** | | |
| --- | --- | --- | --- | --- | --- | --- | --- | --- | --- | --- | --- | --- | --- | --- |
| **Codon** | **AA** | **RSCU** | **Codon** | **AA** | **RSCU** | **Codon** | **AA** | **RSCU** | **Codon** | **AA** | **RSCU** | **Codon** | **AA** | **RSCU** |
| UAG | Ter | 0.292683 | CAC | His | 0.474227 | CAC | His | 0.446352 | UAG | Ter | 0.365854 | UAG | Ter | 0.333333 |
| CAC | His | 0.439024 | GCG | Ala | 0.474438 | UAC | Tyr | 0.479167 | CAC | His | 0.424028 | CAC | His | 0.435088 |
| UAC | Tyr | 0.504065 | CCG | Pro | 0.502513 | GCG | Ala | 0.502547 | CAG | Gln | 0.504673 | GCG | Ala | 0.492221 |
| GCG | Ala | 0.509299 | UAC | Tyr | 0.535088 | CCG | Pro | 0.515556 | GGC | Gly | 0.506527 | GGC | Gly | 0.506266 |
| CAG | Gln | 0.54878 | GGC | Gly | 0.538318 | CAG | Gln | 0.523438 | GCG | Ala | 0.510145 | ACG | Thr | 0.508711 |
| GGC | Gly | 0.552239 | CGC | Arg | 0.554913 | CUC | Leu | 0.531343 | UAC | Tyr | 0.526596 | UAC | Tyr | 0.514905 |
| CGC | Arg | 0.565891 | CUC | Leu | 0.576112 | GGC | Gly | 0.541732 | AGC | Ser | 0.565619 | CAG | Gln | 0.517007 |
| CCG | Pro | 0.58843 | UAG | Ter | 0.580645 | UAG | Ter | 0.545455 | CGC | Arg | 0.576923 | CCG | Pro | 0.582593 |
| GAC | Asp | 0.59335 | CAG | Gln | 0.606335 | UGA | Ter | 0.545455 | CCG | Pro | 0.579125 | CGC | Arg | 0.593703 |
| GAG | Glu | 0.609848 | ACG | Thr | 0.612346 | ACG | Thr | 0.555102 | ACG | Thr | 0.594595 | CUC | Leu | 0.596302 |
| AGC | Ser | 0.6164 | GAG | Glu | 0.621118 | CGC | Arg | 0.573826 | GAG | Glu | 0.601594 | GAG | Glu | 0.620087 |
| CUC | Leu | 0.62069 | GAC | Asp | 0.623932 | GAC | Asp | 0.597938 | CUC | Leu | 0.616092 | AGC | Ser | 0.642 |
| CUG | Leu | 0.62069 | CUG | Leu | 0.625293 | AGC | Ser | 0.608911 | CUG | Leu | 0.616092 | GAC | Asp | 0.648968 |
| ACG | Thr | 0.636816 | CGG | Arg | 0.682081 | AAC | Asn | 0.625 | GAC | Asp | 0.647727 | CUG | Leu | 0.651772 |
| AAC | Asn | 0.653061 | AGC | Ser | 0.718563 | GAG | Glu | 0.625641 | AAC | Asn | 0.696133 | AAC | Asn | 0.685879 |
| CGG | Arg | 0.689922 | AAC | Asn | 0.718615 | CUG | Leu | 0.650746 | AGG | Arg | 0.733516 | AGG | Arg | 0.710645 |
| AGG | Arg | 0.744186 | AAG | Lys | 0.779141 | CCC | Pro | 0.693333 | CGG | Arg | 0.75 | CGG | Arg | 0.71964 |
| GGG | Gly | 0.756219 | GUC | Val | 0.780684 | CGG | Arg | 0.714765 | CCC | Pro | 0.760943 | GUC | Val | 0.737589 |
| GUC | Val | 0.76217 | CCC | Pro | 0.79397 | GGG | Gly | 0.724409 | GUC | Val | 0.761905 | UGC | Cys | 0.785276 |
| AAG | Lys | 0.781132 | AUA | Ile | 0.813913 | GUC | Val | 0.773973 | GGG | Gly | 0.778068 | CCC | Pro | 0.788632 |
| CCC | Pro | 0.793388 | AUC | Ile | 0.824348 | AAG | Lys | 0.788043 | AAG | Lys | 0.798165 | AAG | Lys | 0.789598 |
| AUC | Ile | 0.822148 | UGC | Cys | 0.827586 | AUA | Ile | 0.795918 | UGA | Ter | 0.804878 | UCG | Ser | 0.804 |
| UCG | Ser | 0.825636 | GGG | Gly | 0.829907 | AGG | Arg | 0.825503 | UGC | Cys | 0.818182 | GGG | Gly | 0.822055 |
| UUC | Phe | 0.837629 | GUG | Val | 0.84507 | UGC | Cys | 0.827586 | AUA | Ile | 0.828283 | AUA | Ile | 0.841629 |
| UGC | Cys | 0.84153 | UUC | Phe | 0.852525 | GUG | Val | 0.849315 | UCG | Ser | 0.831793 | UUC | Phe | 0.860728 |
| AUA | Ile | 0.865772 | UCG | Ser | 0.862275 | AUC | Ile | 0.857143 | UUC | Phe | 0.869231 | AUC | Ile | 0.882353 |
| CUA | Leu | 0.905747 | UGA | Ter | 0.870968 | UCG | Ser | 0.868812 | GUG | Val | 0.879552 | CUA | Leu | 0.892142 |
| GUG | Val | 0.906815 | AGG | Arg | 0.878613 | UUC | Phe | 0.870968 | CUA | Leu | 0.882759 | GUG | Val | 0.924823 |
| GCC | Ala | 0.944206 | CUA | Leu | 0.920375 | CUA | Leu | 0.931343 | AUC | Ile | 0.885522 | GCC | Ala | 0.96181 |
| ACA | Thr | 0.981758 | GCC | Ala | 0.940695 | ACA | Thr | 0.946939 | GCC | Ala | 0.898551 | ACA | Thr | 0.996516 |
| UCC | Ser | 0.983977 | UCC | Ser | 0.961078 | GCC | Ala | 0.964346 | UGG | Trp | 1 | UGG | Trp | 1 |
| UGG | Trp | 1 | UGG | Trp | 1 | UCC | Ser | 0.99505 | AUG | Met | 1 | AUG | Met | 1 |
| AUG | Met | 1 | AUG | Met | 1 | UGG | Trp | 1 | ACA | Thr | 1.01351 | UGA | Ter | 1 |
| GCA | Ala | 1.01288 | ACC | Thr | 1.00741 | AUG | Met | 1 | GCA | Ala | 1.03188 | GCA | Ala | 1.0297 |
| UGA | Ter | 1.02439 | GCA | Ala | 1.01431 | UCA | Ser | 1.02475 | UCC | Ser | 1.03697 | UCC | Ser | 1.032 |
| ACC | Thr | 1.06136 | ACA | Thr | 1.02716 | GCA | Ala | 1.02547 | ACC | Thr | 1.06757 | UCA | Ser | 1.068 |
| UCA | Ser | 1.06315 | UCA | Ser | 1.03293 | ACC | Thr | 1.05306 | UCA | Ser | 1.08133 | ACC | Thr | 1.07317 |
| CCA | Pro | 1.07107 | AGU | Ser | 1.07784 | CGU | Arg | 1.09732 | GUA | Val | 1.09244 | GUA | Val | 1.07801 |
| AGU | Ser | 1.07446 | CGU | Arg | 1.09827 | AGU | Ser | 1.12129 | AGU | Ser | 1.1146 | UUG | Leu | 1.11864 |
| GUA | Val | 1.12378 | UUU | Phe | 1.14747 | UUU | Phe | 1.12903 | UUU | Phe | 1.13077 | CCA | Pro | 1.13677 |
| CGU | Arg | 1.15504 | UUG | Leu | 1.15222 | GUA | Val | 1.13014 | UGU | Cys | 1.18182 | UUU | Phe | 1.13927 |
| UGU | Cys | 1.15847 | CCA | Pro | 1.15578 | UUG | Leu | 1.15821 | CCA | Pro | 1.18519 | AGU | Ser | 1.14 |
| UUU | Phe | 1.16237 | GGU | Gly | 1.16636 | UGU | Cys | 1.17241 | AAA | Lys | 1.20183 | CGU | Arg | 1.16042 |
| GUU | Val | 1.20723 | UGU | Cys | 1.17241 | CCA | Pro | 1.18222 | UUG | Leu | 1.2046 | AAA | Lys | 1.2104 |
| AAA | Lys | 1.21887 | GUA | Val | 1.17505 | AAA | Lys | 1.21196 | CGU | Arg | 1.22802 | UGU | Cys | 1.21472 |
| UUG | Leu | 1.22299 | GUU | Val | 1.1992 | CUU | Leu | 1.23582 | CUU | Leu | 1.24138 | GGU | Gly | 1.25815 |
| CUU | Leu | 1.24598 | AAA | Lys | 1.22086 | GUU | Val | 1.24658 | GUU | Val | 1.26611 | GUU | Val | 1.25957 |
| GGU | Gly | 1.24876 | CUU | Leu | 1.25059 | GGU | Gly | 1.27244 | AUU | Ile | 1.2862 | CUU | Leu | 1.26656 |
| AUU | Ile | 1.31208 | AAU | Asn | 1.28139 | AUU | Ile | 1.34694 | GGU | Gly | 1.28982 | AUU | Ile | 1.27602 |
| ACU | Thr | 1.32007 | CGA | Arg | 1.28324 | GAA | Glu | 1.37436 | AAU | Asn | 1.30387 | UCU | Ser | 1.314 |
| AAU | Asn | 1.34694 | UCU | Ser | 1.34731 | AAU | Asn | 1.375 | CGA | Arg | 1.31868 | AAU | Asn | 1.31412 |
| CGA | Arg | 1.37209 | ACU | Thr | 1.35309 | UCU | Ser | 1.38119 | ACU | Thr | 1.32432 | CGA | Arg | 1.34033 |
| UUA | Leu | 1.38391 | AUU | Ile | 1.36174 | AGA | Arg | 1.38926 | GAU | Asp | 1.35227 | GAU | Asp | 1.35103 |
| GAA | Glu | 1.39015 | GAU | Asp | 1.37607 | CGA | Arg | 1.39933 | UCU | Ser | 1.36969 | GAA | Glu | 1.37991 |
| GAU | Asp | 1.40665 | GAA | Glu | 1.37888 | GAU | Asp | 1.40206 | AGA | Arg | 1.39286 | GGA | Gly | 1.41353 |
| UCU | Ser | 1.43638 | CAA | Gln | 1.39367 | ACU | Thr | 1.4449 | GAA | Glu | 1.39841 | ACU | Thr | 1.4216 |
| GGA | Gly | 1.44279 | UAU | Tyr | 1.46491 | GGA | Gly | 1.46142 | GGA | Gly | 1.42559 | UUA | Leu | 1.47458 |
| CAA | Gln | 1.45122 | GGA | Gly | 1.46542 | CAA | Gln | 1.47656 | UUA | Leu | 1.43908 | AGA | Arg | 1.47526 |
| AGA | Arg | 1.47287 | UUA | Leu | 1.47541 | UUA | Leu | 1.49254 | UAU | Tyr | 1.4734 | CAA | Gln | 1.48299 |
| UAU | Tyr | 1.49593 | AGA | Arg | 1.50289 | GCU | Ala | 1.50764 | CCU | Pro | 1.47475 | UAU | Tyr | 1.48509 |
| GCU | Ala | 1.53362 | CAU | His | 1.52577 | UAU | Tyr | 1.52083 | CAA | Gln | 1.49533 | CCU | Pro | 1.49201 |
| CCU | Pro | 1.54711 | CCU | Pro | 1.54774 | CAU | His | 1.55365 | GCU | Ala | 1.55942 | GCU | Ala | 1.51627 |
| CAU | His | 1.56098 | UAA | Ter | 1.54839 | CCU | Pro | 1.60889 | CAU | His | 1.57597 | CAU | His | 1.56491 |
| UAA | Ter | 1.68293 | GCU | Ala | 1.57055 | UAA | Ter | 1.90909 | UAA | Ter | 1.82927 | UAA | Ter | 1.66667 |

**Supplementary Table 12.** Relative synonymous codon usage values of the 15 *Rhododendron* species chloroplast genomes.

| **MN182619.2** | | | **MN711645** | | | **MT239363** | | | **MT239364** | | | **MT239365** | | | **MT239366** | | | **MT985162** | | | **MZ073672** | | |
| --- | --- | --- | --- | --- | --- | --- | --- | --- | --- | --- | --- | --- | --- | --- | --- | --- | --- | --- | --- | --- | --- | --- | --- |
|  | | |  | | |  | | |  | | |  | | |  | | |  | | |  | | |
| Codon | AA | RSCU | Codon | AA | RSCU | Codon | AA | RSCU | Codon | AA | RSCU | Codon | AA | RSCU | Codon | AA | RSCU | Codon | AA | RSCU | Codon | AA | RSCU |
| UUA | Leu | 2.09621 | UUA | Leu | 2.17197 | UUA | Leu | 2.16264 | UUA | Leu | 2.08086 | UUA | Leu | 2.17304 | UUA | Leu | 2.15129 | UUA | Leu | 2.11502 | UUA | Leu | 2.15265 |
| UCU | Ser | 1.95338 | UCU | Ser | 1.9221 | UCU | Ser | 1.93588 | UCU | Ser | 1.91785 | UCU | Ser | 1.94758 | UCU | Ser | 1.95968 | UCU | Ser | 1.91226 | UCU | Ser | 1.96709 |
| GCU | Ala | 1.81757 | GCU | Ala | 1.82595 | GCU | Ala | 1.82126 | GCU | Ala | 1.82064 | GCU | Ala | 1.81818 | GCU | Ala | 1.81231 | GCU | Ala | 1.82007 | GCU | Ala | 1.81684 |
| ACU | Thr | 1.67742 | ACU | Thr | 1.67906 | AGA | Arg | 1.66749 | AGA | Arg | 1.73591 | AGA | Arg | 1.66341 | ACU | Thr | 1.65792 | ACU | Thr | 1.68621 | CCU | Pro | 1.67853 |
| AGA | Arg | 1.67647 | AGA | Arg | 1.655 | ACU | Thr | 1.65872 | ACU | Thr | 1.6333 | ACU | Thr | 1.66099 | AGA | Arg | 1.64803 | AGA | Arg | 1.66017 | ACU | Thr | 1.67619 |
| CCU | Pro | 1.6601 | CCU | Pro | 1.65072 | CCU | Pro | 1.65345 | GAU | Asp | 1.62742 | GAU | Asp | 1.62551 | CCU | Pro | 1.63942 | CCU | Pro | 1.63927 | AGA | Arg | 1.64501 |
| UAU | Tyr | 1.61972 | GAU | Asp | 1.61265 | GAU | Asp | 1.62036 | CCU | Pro | 1.62044 | CCU | Pro | 1.625 | UAU | Tyr | 1.61965 | GAU | Asp | 1.60745 | UAU | Tyr | 1.62559 |
| GAU | Asp | 1.6101 | UAU | Tyr | 1.60692 | UAU | Tyr | 1.60453 | UAU | Tyr | 1.60857 | UAU | Tyr | 1.61809 | GAU | Asp | 1.6158 | UAU | Tyr | 1.59527 | GAU | Asp | 1.61187 |
| UGU | Cys | 1.5907 | CAA | Gln | 1.6 | CAA | Gln | 1.59775 | CAA | Gln | 1.57407 | CAA | Gln | 1.59944 | CAA | Gln | 1.60508 | CAA | Gln | 1.59205 | CAA | Gln | 1.59249 |
| CAA | Gln | 1.5885 | AAU | Asn | 1.58192 | CGU | Arg | 1.57816 | UGU | Cys | 1.56481 | CGU | Arg | 1.58049 | CGU | Arg | 1.58882 | CGU | Arg | 1.59002 | AAU | Asn | 1.59081 |
| AAU | Asn | 1.56737 | CGU | Arg | 1.57689 | AAU | Asn | 1.56863 | GGA | Gly | 1.54286 | UGU | Cys | 1.57025 | GGA | Gly | 1.56399 | AAU | Asn | 1.5818 | CGU | Arg | 1.5832 |
| AAA | Lys | 1.54246 | UGU | Cys | 1.56627 | UGU | Cys | 1.56846 | AAU | Asn | 1.53941 | AAA | Lys | 1.56741 | AAU | Asn | 1.56293 | UGU | Cys | 1.57312 | UGU | Cys | 1.57031 |
| GGA | Gly | 1.53715 | AAA | Lys | 1.56012 | AAA | Lys | 1.5573 | CAU | His | 1.51927 | AAU | Asn | 1.56651 | CGA | Arg | 1.55921 | GGA | Gly | 1.54863 | GUU | Val | 1.56815 |
| CGA | Arg | 1.53114 | GGA | Gly | 1.5465 | CAU | His | 1.55414 | GAA | Glu | 1.5115 | GGA | Gly | 1.56343 | AAA | Lys | 1.55577 | GUU | Val | 1.54462 | GGA | Gly | 1.56554 |
| CAU | His | 1.52968 | GUU | Val | 1.54403 | GGA | Gly | 1.55398 | AUU | Ile | 1.49823 | CGA | Arg | 1.56098 | CAU | His | 1.54797 | AAA | Lys | 1.54116 | AAA | Lys | 1.54579 |
| GAA | Glu | 1.524 | CAU | His | 1.54176 | CGA | Arg | 1.54839 | AAA | Lys | 1.49522 | CAU | His | 1.54797 | UGU | Cys | 1.54167 | GAA | Glu | 1.5315 | CGA | Arg | 1.54041 |
| CGU | Arg | 1.52076 | GAA | Glu | 1.53876 | GAA | Glu | 1.54291 | CGA | Arg | 1.48865 | GAA | Glu | 1.54635 | GAA | Glu | 1.53877 | CAU | His | 1.52795 | CAU | His | 1.5375 |
| AUU | Ile | 1.50897 | CGA | Arg | 1.53295 | GUU | Val | 1.54117 | GUU | Val | 1.46867 | GUU | Val | 1.5309 | GUU | Val | 1.53773 | CGA | Arg | 1.5152 | GAA | Glu | 1.53382 |
| UAA | Ter | 1.48148 | AUU | Ile | 1.53109 | AUU | Ile | 1.51101 | GUA | Val | 1.45102 | AUU | Ile | 1.52426 | AUU | Ile | 1.51485 | AUU | Ile | 1.50379 | AUU | Ile | 1.51192 |
| GUU | Val | 1.47896 | GUA | Val | 1.41509 | GUA | Val | 1.43565 | CGU | Arg | 1.44828 | GUA | Val | 1.43582 | GUA | Val | 1.43606 | GUA | Val | 1.42769 | GUA | Val | 1.39663 |
| GUA | Val | 1.45389 | UUU | Phe | 1.38298 | UUU | Phe | 1.39203 | UUU | Phe | 1.36725 | UAA | Ter | 1.4 | UUU | Phe | 1.37767 | UUU | Phe | 1.38737 | UUU | Phe | 1.38816 |
| UUU | Phe | 1.37581 | GGU | Gly | 1.35287 | UAA | Ter | 1.34483 | GGU | Gly | 1.35429 | UUU | Phe | 1.3792 | UAA | Ter | 1.35165 | UAA | Ter | 1.35484 | GGU | Gly | 1.33908 |
| GGU | Gly | 1.37013 | UAA | Ter | 1.32955 | GGU | Gly | 1.34454 | UAA | Ter | 1.23077 | GGU | Gly | 1.33677 | GGU | Gly | 1.33762 | GGU | Gly | 1.33915 | UAA | Ter | 1.28571 |
| AGU | Ser | 1.20902 | AGU | Ser | 1.23707 | CUU | Leu | 1.23429 | CUU | Leu | 1.21578 | CUU | Leu | 1.2287 | AGU | Ser | 1.21774 | AGU | Ser | 1.22323 | CUU | Leu | 1.25388 |
| CUU | Leu | 1.207 | CUU | Leu | 1.22012 | AGU | Ser | 1.22374 | CCA | Pro | 1.19708 | ACA | Thr | 1.21614 | CUU | Leu | 1.21469 | CUU | Leu | 1.22135 | ACA | Thr | 1.21905 |
| CCA | Pro | 1.18719 | CCA | Pro | 1.18799 | ACA | Thr | 1.20367 | AGU | Ser | 1.18745 | AGU | Ser | 1.20968 | ACA | Thr | 1.21267 | CCA | Pro | 1.21238 | AGU | Ser | 1.21519 |
| ACA | Thr | 1.15347 | ACA | Thr | 1.18586 | CCA | Pro | 1.18686 | ACA | Thr | 1.16551 | CCA | Pro | 1.18304 | CCA | Pro | 1.18701 | ACA | Thr | 1.17241 | CCA | Pro | 1.19525 |
| UUG | Leu | 1.14577 | UCA | Ser | 1.1323 | UCA | Ser | 1.1337 | UUG | Leu | 1.15149 | UCA | Ser | 1.15323 | UUG | Leu | 1.14648 | UCA | Ser | 1.14968 | UCA | Ser | 1.15443 |
| UCA | Ser | 1.12331 | UUG | Leu | 1.12984 | UUG | Leu | 1.1156 | UCA | Ser | 1.11576 | UUG | Leu | 1.13478 | UCA | Ser | 1.14516 | UUG | Leu | 1.11709 | UUG | Leu | 1.12163 |
| GCA | Ala | 1.08787 | GCA | Ala | 1.08092 | GCA | Ala | 1.10015 | GCA | Ala | 1.08968 | GCA | Ala | 1.1123 | GCA | Ala | 1.10769 | GCA | Ala | 1.09442 | GCA | Ala | 1.06942 |
| UGG | Trp | 1 | UGG | Trp | 1 | UGG | Trp | 1 | UGG | Trp | 1 | UGA | Ter | 1.03333 | UGG | Trp | 1 | UGG | Trp | 1 | UGA | Ter | 1.05495 |
| AUG | Met | 1 | AUG | Met | 1 | AUG | Met | 1 | AUG | Met | 1 | UGG | Trp | 1 | AUG | Met | 1 | AUG | Met | 1 | UGG | Trp | 1 |
| AUA | Ile | 0.91866 | UGA | Ter | 0.988636 | UGA | Ter | 1 | UAG | Ter | 0.974359 | AUG | Met | 1 | UGA | Ter | 0.989011 | UGA | Ter | 0.967742 | AUG | Met | 1 |
| UGA | Ter | 0.888889 | AUA | Ile | 0.903109 | AUA | Ile | 0.918239 | AUA | Ile | 0.902123 | AUA | Ile | 0.909233 | AUA | Ile | 0.917665 | AUA | Ile | 0.91713 | AUA | Ile | 0.913108 |
| UCC | Ser | 0.834586 | UCC | Ser | 0.854265 | UCC | Ser | 0.855389 | UCC | Ser | 0.864824 | UCC | Ser | 0.862903 | UCC | Ser | 0.850806 | UCC | Ser | 0.843871 | UCC | Ser | 0.820253 |
| CUA | Leu | 0.804665 | CUA | Leu | 0.789338 | CUA | Leu | 0.804396 | CUA | Leu | 0.821237 | CUA | Leu | 0.777391 | CUA | Leu | 0.789681 | CUA | Leu | 0.80182 | CUA | Leu | 0.788571 |
| ACC | Thr | 0.766373 | ACC | Thr | 0.761559 | ACC | Thr | 0.759633 | UGA | Ter | 0.794872 | ACC | Thr | 0.749776 | ACC | Thr | 0.756561 | ACC | Thr | 0.748276 | ACC | Thr | 0.734199 |
| CCC | Pro | 0.704433 | CCC | Pro | 0.694105 | CCC | Pro | 0.693092 | ACC | Thr | 0.780971 | CCC | Pro | 0.700893 | CCC | Pro | 0.68981 | CCC | Pro | 0.695838 | GGG | Gly | 0.669538 |
| GGG | Gly | 0.668082 | UAG | Ter | 0.681818 | GGG | Gly | 0.664512 | CCC | Pro | 0.690998 | GGG | Gly | 0.66452 | GGG | Gly | 0.661093 | GGG | Gly | 0.680798 | CCC | Pro | 0.660194 |
| UAG | Ter | 0.62963 | GGG | Gly | 0.667516 | UAG | Ter | 0.655172 | GGG | Gly | 0.677143 | UUC | Phe | 0.620803 | UAG | Ter | 0.659341 | UAG | Ter | 0.677419 | UAG | Ter | 0.659341 |
| GCC | Ala | 0.629289 | GCC | Ala | 0.638168 | GCC | Ala | 0.619414 | UUC | Phe | 0.632747 | GCC | Ala | 0.620321 | GCC | Ala | 0.627692 | GCC | Ala | 0.621561 | GCC | Ala | 0.646972 |
| UUC | Phe | 0.624192 | UUC | Phe | 0.617021 | UUC | Phe | 0.607973 | GCC | Ala | 0.615905 | UAG | Ter | 0.566667 | UUC | Phe | 0.622332 | UUC | Phe | 0.612627 | UUC | Phe | 0.611837 |
| AUC | Ile | 0.572368 | AUC | Ile | 0.565803 | AUC | Ile | 0.570755 | AUC | Ile | 0.599646 | AUC | Ile | 0.56651 | AUC | Ile | 0.567483 | AUC | Ile | 0.57908 | AUC | Ile | 0.574975 |
| GUG | Val | 0.555058 | GUG | Val | 0.525157 | GUG | Val | 0.514788 | GUG | Val | 0.575463 | GUG | Val | 0.51981 | GUG | Val | 0.517871 | GUC | Val | 0.513846 | GUC | Val | 0.526799 |
| AGG | Arg | 0.513841 | GUC | Val | 0.515723 | GUC | Val | 0.508393 | AGG | Arg | 0.560135 | GUC | Val | 0.513471 | GUC | Val | 0.50834 | GUG | Val | 0.513846 | GUG | Val | 0.508423 |
| GUC | Val | 0.512086 | AGG | Arg | 0.478438 | UCG | Ser | 0.478854 | UCG | Ser | 0.524272 | CCG | Pro | 0.491071 | CCG | Pro | 0.483763 | AGG | Arg | 0.509743 | UCG | Ser | 0.478481 |
| GAG | Glu | 0.475996 | UCG | Ser | 0.475487 | CCG | Pro | 0.466591 | GUC | Val | 0.504854 | UCG | Ser | 0.459677 | AGG | Arg | 0.463816 | UCG | Ser | 0.483871 | AGG | Arg | 0.475436 |
| UCG | Ser | 0.473684 | CCG | Pro | 0.467186 | GCG | Ala | 0.459168 | AAG | Lys | 0.50478 | AGG | Arg | 0.458537 | GAG | Glu | 0.461233 | CAC | His | 0.47205 | GCG | Ala | 0.466765 |
| CAC | His | 0.47032 | GAG | Glu | 0.46124 | GAG | Glu | 0.457086 | CCG | Pro | 0.491484 | GAG | Glu | 0.453649 | UGC | Cys | 0.458333 | GAG | Glu | 0.4685 | GAG | Glu | 0.466179 |
| GCG | Ala | 0.465272 | CAC | His | 0.458244 | AGG | Arg | 0.456576 | GAG | Glu | 0.488499 | CAC | His | 0.452026 | UCG | Ser | 0.455645 | GCG | Ala | 0.463941 | CCG | Pro | 0.466019 |
| AAG | Lys | 0.457542 | GCG | Ala | 0.454962 | CAC | His | 0.44586 | CAC | His | 0.480726 | GCG | Ala | 0.449198 | GCG | Ala | 0.452308 | AAG | Lys | 0.458844 | CAC | His | 0.4625 |
| CCG | Pro | 0.448276 | AAG | Lys | 0.439883 | AAG | Lys | 0.442703 | GCG | Ala | 0.473773 | GGC | Gly | 0.435287 | CAC | His | 0.452026 | CCG | Pro | 0.452508 | AAG | Lys | 0.454212 |
| AAC | Asn | 0.432633 | UGC | Cys | 0.433735 | GGC | Gly | 0.436975 | AAC | Asn | 0.460591 | AAC | Asn | 0.433486 | AAG | Lys | 0.444229 | GGC | Gly | 0.431421 | UGC | Cys | 0.429688 |
| GGC | Gly | 0.424628 | GGC | Gly | 0.433121 | UGC | Cys | 0.431535 | UGC | Cys | 0.435185 | AAG | Lys | 0.43259 | GGC | Gly | 0.437299 | UGC | Cys | 0.426877 | GGC | Gly | 0.425846 |
| CAG | Gln | 0.411498 | AAC | Asn | 0.418079 | AAC | Asn | 0.431373 | CAG | Gln | 0.425926 | UGC | Cys | 0.429752 | AAC | Asn | 0.437071 | AAC | Asn | 0.418202 | AAC | Asn | 0.40919 |
| UGC | Cys | 0.409302 | CGG | Arg | 0.400325 | CAG | Gln | 0.40225 | GGC | Gly | 0.425714 | CAG | Gln | 0.400556 | CAG | Gln | 0.394922 | CAG | Gln | 0.407947 | CAG | Gln | 0.407507 |
| AGC | Ser | 0.406015 | CAG | Gln | 0.4 | CGG | Arg | 0.397022 | ACG | Thr | 0.420218 | CGG | Arg | 0.390244 | CGG | Arg | 0.394737 | UAC | Tyr | 0.404734 | CGG | Arg | 0.40412 |
| ACG | Thr | 0.402737 | UAC | Tyr | 0.393078 | UAC | Tyr | 0.395466 | CGG | Arg | 0.413793 | UAC | Tyr | 0.38191 | GAC | Asp | 0.384196 | ACG | Thr | 0.393103 | GAC | Asp | 0.388133 |
| CGG | Arg | 0.394464 | GAC | Asp | 0.387352 | GAC | Asp | 0.379642 | UAC | Tyr | 0.391429 | GAC | Asp | 0.374491 | UAC | Tyr | 0.380353 | GAC | Asp | 0.392547 | UAC | Tyr | 0.374408 |
| CUC | Leu | 0.393586 | AGC | Ser | 0.378778 | ACG | Thr | 0.377982 | AGC | Ser | 0.389843 | ACG | Thr | 0.373094 | ACG | Thr | 0.372851 | CGG | Arg | 0.388153 | ACG | Thr | 0.370563 |
| GAC | Asp | 0.389902 | ACG | Thr | 0.373527 | AGC | Ser | 0.372442 | GAC | Asp | 0.372578 | AGC | Ser | 0.366935 | AGC | Ser | 0.370968 | AGC | Ser | 0.387097 | AGC | Ser | 0.364557 |
| UAC | Tyr | 0.380282 | CGC | Arg | 0.356387 | CGC | Arg | 0.352357 | CUC | Leu | 0.368242 | CUC | Leu | 0.352174 | CUC | Leu | 0.354176 | CUC | Leu | 0.37981 | CUC | Leu | 0.357551 |
| CGC | Arg | 0.363322 | CUC | Leu | 0.353396 | CUC | Leu | 0.348132 | CUG | Leu | 0.362396 | CGC | Arg | 0.346341 | CGC | Arg | 0.345395 | CUG | Leu | 0.364915 | CGC | Arg | 0.351823 |
| CUG | Leu | 0.35277 | CUG | Leu | 0.33534 | CUG | Leu | 0.334945 | CGC | Arg | 0.353238 | CUG | Leu | 0.333913 | CUG | Leu | 0.343682 | CGC | Arg | 0.336711 | CUG | Leu | 0.325714 |

| **NC_047438** | | | **NC_050162** | | | **NC_057644** | | | **NC_058233** | | | **DRR298903-DRR298907** | | | **SRR12917131-SRR12917132** | | | **SRR13425299** | | |
| --- | --- | --- | --- | --- | --- | --- | --- | --- | --- | --- | --- | --- | --- | --- | --- | --- | --- | --- | --- | --- |
| Codon | AA | RSCU | Codon | AA | RSCU | Codon | AA | RSCU | Codon | AA | RSCU | Codon | AA | RSCU | Codon | AA | RSCU | Codon | AA | RSCU |
| UUA | Leu | 2.13218 | UUA | Leu | 2.12831 | UUA | Leu | 2.1134 | UUA | Leu | 2.112 | GCA | Ala | 1.08326 | GCA | Ala | 1.12256 | GCA | Ala | 1.10083 |
| UCU | Ser | 2.02257 | UCU | Ser | 1.90488 | UCU | Ser | 1.92807 | UCU | Ser | 1.92471 | GCC | Ala | 0.632464 | GCC | Ala | 0.623955 | GCC | Ala | 0.624793 |
| GCU | Ala | 1.81848 | GCU | Ala | 1.80236 | GCU | Ala | 1.8312 | GCU | Ala | 1.82935 | GCG | Ala | 0.464256 | GCG | Ala | 0.470752 | GCG | Ala | 0.46281 |
| CCU | Pro | 1.67895 | AGA | Arg | 1.66977 | CCU | Pro | 1.67134 | AGA | Arg | 1.6688 | GCT | Ala | 1.82002 | GCT | Ala | 1.78273 | GCT | Ala | 1.81157 |
| ACU | Thr | 1.64729 | ACU | Thr | 1.66957 | ACU | Thr | 1.66565 | CCU | Pro | 1.66837 | AGA | Arg | 1.6687 | AGA | Arg | 1.65275 | AGA | Arg | 1.70472 |
| AGA | Arg | 1.63395 | CCU | Pro | 1.65032 | AGA | Arg | 1.66272 | ACU | Thr | 1.6626 | AGG | Arg | 0.528335 | AGG | Arg | 0.523077 | AGG | Arg | 0.515021 |
| GAU | Asp | 1.61834 | GAU | Asp | 1.60939 | UAU | Tyr | 1.62899 | UAU | Tyr | 1.62717 | CGA | Arg | 1.52223 | CGA | Arg | 1.55604 | CGA | Arg | 1.52446 |
| UAU | Tyr | 1.60504 | UAU | Tyr | 1.60191 | GAU | Asp | 1.59825 | GAU | Asp | 1.59649 | CGC | Arg | 0.366173 | CGC | Arg | 0.325275 | CGC | Arg | 0.350215 |
| CGU | Arg | 1.59682 | CAA | Gln | 1.5853 | CAA | Gln | 1.57795 | CAA | Gln | 1.57746 | CGG | Arg | 0.40279 | CGG | Arg | 0.417582 | CGG | Arg | 0.381116 |
| CAA | Gln | 1.586 | CGU | Arg | 1.5814 | CGU | Arg | 1.56974 | CGU | Arg | 1.56999 | CGT | Arg | 1.51177 | CGT | Arg | 1.52527 | CGT | Arg | 1.52446 |
| UGU | Cys | 1.58182 | AAU | Asn | 1.57576 | AAU | Asn | 1.56298 | AAA | Lys | 1.56263 | AAC | Asn | 0.432964 | AAC | Asn | 0.423077 | AAC | Asn | 0.442822 |
| AAA | Lys | 1.57098 | AAA | Lys | 1.55275 | AAA | Lys | 1.56211 | AAU | Asn | 1.56242 | AAT | Asn | 1.56704 | AAT | Asn | 1.57692 | AAT | Asn | 1.55718 |
| AAU | Asn | 1.55919 | GGA | Gly | 1.54512 | UGU | Cys | 1.5534 | UGU | Cys | 1.5534 | GAC | Asp | 0.391608 | GAC | Asp | 0.390129 | GAC | Asp | 0.406685 |
| GUU | Val | 1.55633 | UGU | Cys | 1.54086 | GGA | Gly | 1.54624 | GGA | Gly | 1.54848 | GAT | Asp | 1.60839 | GAT | Asp | 1.60987 | GAT | Asp | 1.59331 |
| GAA | Glu | 1.54873 | GAA | Glu | 1.54081 | CGA | Arg | 1.54239 | CAU | His | 1.54177 | TGC | Cys | 0.412844 | TGC | Cys | 0.435424 | TGC | Cys | 0.464912 |
| GGA | Gly | 1.54633 | GUU | Val | 1.5394 | CAU | His | 1.54137 | CGA | Arg | 1.53705 | TGT | Cys | 1.58716 | TGT | Cys | 1.56458 | TGT | Cys | 1.53509 |
| CGA | Arg | 1.53846 | CAU | His | 1.53753 | GAA | Glu | 1.53552 | GAA | Glu | 1.5334 | CAA | Gln | 1.58422 | CAA | Gln | 1.58816 | CAA | Gln | 1.58383 |
| CAU | His | 1.53776 | AUU | Ile | 1.51431 | AUU | Ile | 1.51936 | AUU | Ile | 1.51665 | CAG | Gln | 0.415781 | CAG | Gln | 0.411837 | CAG | Gln | 0.416168 |
| AUU | Ile | 1.52009 | CGA | Arg | 1.50698 | GUU | Val | 1.49451 | GUU | Val | 1.49817 | GAA | Glu | 1.51943 | GAA | Glu | 1.52801 | GAA | Glu | 1.52823 |
| UAA | Ter | 1.425 | GUA | Val | 1.42311 | GUA | Val | 1.44689 | GUA | Val | 1.44322 | GAG | Glu | 0.480573 | GAG | Glu | 0.471986 | GAG | Glu | 0.471774 |
| GUA | Val | 1.42461 | UUU | Phe | 1.37764 | UAA | Ter | 1.4026 | UAA | Ter | 1.36364 | GGA | Gly | 1.53737 | GGA | Gly | 1.56655 | GGA | Gly | 1.52502 |
| UUU | Phe | 1.3835 | GGU | Gly | 1.34981 | GGU | Gly | 1.35838 | GGU | Gly | 1.35745 | GGC | Gly | 0.427046 | GGC | Gly | 0.424028 | GGC | Gly | 0.43129 |
| GGU | Gly | 1.37206 | UAA | Ter | 1.32632 | UUU | Phe | 1.35831 | UUU | Phe | 1.35441 | GGG | Gly | 0.669039 | GGG | Gly | 0.69258 | GGG | Gly | 0.682171 |
| ACA | Thr | 1.23046 | CUU | Leu | 1.21689 | AGU | Ser | 1.22909 | AGU | Ser | 1.23294 | GGT | Gly | 1.36655 | GGT | Gly | 1.31684 | GGT | Gly | 1.36152 |
| CUU | Leu | 1.22701 | AGU | Ser | 1.19923 | CUU | Leu | 1.21626 | CUU | Leu | 1.218 | CAC | His | 0.47032 | CAC | His | 0.469667 | CAC | His | 0.467706 |
| AGU | Ser | 1.2009 | ACA | Thr | 1.19652 | ACA | Thr | 1.17933 | ACA | Thr | 1.1748 | CAT | His | 1.52968 | CAT | His | 1.53033 | CAT | His | 1.53229 |
| CCA | Pro | 1.16528 | CCA | Pro | 1.18977 | CCA | Pro | 1.15669 | CCA | Pro | 1.15816 | ATA | Ile | 0.910072 | ATA | Ile | 0.922751 | ATA | Ile | 0.907683 |
| UUG | Leu | 1.15517 | UCA | Ser | 1.14524 | UUG | Leu | 1.14701 | UUG | Leu | 1.146 | ATC | Ile | 0.571942 | ATC | Ile | 0.583608 | ATC | Ile | 0.578916 |
| GCA | Ala | 1.10561 | GCA | Ala | 1.10914 | UCA | Ser | 1.14464 | UCA | Ser | 1.14353 | ATT | Ile | 1.51799 | ATT | Ile | 1.49364 | ATT | Ile | 1.5134 |
| UCA | Ser | 1.08804 | UUG | Leu | 1.09768 | GCA | Ala | 1.10486 | GCA | Ala | 1.10239 | CTA | Leu | 0.801737 | CTA | Leu | 0.787077 | CTA | Leu | 0.800578 |
| UGG | Trp | 1 | UGG | Trp | 1 | AUG | Met | 1 | AUG | Met | 1 | CTC | Leu | 0.396527 | CTC | Leu | 0.376022 | CTC | Leu | 0.381503 |
| AUG | Met | 1 | AUG | Met | 1 | UGG | Trp | 1 | UGG | Trp | 1 | CTG | Leu | 0.356006 | CTG | Leu | 0.373686 | CTG | Leu | 0.358382 |
| UGA | Ter | 0.975 | UGA | Ter | 0.947368 | AUA | Ile | 0.905347 | UGA | Ter | 0.935065 | CTT | Leu | 1.19537 | CTT | Leu | 1.23083 | CTT | Leu | 1.20809 |
| AUA | Ile | 0.901573 | AUA | Ile | 0.913109 | UGA | Ter | 0.896104 | AUA | Ile | 0.906289 | TTA | Leu | 2.1042 | TTA | Leu | 2.13235 | TTA | Leu | 2.12428 |
| UCC | Ser | 0.862302 | UCC | Ser | 0.859897 | UCC | Ser | 0.839719 | UCC | Ser | 0.837647 | TTG | Leu | 1.14616 | TTG | Leu | 1.10004 | TTG | Leu | 1.12717 |
| CUA | Leu | 0.793103 | CUA | Leu | 0.812086 | CUA | Leu | 0.815855 | CUA | Leu | 0.813 | AAA | Lys | 1.54062 | AAA | Lys | 1.53557 | AAA | Lys | 1.5443 |
| ACC | Thr | 0.741483 | ACC | Thr | 0.751304 | ACC | Thr | 0.786221 | ACC | Thr | 0.792683 | AAG | Lys | 0.459378 | AAG | Lys | 0.464432 | AAG | Lys | 0.455696 |
| CCC | Pro | 0.694411 | UAG | Ter | 0.726316 | CCC | Pro | 0.718471 | CCC | Pro | 0.714286 | ATG | Met | 1 | ATG | Met | 1 | ATG | Met | 1 |
| GGG | Gly | 0.639004 | CCC | Pro | 0.695096 | UAG | Ter | 0.701299 | UAG | Ter | 0.701299 | TTC | Phe | 0.626263 | TTC | Phe | 0.618364 | TTC | Phe | 0.635531 |
| GCC | Ala | 0.623762 | GGG | Gly | 0.677379 | GGG | Gly | 0.653179 | GGG | Gly | 0.657019 | TTT | Phe | 1.37374 | TTT | Phe | 1.38164 | TTT | Phe | 1.36447 |
| UUC | Phe | 0.6165 | GCC | Ala | 0.631268 | UUC | Phe | 0.641691 | UUC | Phe | 0.645594 | CCA | Pro | 1.18866 | CCA | Pro | 1.20322 | CCA | Pro | 1.18303 |
| UAG | Ter | 0.6 | UUC | Phe | 0.622361 | GCC | Ala | 0.617221 | GCC | Ala | 0.617747 | CCC | Pro | 0.70037 | CCC | Pro | 0.668008 | CCC | Pro | 0.722424 |
| AUC | Ile | 0.578334 | AUC | Ile | 0.572577 | AUC | Ile | 0.575292 | AUC | Ile | 0.577065 | CCG | Pro | 0.453761 | CCG | Pro | 0.49497 | CCG | Pro | 0.426667 |
| GUG | Val | 0.523397 | GUG | Val | 0.520275 | GUG | Val | 0.553114 | GUG | Val | 0.549451 | CCT | Pro | 1.65721 | CCT | Pro | 1.6338 | CCT | Pro | 1.66788 |
| GUC | Val | 0.495667 | GUC | Val | 0.517215 | GUC | Val | 0.505495 | GUC | Val | 0.509158 | AGC | Ser | 0.408683 | AGC | Ser | 0.378151 | AGC | Ser | 0.379412 |
| AGG | Arg | 0.472149 | UCG | Ser | 0.505141 | AGG | Arg | 0.486782 | AGG | Arg | 0.488564 | AGT | Ser | 1.20359 | AGT | Ser | 1.20648 | AGT | Ser | 1.20882 |
| CAC | His | 0.462243 | AGG | Arg | 0.483721 | GAG | Glu | 0.464475 | GAG | Glu | 0.466596 | TCA | Ser | 1.11826 | TCA | Ser | 1.15966 | TCA | Ser | 1.12059 |
| CCG | Pro | 0.461356 | CCG | Pro | 0.464819 | UCG | Ser | 0.464425 | UCG | Ser | 0.465882 | TCC | Ser | 0.848802 | TCC | Ser | 0.817527 | TCC | Ser | 0.873529 |
| GCG | Ala | 0.452145 | CAC | His | 0.462475 | CAC | His | 0.458629 | CCG | Pro | 0.459184 | TCG | Ser | 0.476048 | TCG | Ser | 0.47539 | TCG | Ser | 0.494118 |
| GAG | Glu | 0.451271 | GAG | Glu | 0.459193 | CCG | Pro | 0.453503 | CAC | His | 0.458234 | TCT | Ser | 1.94461 | TCT | Ser | 1.96279 | TCT | Ser | 1.92353 |
| GGC | Gly | 0.4426 | UGC | Cys | 0.459144 | GCG | Ala | 0.446718 | GCG | Ala | 0.450512 | TAA | Ter | 1.44444 | TAA | Ter | 1.33981 | TAA | Ter | 1.45361 |
| AAC | Asn | 0.440806 | GCG | Ala | 0.457227 | UGC | Cys | 0.446602 | UGC | Cys | 0.446602 | TAG | Ter | 0.666667 | TAG | Ter | 0.582524 | TAG | Ter | 0.71134 |
| UCG | Ser | 0.437923 | AAG | Lys | 0.44725 | GGC | Gly | 0.442197 | AAC | Asn | 0.43758 | TGA | Ter | 0.888889 | TGA | Ter | 1.07767 | TGA | Ter | 0.835052 |
| AAG | Lys | 0.429022 | GGC | Gly | 0.427689 | AAG | Lys | 0.437895 | AAG | Lys | 0.437367 | ACA | Thr | 1.14902 | ACA | Thr | 1.20562 | ACA | Thr | 1.16618 |
| UGC | Cys | 0.418182 | AAC | Asn | 0.424242 | AAC | Asn | 0.437018 | GGC | Gly | 0.437048 | ACC | Thr | 0.768627 | ACC | Thr | 0.746491 | ACC | Thr | 0.769679 |
| CAG | Gln | 0.414003 | CAG | Gln | 0.414698 | CAG | Gln | 0.422047 | CAG | Gln | 0.422535 | ACG | Thr | 0.403922 | ACG | Thr | 0.393064 | ACG | Thr | 0.396501 |
| UAC | Tyr | 0.394958 | CUC | Leu | 0.404801 | GAC | Asp | 0.401747 | GAC | Asp | 0.403509 | ACT | Thr | 1.67843 | ACT | Thr | 1.65483 | ACT | Thr | 1.66764 |
| AGC | Ser | 0.388262 | CGG | Arg | 0.404651 | AGC | Ser | 0.394058 | AGC | Ser | 0.395294 | TGG | Trp | 1 | TGG | Trp | 1 | TGG | Trp | 1 |
| CGG | Arg | 0.381963 | UAC | Tyr | 0.398093 | CGG | Arg | 0.382862 | CGG | Arg | 0.378774 | TAC | Tyr | 0.389356 | TAC | Tyr | 0.388889 | TAC | Tyr | 0.394993 |
| GAC | Asp | 0.381657 | GAC | Asp | 0.390606 | CUC | Leu | 0.376317 | CUC | Leu | 0.378 | TAT | Tyr | 1.61064 | TAT | Tyr | 1.61111 | TAT | Tyr | 1.60501 |
| ACG | Thr | 0.380762 | AGC | Ser | 0.385604 | UAC | Tyr | 0.371014 | UAC | Tyr | 0.372832 | GTA | Val | 1.45031 | GTA | Val | 1.42899 | GTA | Val | 1.44071 |
| CGC | Arg | 0.376658 | ACG | Thr | 0.382609 | ACG | Thr | 0.368794 | ACG | Thr | 0.369919 | GTC | Val | 0.515667 | GTC | Val | 0.54142 | GTC | Val | 0.513274 |
| CUC | Leu | 0.356322 | CGC | Arg | 0.353488 | CGC | Arg | 0.355515 | CGC | Arg | 0.356816 | GTG | Val | 0.56222 | GTG | Val | 0.497041 | GTG | Val | 0.559292 |
| CUG | Leu | 0.336207 | CUG | Leu | 0.340232 | CUG | Leu | 0.331159 | CUG | Leu | 0.333 | GTT | Val | 1.4718 | GTT | Val | 1.53254 | GTT | Val | 1.48673 |

**Supplementary Table 13.** Effective number of codons values of the genes from 5 Ericacea species mitochondrial genomes

| Species | GC all content （%） | GC1 content （%） | GC2 content （%） | GC3 content （%） | ENC | gene | ENC plot |
| --- | --- | --- | --- | --- | --- | --- | --- |
| MK990822 | 44.77 | 57.45 | 41.76 | 35.1 | 52.843 | *atp1* | 55.62046 |
| MK990822 | 43.35 | 47.15 | 42.49 | 40.41 | 60.993 | *atp4* | 58.34615 |
| MK990822 | 39.98 | 48.03 | 38.92 | 33 | 53.606 | *atp6* | 54.31996 |
| MK990822 | 40.62 | 36.25 | 39.38 | 46.25 | 56.755 | *atp8* | 60.13807 |
| MK990822 | 45.78 | 49.4 | 51.81 | 36.14 | 38.706 | *atp9* | 56.22271 |
| MK990822 | 41.38 | 45.41 | 45.89 | 32.85 | 51.453 | *ccmB* | 54.22315 |
| MK990822 | 43.56 | 47.01 | 48.61 | 35.06 | 49.599 | *ccmC* | 55.59671 |
| MK990822 | 45.43 | 49.44 | 44.77 | 42.09 | 58.574 | *ccmFc* | 59.00476 |
| MK990822 | 46.34 | 50.35 | 47.21 | 41.46 | 56.355 | *ccmFn* | 58.77055 |
| MK990822 | 40.36 | 47.46 | 40.1 | 33.5 | 52.702 | *cob* | 54.63909 |
| MK990822 | 42.8 | 47.35 | 43.18 | 37.88 | 55.15 | *cox1* | 57.15998 |
| MK990822 | 41.09 | 53.05 | 39.69 | 30.53 | 52.036 | *cox2* | 52.66859 |
| MK990822 | 41.98 | 51.13 | 40.98 | 33.83 | 54.763 | *cox3* | 54.84659 |
| MK990822 | 51.98 | 52.59 | 44.97 | 58.38 | 57.306 | *matR* | 58.99911 |
| MK990822 | 44.11 | 44.49 | 43.35 | 44.49 | 52.661 | *mttB* | 59.749 |
| MK990822 | 42.36 | 54.46 | 40.48 | 32.14 | 51.683 | *nad1* | 53.75845 |
| MK990822 | 39.74 | 41.31 | 43.97 | 33.95 | 51.446 | *nad2* | 54.92141 |
| MK990822 | 36.97 | 38.66 | 34.45 | 37.82 | 47.083 | *nad3* | 57.12922 |
| MK990822 | 36.97 | 38.66 | 34.45 | 37.82 | 47.083 | *nad3* | 57.12922 |
| MK990822 | 37.97 | 43.15 | 36.09 | 34.68 | 49.752 | *nad4* | 55.36902 |
| MK990822 | 38.61 | 40.59 | 42.57 | 32.67 | 42.199 | *nad4L* | 54.10633 |
| MK990822 | 40.8 | 42.65 | 42.33 | 37.42 | 55.564 | *nad5* | 56.92123 |
| MK990822 | 38.83 | 43.2 | 35.92 | 37.38 | 52.723 | *nad6* | 56.90016 |
| MK990822 | 44.39 | 55.44 | 44.56 | 33.16 | 51.252 | *nad7* | 54.42269 |
| MK990822 | 42.23 | 51.31 | 41.36 | 34.03 | 60.327 | *nad9* | 54.9711 |
| MK990822 | 42.23 | 51.31 | 41.36 | 34.03 | 60.327 | *nad9* | 54.9711 |
| MK990822 | 37.7 | 45.09 | 31.85 | 36.16 | 55.619 | *ORF671* | 56.23399 |
| MK990822 | 51.1 | 49.7 | 54.19 | 49.4 | 58.796 | *rpl2* | 60.48565 |
| MK990822 | 41.49 | 47.34 | 35.11 | 42.02 | 57.028 | *rpl5* | 58.97951 |
| MK990822 | 42.68 | 46.95 | 44.51 | 36.59 | 54.761 | *rpl10* | 56.47385 |
| MK990822 | 44.18 | 48.19 | 50.6 | 33.73 | 45.845 | *rpl16* | 54.78398 |
| MK990822 | 43.56 | 45.54 | 42.08 | 43.07 | 50.896 | *rps1* | 59.33752 |
| MK990822 | 42.49 | 44.5 | 40.6 | 42.38 | 59.973 | *rps3* | 59.10728 |
| MK990822 | 40.07 | 43.68 | 41.16 | 35.38 | 50.846 | *rps4* | 55.78551 |
| MK990822 | 40.27 | 44.97 | 37.58 | 38.26 | 58.137 | *rps10* | 57.35208 |
| MK990822 | 43.39 | 53.17 | 45.24 | 31.75 | 51.806 | *rps12* | 53.49886 |
| MK990822 | 43.39 | 53.17 | 45.24 | 31.75 | 51.806 | *rps12* | 53.49886 |
| MK990822 | 36.18 | 47.01 | 37.61 | 23.93 | 37.14 | *rps13* | 47.84188 |
| MK990822 | 39.65 | 36.84 | 47.37 | 34.74 | 57.272 | *rps19* | 55.40522 |
| MK990822 | 40 | 37.89 | 47.37 | 34.74 | 50.695 | *rps19* | 55.40522 |
| MK990822 | 37.38 | 37.38 | 36.45 | 38.32 | 45.909 | *sdh3* | 57.38197 |
| MK990822 | 40 | 41.33 | 34.67 | 44 | 50.797 | *sdh4* | 59.61666 |
| MZ779111 | 44.05 | 56.86 | 41.76 | 33.53 | 51.35 | *atp1* | 54.65805 |
| MZ779111 | 43.01 | 45.6 | 43.01 | 40.41 | 60.918 | *atp4* | 58.34615 |
| MZ779111 | 40.83 | 36.25 | 39.38 | 46.88 | 54.18 | *atp8* | 60.24384 |
| MZ779111 | 46.84 | 50.63 | 53.16 | 36.71 | 35.054 | *atp9* | 56.53982 |
| MZ779111 | 42.19 | 46.86 | 45.41 | 34.3 | 53.216 | *ccmB* | 55.13766 |
| MZ779111 | 42.14 | 45.71 | 45 | 35.71 | 46.268 | *ccmC* | 55.97731 |
| MZ779111 | 43.44 | 43.9 | 51.22 | 35 | 27.163 | *ccmC* | 55.56101 |
| MZ779111 | 47.46 | 52.73 | 47.66 | 41.96 | 58.456 | *ccmFc* | 58.95771 |
| MZ779111 | 46.26 | 51.03 | 46.72 | 41.03 | 56.464 | *ccmFn* | 58.60181 |
| MZ779111 | 42.42 | 47.49 | 43.49 | 36.27 | 54.092 | *cox1* | 56.29586 |
| MZ779111 | 40.67 | 52.9 | 39 | 30.12 | 49.438 | *cox2* | 52.38385 |
| MZ779111 | 42.23 | 51.5 | 40.98 | 34.21 | 55.003 | *cox3* | 55.08234 |
| MZ779111 | 39.29 | 47.23 | 37.45 | 33.19 | 52.014 | *cytB* | 54.44189 |
| MZ779111 | 51.39 | 53.73 | 44.08 | 56.36 | 56.487 | *matR* | 59.64011 |
| MZ779111 | 43.88 | 44.19 | 43.07 | 44.36 | 53.36 | *mttB* | 59.71489 |
| MZ779111 | 40.26 | 55.38 | 39.23 | 26.15 | 51.066 | *nad1* | 49.51089 |
| MZ779111 | 44.29 | 46.4 | 50.45 | 36.04 | 52.405 | *nad2* | 56.1661 |
| MZ779111 | 36.69 | 39.5 | 34.45 | 36.13 | 46.94 | *nad3* | 56.21706 |
| MZ779111 | 38.5 | 42.95 | 38.78 | 33.76 | 47.221 | *nad4* | 54.80278 |
| MZ779111 | 40.56 | 43.1 | 41.63 | 36.95 | 53.788 | *nad5* | 56.67046 |
| MZ779111 | 38.83 | 43.2 | 35.44 | 37.86 | 52.876 | *nad6* | 57.14974 |
| MZ779111 | 44.33 | 45.91 | 32.7 | 54.43 | 57.029 | *nad7* | 60.09255 |
| MZ779111 | 42.41 | 51.31 | 40.31 | 35.6 | 61 | *nad9* | 55.91371 |
| MZ779111 | 52.29 | 51.51 | 55.52 | 49.83 | 55.863 | *rpl2* | 60.49763 |
| MZ779111 | 42.07 | 46.95 | 43.9 | 35.37 | 59.107 | *rpl10* | 55.77965 |
| MZ779111 | 44.58 | 48.19 | 50.6 | 34.94 | 46.256 | *rpl16* | 55.52521 |
| MZ779111 | 43.3 | 47.42 | 41.75 | 40.72 | 50.855 | *rps1* | 58.47579 |
| MZ779111 | 39.47 | 42.24 | 41.16 | 35.02 | 51.621 | *rps4* | 55.57292 |
| MZ779111 | 39.39 | 43.64 | 39.09 | 35.45 | 52.469 | *rps10* | 55.82644 |
| MZ779111 | 43.65 | 52.38 | 45.24 | 33.33 | 53.254 | *rps12* | 54.53121 |
| MZ779111 | 35.85 | 44.54 | 38.66 | 24.37 | 38.571 | *rps13* | 48.17488 |
| MZ779111 | 40.96 | 47.87 | 35.11 | 39.89 | 56.052 | *rps15* | 58.12072 |
| MZ779111 | 39.65 | 38.95 | 47.37 | 32.63 | 51.579 | *rps19* | 54.08028 |
| MZ779111 | 38.23 | 39.39 | 32.58 | 42.75 | 49.201 | *sdh4* | 59.23316 |
| OM283814 | 45.56 | 56.86 | 41.96 | 37.84 | 53.965 | *atp1* | 57.13949 |
| OM283814 | 42.83 | 46.63 | 42.49 | 39.38 | 61 | *atp4* | 57.89015 |
| OM283814 | 39.04 | 46.5 | 37.76 | 32.87 | 56.964 | *atp6* | 54.23608 |
| OM283814 | 41.25 | 36.25 | 39.38 | 48.12 | 54.85 | *atp8* | 60.39932 |
| OM283814 | 44.72 | 47.56 | 51.22 | 35.37 | 33.447 | *atp9* | 55.77965 |
| OM283814 | 44.72 | 47.56 | 51.22 | 35.37 | 33.447 | *atp9* | 55.77965 |
| OM283814 | 41.71 | 45.89 | 45.41 | 33.82 | 53.787 | *ccmB* | 54.84034 |
| OM283814 | 43.88 | 46.76 | 47.12 | 37.77 | 49.962 | *ccmC* | 57.1035 |
| OM283814 | 45.18 | 48.34 | 44.81 | 42.38 | 58.916 | *ccmFC* | 59.10728 |
| OM283814 | 46.21 | 51.03 | 46.72 | 40.86 | 56.454 | *ccmFN* | 58.53315 |
| OM283814 | 40.19 | 47.97 | 39.85 | 32.74 | 52.876 | *cob* | 54.15184 |
| OM283814 | 42.34 | 47.07 | 42.91 | 37.05 | 54.551 | *cox1* | 56.72439 |
| OM283814 | 42.34 | 47.07 | 42.91 | 37.05 | 54.551 | *cox1* | 56.72439 |
| OM283814 | 46.51 | 56.19 | 40.95 | 42.38 | 57.139 | *cox2* | 59.10728 |
| OM283814 | 42.11 | 51.13 | 40.98 | 34.21 | 54.983 | *cox3* | 55.08234 |
| OM283814 | 51.68 | 51.98 | 45.27 | 57.77 | 57.764 | *matR* | 59.21008 |
| OM283814 | 43.5 | 43.97 | 42.55 | 43.97 | 52.974 | *mttB* | 59.60822 |
| OM283814 | 40.18 | 52.45 | 38.65 | 29.45 | 50.174 | *nad1* | 51.91291 |
| OM283814 | 39.6 | 40.7 | 43.76 | 34.36 | 52.187 | *nad2* | 55.17443 |
| OM283814 | 36.69 | 37.82 | 34.45 | 37.82 | 47.677 | *nad3* | 57.12922 |
| OM283814 | 38.31 | 43.75 | 36.09 | 35.08 | 50.566 | *nad4* | 55.60859 |
| OM283814 | 38.31 | 43.75 | 36.09 | 35.08 | 50.566 | *nad4* | 55.60859 |
| OM283814 | 38.83 | 41.76 | 42.86 | 31.87 | 45.168 | *nad4L* | 53.57905 |
| OM283814 | 40.97 | 44.67 | 42.63 | 35.62 | 52.822 | *nad5* | 55.9253 |
| OM283814 | 39.16 | 42.72 | 35.44 | 39.32 | 53.514 | *nad6* | 57.86242 |
| OM283814 | 43.63 | 55.19 | 44.56 | 31.14 | 49.221 | *nad7* | 53.08705 |
| OM283814 | 41.36 | 50.79 | 38.74 | 34.55 | 59.738 | *nad9* | 55.29028 |
| OM283814 | 41.67 | 48.4 | 35.64 | 40.96 | 56.051 | *rpl5* | 58.57367 |
| OM283814 | 42.68 | 47.56 | 43.9 | 36.59 | 61 | *rpl10* | 56.47385 |
| OM283814 | 43.43 | 45.87 | 40.37 | 44.04 | 50.106 | *rps1* | 59.62784 |
| OM283814 | 42.62 | 44.6 | 39.9 | 43.38 | 59.126 | *rps3* | 59.43459 |
| OM283814 | 39.45 | 40.97 | 41.26 | 36.1 | 52.172 | *rps4* | 56.2001 |
| OM283814 | 43.65 | 52.38 | 45.24 | 33.33 | 55.998 | *rps12* | 54.53121 |
| OM283814 | 42.22 | 51.11 | 28.89 | 46.67 | 49.976 | *rps14* | 60.21057 |
| OM283814 | 37.38 | 37.38 | 35.51 | 39.25 | 42.827 | *sdh3* | 57.82991 |
| OM283814 | 38.35 | 40.6 | 32.33 | 42.11 | 47.536 | *sdh4* | 59.01194 |
| NC_053763 | 45.69 | 56.86 | 41.96 | 38.24 | 53.892 | *atp1* | 57.34208 |
| NC_053763 | 42.83 | 46.63 | 42.49 | 39.38 | 61 | *atp4* | 57.89015 |
| NC_053763 | 42.83 | 46.63 | 42.49 | 39.38 | 61 | *atp4* | 57.89015 |
| NC_053763 | 39.04 | 46.5 | 37.76 | 32.87 | 56.964 | *atp6* | 54.23608 |
| NC_053763 | 41.25 | 36.25 | 39.38 | 48.12 | 54.85 | *atp8* | 60.39932 |
| NC_053763 | 44.72 | 47.56 | 51.22 | 35.37 | 33.447 | *atp9* | 55.77965 |
| NC_053763 | 41.71 | 45.89 | 45.41 | 33.82 | 53.787 | *ccmB* | 54.84034 |
| NC_053763 | 41.71 | 45.89 | 45.41 | 33.82 | 53.787 | *ccmB* | 54.84034 |
| NC_053763 | 43.88 | 46.76 | 47.12 | 37.77 | 49.962 | *ccmC* | 57.1035 |
| NC_053763 | 43.88 | 46.76 | 47.12 | 37.77 | 49.962 | *ccmC* | 57.1035 |
| NC_053763 | 45.18 | 48.34 | 44.81 | 42.38 | 58.916 | *ccmFC* | 59.10728 |
| NC_053763 | 46.21 | 51.03 | 46.72 | 40.86 | 56.454 | *ccmFN* | 58.53315 |
| NC_053763 | 40.27 | 47.97 | 39.85 | 32.99 | 52.949 | *cob* | 54.31352 |
| NC_053763 | 42.47 | 47.07 | 43.29 | 37.05 | 54.708 | *cox1* | 56.72439 |
| NC_053763 | 41.64 | 52.63 | 40.7 | 31.58 | 53.107 | *cox2* | 53.38479 |
| NC_053763 | 42.23 | 51.5 | 40.98 | 34.21 | 54.917 | *cox3* | 55.08234 |
| NC_053763 | 51.68 | 51.98 | 45.27 | 57.77 | 57.764 | *matR* | 59.21008 |
| NC_053763 | 43.5 | 43.97 | 42.55 | 43.97 | 52.974 | *mttB* | 59.60822 |
| NC_053763 | 43.5 | 43.97 | 42.55 | 43.97 | 52.974 | *mttB* | 59.60822 |
| NC_053763 | 40.18 | 52.45 | 38.65 | 29.45 | 50.174 | *nad1* | 51.91291 |
| NC_053763 | 39.54 | 40.7 | 43.76 | 34.15 | 52.096 | *nad2* | 55.04534 |
| NC_053763 | 36.69 | 37.82 | 34.45 | 37.82 | 47.677 | *nad3* | 57.12922 |
| NC_053763 | 38.24 | 43.75 | 36.09 | 34.88 | 50.554 | *nad4* | 55.48932 |
| NC_053763 | 38.83 | 41.76 | 42.86 | 31.87 | 45.168 | *nad4L* | 53.57905 |
| NC_053763 | 38.83 | 41.76 | 42.86 | 31.87 | 45.168 | *nad4L* | 53.57905 |
| NC_053763 | 40.97 | 44.67 | 42.63 | 35.62 | 52.822 | *nad5* | 55.9253 |
| NC_053763 | 39.16 | 42.72 | 35.44 | 39.32 | 53.514 | *nad6* | 57.86242 |
| NC_053763 | 43.63 | 55.19 | 44.56 | 31.14 | 49.221 | *nad7* | 53.08705 |
| NC_053763 | 41.36 | 50.79 | 38.74 | 34.55 | 59.738 | *nad9* | 55.29028 |
| NC_053763 | 41.36 | 50.79 | 38.74 | 34.55 | 59.738 | *nad9* | 55.29028 |
| NC_053763 | 41.67 | 48.4 | 35.64 | 40.96 | 56.051 | *rpl5* | 58.57367 |
| NC_053763 | 42.68 | 47.56 | 43.9 | 36.59 | 61 | *rpl10* | 56.47385 |
| NC_053763 | 42.68 | 47.56 | 43.9 | 36.59 | 61 | *rpl10* | 56.47385 |
| NC_053763 | 43.43 | 45.87 | 40.37 | 44.04 | 50.106 | *rps1* | 59.62784 |
| NC_053763 | 43.43 | 45.87 | 40.37 | 44.04 | 50.106 | *rps1* | 59.62784 |
| NC_053763 | 42.62 | 44.6 | 39.9 | 43.38 | 59.126 | *rps3* | 59.43459 |
| NC_053763 | 39.45 | 40.97 | 41.26 | 36.1 | 52.172 | *rps4* | 56.2001 |
| NC_053763 | 43.65 | 52.38 | 45.24 | 33.33 | 55.998 | *rps12* | 54.53121 |
| NC_053763 | 42.22 | 51.11 | 28.89 | 46.67 | 49.976 | *rps14* | 60.21057 |
| NC_053763 | 37.38 | 37.38 | 35.51 | 39.25 | 42.827 | *sdh3* | 57.82991 |
| NC_053763 | 38.35 | 40.6 | 32.33 | 42.11 | 47.536 | *sdh4* | 59.01194 |
| NC_023338 | 44.49 | 56.75 | 41.49 | 35.23 | 52.428 | *atp1* | 55.69735 |
| NC_023338 | 42.49 | 46.11 | 41.97 | 39.38 | 61 | *atp4* | 57.89015 |
| NC_023338 | 40.42 | 35.62 | 38.12 | 47.5 | 56.405 | *atp8* | 60.33036 |
| NC_023338 | 45.93 | 48.78 | 52.44 | 36.59 | 36.356 | *atp9* | 56.47385 |
| NC_023338 | 41.71 | 45.89 | 45.89 | 33.33 | 51.302 | *ccmB* | 54.53121 |
| NC_023338 | 43.89 | 45.05 | 45.54 | 41.09 | 54.509 | *ccmC* | 58.62578 |
| NC_023338 | 45.68 | 49.06 | 44.34 | 43.63 | 59.518 | *ccmFc* | 59.50995 |
| NC_023338 | 46.09 | 51.21 | 46.38 | 40.69 | 55.999 | *ccmFn* | 58.4634 |
| NC_023338 | 41.7 | 48.45 | 41.24 | 35.4 | 54.797 | *cob* | 55.79722 |
| NC_023338 | 42.15 | 47.49 | 43.29 | 35.67 | 52.753 | *cox1* | 55.95422 |
| NC_023338 | 40.47 | 52.53 | 38.13 | 30.74 | 51.081 | *cox2* | 52.81337 |
| NC_023338 | 41.98 | 51.13 | 40.98 | 33.83 | 54.762 | *cox3* | 54.84659 |
| NC_023338 | 50.56 | 52.72 | 43.51 | 55.44 | 56.978 | *matR* | 59.87586 |
| NC_023338 | 44.23 | 44.49 | 43.73 | 44.49 | 53.546 | *mttB* | 59.749 |
| NC_023338 | 42.42 | 54.55 | 40 | 32.73 | 52.128 | *nad1* | 54.14535 |
| NC_023338 | 40.24 | 41.06 | 44.92 | 34.76 | 52.627 | *nad2* | 55.41727 |
| NC_023338 | 36.13 | 37.82 | 34.45 | 36.13 | 48.621 | *nad3* | 56.21706 |
| NC_023338 | 37.98 | 43.43 | 36.16 | 34.34 | 49.362 | *nad4* | 55.16218 |
| NC_023338 | 39.87 | 43.29 | 41.33 | 34.99 | 51.853 | *nad5* | 55.55505 |
| NC_023338 | 39.16 | 42.72 | 35.92 | 38.83 | 52.944 | *nad6* | 57.63126 |
| NC_023338 | 44.22 | 55.95 | 44.56 | 32.15 | 50.496 | *nad7* | 53.76507 |
| NC_023338 | 41.54 | 50.79 | 39.79 | 34.03 | 59.413 | *nad9* | 54.9711 |
| NC_023338 | 51.09 | 50.6 | 53.27 | 49.4 | 60.064 | *rpl2* | 60.48565 |
| NC_023338 | 42.68 | 47.56 | 43.9 | 36.59 | 60.005 | *rpl10* | 56.47385 |
| NC_023338 | 44.58 | 48.19 | 50.6 | 34.94 | 46.256 | *rpl16* | 55.52521 |
| NC_023338 | 42.88 | 45.73 | 39.7 | 43.22 | 52.245 | *rps1* | 59.38499 |
| NC_023338 | 40.31 | 43.32 | 41.88 | 35.74 | 53.181 | *rps4* | 55.9946 |
| NC_023338 | 39.39 | 43.64 | 39.09 | 35.45 | 53.213 | *rps10* | 55.82644 |
| NC_023338 | 42.33 | 52.38 | 45.24 | 29.37 | 51.14 | *rps12* | 51.85624 |
| NC_023338 | 34.76 | 44.44 | 37.61 | 22.22 | 40.294 | *rps13* | 46.54128 |
| NC_023338 | 41.58 | 48.39 | 35.48 | 40.86 | 56.098 | *rps15* | 58.53315 |
| NC_023338 | 40 | 38.95 | 47.37 | 33.68 | 52.842 | *rps19* | 54.75258 |
| NC_023338 | 38.6 | 39.85 | 32.33 | 43.61 | 50.53 | *sdh4* | 59.50402 |

**Supplementary Table 14.** Effective number of codons values of the genes from 15 *Rhododendron* species chloroplast genomes

| Species | GC all content （%） | GC1 content （%） | GC2 content （%） | GC3 content （%） | ENC | gene | ENC plot | ENC plot -ENC |
| --- | --- | --- | --- | --- | --- | --- | --- | --- |
| MN711645 | 40.02 | 50.56 | 36.72 | 32.77 | 53.447 | *accD* | 54.17131 | 0.724314345 |
| MN711645 | 40.09 | 54.33 | 40.16 | 25.79 | 44.771 | *atpA* | 49.2424 | 4.47140072 |
| MN711645 | 41.82 | 56.11 | 41.68 | 27.66 | 46.317 | *atpB* | 50.62483 | 4.307831035 |
| MN711645 | 38.31 | 49.25 | 38.06 | 27.61 | 47.054 | *atpE* | 50.5883 | 3.534303186 |
| MN711645 | 38.02 | 45.95 | 34.59 | 33.51 | 45.492 | *atpF* | 54.64541 | 9.153410099 |
| MN711645 | 45.93 | 60.98 | 48.78 | 28.05 | 51.174 | *atpH* | 50.9088 | -0.265195476 |
| MN711645 | 38.58 | 49.19 | 37.1 | 29.44 | 46.049 | *atpI* | 51.90583 | 5.856829597 |
| MN711645 | 33.44 | 35.51 | 37.07 | 27.73 | 47.907 | *ccsA* | 50.67592 | 2.768924691 |
| MN711645 | 33.44 | 35.51 | 37.07 | 27.73 | 47.907 | *ccsA* | 50.67592 | 2.768924691 |
| MN711645 | 33.33 | 39.13 | 28.26 | 32.61 | 46.447 | *cemA* | 54.06724 | 7.620241141 |
| MN711645 | 33.33 | 39.13 | 28.26 | 32.61 | 46.447 | *cemA* | 54.06724 | 7.620241141 |
| MN711645 | 36.99 | 47.56 | 37.8 | 25.61 | 54.141 | *infA* | 49.10779 | -5.033206193 |
| MN711645 | 32.74 | 39.25 | 31.56 | 27.42 | 47.374 | *matK* | 50.44926 | 3.075255793 |
| MN711645 | 34.16 | 40.93 | 39.56 | 21.98 | 41.646 | *ndhA* | 46.35821 | 4.712212705 |
| MN711645 | 34.16 | 40.93 | 39.56 | 21.98 | 41.646 | *ndhA* | 46.35821 | 4.712212705 |
| MN711645 | 37.19 | 41.58 | 37.93 | 32.05 | 46.918 | *ndhB* | 53.69881 | 6.780811797 |
| MN711645 | 34.99 | 47.11 | 31.4 | 26.45 | 56.087 | *ndhC* | 49.73385 | -6.353148423 |
| MN711645 | 35.44 | 40.43 | 37.48 | 28.4 | 45.366 | *ndhD* | 51.16216 | 5.796161911 |
| MN711645 | 35.44 | 40.43 | 37.48 | 28.4 | 45.366 | *ndhD* | 51.16216 | 5.796161911 |
| MN711645 | 33.33 | 40.2 | 34.31 | 25.49 | 39.896 | *ndhE* | 49.01793 | 9.121929252 |
| MN711645 | 33.33 | 40.2 | 34.31 | 25.49 | 39.896 | *ndhE* | 49.01793 | 9.121929252 |
| MN711645 | 32.13 | 36.1 | 35.7 | 24.6 | 42.64 | *ndhF* | 48.34858 | 5.708583016 |
| MN711645 | 32.77 | 41.57 | 33.71 | 23.03 | 47.492 | *ndhG* | 47.15837 | -0.333628448 |
| MN711645 | 32.77 | 41.57 | 33.71 | 23.03 | 47.492 | *ndhG* | 47.15837 | -0.333628448 |
| MN711645 | 37.65 | 51.02 | 36.55 | 25.38 | 49.275 | *ndhH* | 48.93547 | -0.339532528 |
| MN711645 | 37.65 | 51.02 | 36.55 | 25.38 | 49.275 | *ndhH* | 48.93547 | -0.339532528 |
| MN711645 | 35.77 | 42.7 | 36.52 | 28.09 | 48.067 | *ndhI* | 50.93783 | 2.870832752 |
| MN711645 | 35.77 | 42.7 | 36.52 | 28.09 | 48.067 | *ndhI* | 50.93783 | 2.870832752 |
| MN711645 | 38.99 | 50.31 | 37.11 | 29.56 | 46.076 | *ndhJ* | 51.99068 | 5.914684669 |
| MN711645 | 37.17 | 43.81 | 42.48 | 25.22 | 51.175 | *ndhK* | 48.81538 | -2.359618773 |
| MN711645 | 40.19 | 52.02 | 36.45 | 32.09 | 49.848 | *petA* | 53.72534 | 3.877338387 |
| MN711645 | 40.19 | 52.02 | 36.45 | 32.09 | 49.848 | *petA* | 53.72534 | 3.877338387 |
| MN711645 | 39.51 | 48.61 | 42.13 | 27.78 | 44.342 | *petB* | 50.71239 | 6.370387453 |
| MN711645 | 37.71 | 50.62 | 39.38 | 23.12 | 45.237 | *petD* | 47.22684 | 1.989840698 |
| MN711645 | 34.21 | 50 | 28.95 | 23.68 | 43.133 | *petG* | 47.6523 | 4.519302359 |
| MN711645 | 32.29 | 31.25 | 43.75 | 21.88 | 55.5 | *petL* | 46.28191 | -9.218087523 |
| MN711645 | 41.11 | 50 | 43.33 | 30 | 30.698 | *petN* | 52.3 | 21.602 |
| MN711645 | 42.7 | 52.2 | 43.14 | 32.76 | 50.489 | *psaA* | 54.16483 | 3.675825677 |
| MN711645 | 40.82 | 48.44 | 43.13 | 30.88 | 48.181 | *psaB* | 52.90948 | 4.728475383 |
| MN711645 | 41.87 | 45.12 | 53.66 | 26.83 | 52.531 | *psaC* | 50.01516 | -2.515839759 |
| MN711645 | 41.87 | 45.12 | 53.66 | 26.83 | 52.531 | *psaC* | 50.01516 | -2.515839759 |
| MN711645 | 32.43 | 40.54 | 29.73 | 27.03 | 41.643 | *psaI* | 50.16269 | 8.519689698 |
| MN711645 | 32.43 | 40.54 | 29.73 | 27.03 | 41.643 | *psaI* | 50.16269 | 8.519689698 |
| MN711645 | 41.34 | 49.44 | 43.79 | 30.79 | 41.566 | *psbA* | 52.84773 | 11.28173339 |
| MN711645 | 44.01 | 54.62 | 45.97 | 31.43 | 48.539 | *psbB* | 53.28369 | 4.744686767 |
| MN711645 | 44.16 | 54.01 | 46.41 | 32.07 | 45.006 | *psbC* | 53.71208 | 8.706079057 |
| MN711645 | 42.56 | 51.98 | 43.22 | 32.49 | 46.188 | *psbD* | 53.98883 | 7.80083317 |
| MN711645 | 41.67 | 44.05 | 47.62 | 33.33 | 50.06 | *psbE* | 54.53121 | 4.471211875 |
| MN711645 | 39.17 | 47.5 | 45 | 25 | 44 | *psbF* | 48.65 | 4.65 |
| MN711645 | 37.39 | 36.49 | 47.3 | 28.38 | 42.805 | *psbH* | 51.14772 | 8.34272388 |
| MN711645 | 37.84 | 54.05 | 32.43 | 27.03 | 27.272 | *psbI* | 50.16269 | 22.8906897 |
| MN711645 | 41.46 | 46.34 | 48.78 | 29.27 | 35.964 | *psbJ* | 51.78527 | 15.82126637 |
| MN711645 | 36.02 | 38.71 | 33.87 | 35.48 | 51.871 | *psbK* | 55.84394 | 3.972944876 |
| MN711645 | 32.48 | 35.9 | 25.64 | 35.9 | 52.793 | *psbL* | 56.08638 | 3.293383551 |
| MN711645 | 29.52 | 45.71 | 25.71 | 17.14 | 47 | *psbM* | 42.67669 | -4.323313026 |
| MN711645 | 42.42 | 54.55 | 40.91 | 31.82 | 46.02 | *psbN* | 53.54567 | 7.525673867 |
| MN711645 | 34.26 | 38.89 | 30.56 | 33.33 | 56.893 | *psbT* | 54.53121 | -2.361788125 |
| MN711645 | 36.51 | 41.27 | 41.27 | 26.98 | 46.925 | *psbZ* | 50.12584 | 3.200842687 |
| MN711645 | 43.56 | 58.61 | 43.07 | 28.99 | 46.912 | *rbcL* | 51.58582 | 4.673816622 |
| MN711645 | 42.29 | 48.51 | 48.51 | 29.85 | 50.37 | *rpl2* | 52.19488 | 1.824878985 |
| MN711645 | 39.39 | 56.06 | 34.85 | 27.27 | 50.124 | *rpl14* | 50.33922 | 0.215217696 |
| MN711645 | 38.6 | 37.72 | 49.12 | 28.95 | 44.87 | *rpl20* | 51.55724 | 6.687237022 |
| MN711645 | 34.41 | 39.35 | 39.35 | 24.52 | 42.675 | *rpl22* | 48.2882 | 5.613195139 |
| MN711645 | 32.64 | 40.62 | 33.33 | 23.96 | 49.603 | *rpl23* | 47.86461 | -1.738391504 |
| MN711645 | 35.78 | 35.29 | 48.53 | 23.53 | 47.675 | *rpl32* | 47.53844 | -0.136556515 |
| MN711645 | 35.78 | 35.29 | 48.53 | 23.53 | 47.675 | *rpl32* | 47.53844 | -0.136556515 |
| MN711645 | 37.56 | 38.03 | 36.62 | 38.03 | 52.036 | *rpl33* | 57.23637 | 5.200369213 |
| MN711645 | 36.84 | 36.84 | 50 | 23.68 | 26.489 | *rpl36* | 47.6523 | 21.16330236 |
| MN711645 | 34.65 | 43.17 | 35.61 | 25.18 | 45.309 | *rpoA* | 48.78533 | 3.476334119 |
| MN711645 | 37.89 | 48.69 | 36.7 | 28.28 | 48.406 | *rpoB* | 51.07546 | 2.669460938 |
| MN711645 | 37.69 | 49.42 | 36.48 | 27.18 | 48.284 | *rpoC1* | 50.27309 | 1.989086037 |
| MN711645 | 36.91 | 45.44 | 36.76 | 28.52 | 49.628 | *rpoC2* | 51.24869 | 1.620686881 |
| MN711645 | 37.43 | 41.95 | 40.68 | 29.66 | 50.53 | *rps2* | 52.06124 | 1.53123729 |
| MN711645 | 34.2 | 42.81 | 36.6 | 23.2 | 48.689 | *rps3* | 47.28768 | -1.401317391 |
| MN711645 | 37.16 | 46.89 | 38.76 | 25.84 | 48.651 | *rps4* | 49.27975 | 0.628749898 |
| MN711645 | 39.53 | 51.28 | 42.31 | 25 | 46.355 | *rps7* | 48.65 | 2.295 |
| MN711645 | 34.09 | 39.85 | 39.85 | 22.56 | 44.946 | *rps8* | 46.80047 | 1.854474969 |
| MN711645 | 41.49 | 48.92 | 53.96 | 21.58 | 51.001 | *rps11* | 46.05296 | -4.948044933 |
| MN711645 | 41.94 | 50 | 46.77 | 29.03 | 44.033 | *rps12* | 51.61437 | 7.581374785 |
| MN711645 | 42.9 | 47.52 | 47.52 | 33.66 | 43.475 | *rps14* | 54.74001 | 11.26500823 |
| MN711645 | 31.9 | 43.01 | 24.73 | 27.96 | 55.317 | *rps15* | 50.84342 | -4.473576284 |
| MN711645 | 31.9 | 43.01 | 24.73 | 27.96 | 55.317 | *rps15* | 50.84342 | -4.473576284 |
| MN711645 | 34.47 | 51.14 | 31.82 | 20.45 | 50.19 | *rps16* | 45.19036 | -4.999643088 |
| MN711645 | 34.47 | 51.14 | 31.82 | 20.45 | 50.19 | *rps16* | 45.19036 | -4.999643088 |
| MN711645 | 33.03 | 33.33 | 40.54 | 25.23 | 38.567 | *rps18* | 48.82289 | 10.25589143 |
| MN711645 | 38.46 | 47.93 | 38.46 | 28.99 | 51.943 | *ycf3* | 51.58582 | -0.357183378 |
| MN711645 | 38.92 | 48.11 | 40 | 28.65 | 50.179 | *ycf4* | 51.34222 | 1.163219685 |
| MN711645 | 38.92 | 48.11 | 40 | 28.65 | 50.179 | *ycf4* | 51.34222 | 1.163219685 |
| MT239363 | 40.09 | 54.13 | 40.16 | 25.98 | 45.019 | *atpA* | 49.38423 | 4.365228381 |
| MT239363 | 41.82 | 56.11 | 41.68 | 27.66 | 46.317 | *atpB* | 50.62483 | 4.307831035 |
| MT239363 | 38.31 | 49.25 | 38.06 | 27.61 | 47.054 | *atpE* | 50.5883 | 3.534303186 |
| MT239363 | 38.02 | 45.95 | 34.59 | 33.51 | 45.492 | *atpF* | 54.64541 | 9.153410099 |
| MT239363 | 45.93 | 60.98 | 48.78 | 28.05 | 51.174 | *atpH* | 50.9088 | -0.265195476 |
| MT239363 | 38.58 | 49.19 | 37.1 | 29.44 | 46.049 | *atpI* | 51.90583 | 5.856829597 |
| MT239363 | 33.33 | 35.51 | 37.07 | 27.41 | 47.843 | *ccsA* | 50.44193 | 2.598927074 |
| MT239363 | 33.33 | 35.51 | 37.07 | 27.41 | 47.843 | *ccsA* | 50.44193 | 2.598927074 |
| MT239363 | 33.33 | 39.13 | 28.26 | 32.61 | 46.447 | *cemA* | 54.06724 | 7.620241141 |
| MT239363 | 33.33 | 39.13 | 28.26 | 32.61 | 46.447 | *cemA* | 54.06724 | 7.620241141 |
| MT239363 | 32.54 | 38.86 | 31.56 | 27.22 | 47.189 | *matK* | 50.30249 | 3.113487833 |
| MT239363 | 34.16 | 40.93 | 39.56 | 21.98 | 41.646 | *ndhA* | 46.35821 | 4.712212705 |
| MT239363 | 34.16 | 40.93 | 39.56 | 21.98 | 41.646 | *ndhA* | 46.35821 | 4.712212705 |
| MT239363 | 37.05 | 41.49 | 37.57 | 32.09 | 47.417 | *ndhB* | 53.72534 | 6.308338387 |
| MT239363 | 34.99 | 47.11 | 31.4 | 26.45 | 56.087 | *ndhC* | 49.73385 | -6.353148423 |
| MT239363 | 35.44 | 40.43 | 37.48 | 28.4 | 45.366 | *ndhD* | 51.16216 | 5.796161911 |
| MT239363 | 35.44 | 40.43 | 37.48 | 28.4 | 45.366 | *ndhD* | 51.16216 | 5.796161911 |
| MT239363 | 33.33 | 40.2 | 34.31 | 25.49 | 39.896 | *ndhE* | 49.01793 | 9.121929252 |
| MT239363 | 33.33 | 40.2 | 34.31 | 25.49 | 39.896 | *ndhE* | 49.01793 | 9.121929252 |
| MT239363 | 32.04 | 35.96 | 35.83 | 24.33 | 42.54 | *ndhF* | 48.14464 | 5.604643957 |
| MT239363 | 32.77 | 41.57 | 33.71 | 23.03 | 47.492 | *ndhG* | 47.15837 | -0.333628448 |
| MT239363 | 32.77 | 41.57 | 33.71 | 23.03 | 47.492 | *ndhG* | 47.15837 | -0.333628448 |
| MT239363 | 37.65 | 51.02 | 36.55 | 25.38 | 49.275 | *ndhH* | 48.93547 | -0.339532528 |
| MT239363 | 37.65 | 51.02 | 36.55 | 25.38 | 49.275 | *ndhH* | 48.93547 | -0.339532528 |
| MT239363 | 35.77 | 42.7 | 36.52 | 28.09 | 48.067 | *ndhI* | 50.93783 | 2.870832752 |
| MT239363 | 35.77 | 42.7 | 36.52 | 28.09 | 48.067 | *ndhI* | 50.93783 | 2.870832752 |
| MT239363 | 38.78 | 50.31 | 37.11 | 28.93 | 45.032 | *ndhJ* | 51.54294 | 6.510939224 |
| MT239363 | 37.32 | 43.81 | 42.48 | 25.66 | 51.108 | *ndhK* | 49.14521 | -1.962792198 |
| MT239363 | 40.29 | 52.02 | 36.45 | 32.4 | 50.157 | *petA* | 53.92983 | 3.772831103 |
| MT239363 | 40.29 | 52.02 | 36.45 | 32.4 | 50.157 | *petA* | 53.92983 | 3.772831103 |
| MT239363 | 39.51 | 48.61 | 42.13 | 27.78 | 44.342 | *petB* | 50.71239 | 6.370387453 |
| MT239363 | 37.47 | 50.31 | 39.13 | 22.98 | 45.475 | *petD* | 47.12032 | 1.645323468 |
| MT239363 | 34.21 | 50 | 28.95 | 23.68 | 43.133 | *petG* | 47.6523 | 4.519302359 |
| MT239363 | 32.29 | 31.25 | 43.75 | 21.88 | 55.5 | *petL* | 46.28191 | -9.218087523 |
| MT239363 | 41.11 | 50 | 43.33 | 30 | 30.698 | *petN* | 52.3 | 21.602 |
| MT239363 | 42.7 | 52.2 | 43.14 | 32.76 | 50.489 | *psaA* | 54.16483 | 3.675825677 |
| MT239363 | 40.73 | 48.44 | 43.13 | 30.61 | 47.879 | *psaB* | 52.72384 | 4.844835276 |
| MT239363 | 41.87 | 45.12 | 53.66 | 26.83 | 51.756 | *psaC* | 50.01516 | -1.740839759 |
| MT239363 | 41.87 | 45.12 | 53.66 | 26.83 | 51.756 | *psaC* | 50.01516 | -1.740839759 |
| MT239363 | 32.43 | 40.54 | 29.73 | 27.03 | 41.643 | *psaI* | 50.16269 | 8.519689698 |
| MT239363 | 32.43 | 40.54 | 29.73 | 27.03 | 41.643 | *psaI* | 50.16269 | 8.519689698 |
| MT239363 | 37.59 | 40.43 | 42.55 | 29.79 | 39.036 | *psaJ* | 52.15273 | 13.11673476 |
| MT239363 | 41.43 | 49.44 | 43.79 | 31.07 | 41.524 | *psbA* | 53.03936 | 11.51535769 |
| MT239363 | 44.01 | 54.62 | 45.97 | 31.43 | 48.539 | *psbB* | 53.28369 | 4.744686767 |
| MT239363 | 44.09 | 54.01 | 46.41 | 31.86 | 45.106 | *psbC* | 53.57238 | 8.466380585 |
| MT239363 | 42.47 | 51.98 | 43.22 | 32.2 | 46.148 | *psbD* | 53.79812 | 7.650122179 |
| MT239363 | 41.67 | 44.05 | 47.62 | 33.33 | 50.06 | *psbE* | 54.53121 | 4.471211875 |
| MT239363 | 39.17 | 47.5 | 45 | 25 | 44 | *psbF* | 48.65 | 4.65 |
| MT239363 | 41.46 | 46.34 | 48.78 | 29.27 | 35.964 | *psbJ* | 51.78527 | 15.82126637 |
| MT239363 | 36.02 | 38.71 | 33.87 | 35.48 | 54.824 | *psbK* | 55.84394 | 1.019944876 |
| MT239363 | 32.48 | 35.9 | 25.64 | 35.9 | 52.793 | *psbL* | 56.08638 | 3.293383551 |
| MT239363 | 29.52 | 45.71 | 25.71 | 17.14 | 47 | *psbM* | 42.67669 | -4.323313026 |
| MT239363 | 42.42 | 54.55 | 40.91 | 31.82 | 46.02 | *psbN* | 53.54567 | 7.525673867 |
| MT239363 | 36.51 | 41.27 | 41.27 | 26.98 | 46.925 | *psbZ* | 50.12584 | 3.200842687 |
| MT239363 | 43.77 | 58.61 | 43.49 | 29.2 | 47.033 | *rbcL* | 51.7355 | 4.702504828 |
| MT239363 | 42.16 | 48.51 | 48.51 | 29.48 | 49.344 | *rpl2* | 51.93414 | 2.590137689 |
| MT239363 | 39.39 | 56.06 | 34.85 | 27.27 | 50.124 | *rpl14* | 50.33922 | 0.215217696 |
| MT239363 | 39.9 | 47.45 | 51.82 | 20.44 | 37.45 | *rpl16* | 45.18273 | 7.732725645 |
| MT239363 | 38.3 | 37.72 | 48.25 | 28.95 | 43.529 | *rpl20* | 51.55724 | 8.028237022 |
| MT239363 | 34.19 | 39.35 | 38.71 | 24.52 | 42.796 | *rpl22* | 48.2882 | 5.492195139 |
| MT239363 | 32.62 | 40.86 | 34.41 | 22.58 | 48.969 | *rpl23* | 46.81571 | -2.153285144 |
| MT239363 | 35.78 | 35.29 | 48.53 | 23.53 | 47.675 | *rpl32* | 47.53844 | -0.136556515 |
| MT239363 | 35.78 | 35.29 | 48.53 | 23.53 | 47.675 | *rpl32* | 47.53844 | -0.136556515 |
| MT239363 | 36.99 | 38.36 | 35.62 | 36.99 | 50.543 | *rpl33* | 56.69207 | 6.149069653 |
| MT239363 | 36.84 | 36.84 | 50 | 23.68 | 26.489 | *rpl36* | 47.6523 | 21.16330236 |
| MT239363 | 34.41 | 43.17 | 35.25 | 24.82 | 45.162 | *rpoA* | 48.51447 | 3.352473444 |
| MT239363 | 38.05 | 48.69 | 36.89 | 28.56 | 48.613 | *rpoB* | 51.27749 | 2.664488835 |
| MT239363 | 37.79 | 49.56 | 36.48 | 27.33 | 48.358 | *rpoC1* | 50.38326 | 2.025260391 |
| MT239363 | 36.91 | 45.44 | 36.69 | 28.59 | 49.653 | *rpoC2* | 51.29908 | 1.646077142 |
| MT239363 | 37.29 | 41.95 | 40.25 | 29.66 | 50.105 | *rps2* | 52.06124 | 1.95623729 |
| MT239363 | 35.01 | 44.77 | 33.89 | 26.36 | 46.821 | *rps3* | 49.66704 | 2.846042131 |
| MT239363 | 37.16 | 46.89 | 38.76 | 25.84 | 48.416 | *rps4* | 49.27975 | 0.863749898 |
| MT239363 | 39.53 | 51.28 | 42.31 | 25 | 46.355 | *rps7* | 48.65 | 2.295 |
| MT239363 | 33.83 | 39.85 | 39.85 | 21.8 | 40.338 | *rps8* | 46.22086 | 5.882864738 |
| MT239363 | 41.73 | 49.64 | 53.96 | 21.58 | 50.703 | *rps11* | 46.05296 | -4.650044933 |
| MT239363 | 42.2 | 50.81 | 46.77 | 29.03 | 44.647 | *rps12* | 51.61437 | 6.967374785 |
| MT239363 | 42.9 | 47.52 | 47.52 | 33.66 | 43.475 | *rps14* | 54.74001 | 11.26500823 |
| MT239363 | 31.9 | 43.01 | 24.73 | 27.96 | 55.317 | *rps15* | 50.84342 | -4.473576284 |
| MT239363 | 31.9 | 43.01 | 24.73 | 27.96 | 55.317 | *rps15* | 50.84342 | -4.473576284 |
| MT239363 | 34.47 | 51.14 | 31.82 | 20.45 | 50.19 | *rps16* | 45.19036 | -4.999643088 |
| MT239363 | 34.47 | 51.14 | 31.82 | 20.45 | 50.19 | *rps16* | 45.19036 | -4.999643088 |
| MT239363 | 33.04 | 32.74 | 40.71 | 25.66 | 38.032 | *rps18* | 49.14521 | 11.1132078 |
| MT239363 | 33.69 | 43.01 | 36.56 | 21.51 | 54.586 | *rps19* | 45.99952 | -8.586477706 |
| MT239363 | 38.46 | 47.93 | 38.46 | 28.99 | 51.943 | *ycf3* | 51.58582 | -0.357183378 |
| MT239363 | 38.92 | 48.11 | 40 | 28.65 | 50.179 | *ycf4* | 51.34222 | 1.163219685 |
| MT239363 | 38.92 | 48.11 | 40 | 28.65 | 50.179 | *ycf4* | 51.34222 | 1.163219685 |
| MT239365 | 40.03 | 54.33 | 40.16 | 25.59 | 44.792 | *atpA* | 49.09282 | 4.300823297 |
| MT239365 | 41.75 | 56.31 | 41.68 | 27.25 | 45.718 | *atpB* | 50.32453 | 4.606528748 |
| MT239365 | 38.56 | 49.25 | 38.06 | 28.36 | 46.088 | *atpE* | 51.13328 | 5.045280975 |
| MT239365 | 38.02 | 45.95 | 34.59 | 33.51 | 45.406 | *atpF* | 54.64541 | 9.239410099 |
| MT239365 | 45.53 | 60.98 | 48.78 | 26.83 | 50.468 | *atpH* | 50.01516 | -0.452839759 |
| MT239365 | 39.11 | 49.6 | 37.1 | 30.65 | 46.008 | *atpI* | 52.75142 | 6.743415457 |
| MT239365 | 33.44 | 35.51 | 37.38 | 27.41 | 47.949 | *ccsA* | 50.44193 | 2.492927074 |
| MT239365 | 33.44 | 35.51 | 37.38 | 27.41 | 47.949 | *ccsA* | 50.44193 | 2.492927074 |
| MT239365 | 33.48 | 39.57 | 28.26 | 32.61 | 46.464 | *cemA* | 54.06724 | 7.603241141 |
| MT239365 | 33.48 | 39.57 | 28.26 | 32.61 | 46.464 | *cemA* | 54.06724 | 7.603241141 |
| MT239365 | 32.41 | 38.19 | 31.5 | 27.56 | 47.675 | *matK* | 50.55175 | 2.876748626 |
| MT239365 | 34.16 | 40.93 | 39.56 | 21.98 | 41.646 | *ndhA* | 46.35821 | 4.712212705 |
| MT239365 | 34.16 | 40.93 | 39.56 | 21.98 | 41.646 | *ndhA* | 46.35821 | 4.712212705 |
| MT239365 | 36.92 | 41.49 | 37.57 | 31.7 | 46.86 | *ndhB* | 53.46537 | 6.605369073 |
| MT239365 | 35.26 | 47.11 | 31.4 | 27.27 | 56.338 | *ndhC* | 50.33922 | -5.998782304 |
| MT239365 | 35.57 | 40.43 | 37.48 | 28.8 | 45.433 | *ndhD* | 51.44987 | 6.016874797 |
| MT239365 | 35.57 | 40.43 | 37.48 | 28.8 | 45.433 | *ndhD* | 51.44987 | 6.016874797 |
| MT239365 | 33.33 | 40.2 | 34.31 | 25.49 | 39.896 | *ndhE* | 49.01793 | 9.121929252 |
| MT239365 | 33.33 | 40.2 | 34.31 | 25.49 | 39.896 | *ndhE* | 49.01793 | 9.121929252 |
| MT239365 | 31.73 | 35.83 | 35.56 | 23.8 | 42.932 | *ndhF* | 47.74333 | 4.811328831 |
| MT239365 | 32.77 | 41.57 | 33.71 | 23.03 | 47.492 | *ndhG* | 47.15837 | -0.333628448 |
| MT239365 | 32.77 | 41.57 | 33.71 | 23.03 | 47.492 | *ndhG* | 47.15837 | -0.333628448 |
| MT239365 | 37.87 | 51.41 | 36.76 | 25.45 | 49.549 | *ndhH* | 48.98795 | -0.56104747 |
| MT239365 | 37.87 | 51.41 | 36.76 | 25.45 | 49.549 | *ndhH* | 48.98795 | -0.56104747 |
| MT239365 | 35.77 | 42.7 | 36.52 | 28.09 | 48.067 | *ndhI* | 50.93783 | 2.870832752 |
| MT239365 | 35.77 | 42.7 | 36.52 | 28.09 | 48.067 | *ndhI* | 50.93783 | 2.870832752 |
| MT239365 | 38.57 | 50.31 | 35.85 | 29.56 | 46.23 | *ndhJ* | 51.99068 | 5.760684669 |
| MT239365 | 37.32 | 44.25 | 42.48 | 25.22 | 50.448 | *ndhK* | 48.81538 | -1.632618773 |
| MT239365 | 40.19 | 52.02 | 36.45 | 32.09 | 49.848 | *petA* | 53.72534 | 3.877338387 |
| MT239365 | 40.19 | 52.02 | 36.45 | 32.09 | 49.848 | *petA* | 53.72534 | 3.877338387 |
| MT239365 | 39.81 | 48.61 | 42.13 | 28.7 | 44.715 | *petB* | 51.37814 | 6.663136849 |
| MT239365 | 37.47 | 50.31 | 39.13 | 22.98 | 46.058 | *petD* | 47.12032 | 1.062323468 |
| MT239365 | 34.21 | 50 | 28.95 | 23.68 | 43.133 | *petG* | 47.6523 | 4.519302359 |
| MT239365 | 32.29 | 31.25 | 43.75 | 21.88 | 55.5 | *petL* | 46.28191 | -9.218087523 |
| MT239365 | 41.11 | 50 | 43.33 | 30 | 30.698 | *petN* | 52.3 | 21.602 |
| MT239365 | 42.65 | 52.06 | 43.14 | 32.76 | 50.581 | *psaA* | 54.16483 | 3.583825677 |
| MT239365 | 40.86 | 48.44 | 43.13 | 31.02 | 48.513 | *psaB* | 53.00524 | 4.49223948 |
| MT239365 | 41.87 | 45.12 | 53.66 | 26.83 | 51.756 | *psaC* | 50.01516 | -1.740839759 |
| MT239365 | 41.87 | 45.12 | 53.66 | 26.83 | 51.756 | *psaC* | 50.01516 | -1.740839759 |
| MT239365 | 32.43 | 40.54 | 29.73 | 27.03 | 41.643 | *psaI* | 50.16269 | 8.519689698 |
| MT239365 | 32.43 | 40.54 | 29.73 | 27.03 | 41.643 | *psaI* | 50.16269 | 8.519689698 |
| MT239365 | 37.59 | 40.43 | 42.55 | 29.79 | 39.036 | *psaJ* | 52.15273 | 13.11673476 |
| MT239365 | 41.53 | 49.44 | 43.79 | 31.36 | 41.134 | *psbA* | 53.23636 | 12.1023626 |
| MT239365 | 44.2 | 54.81 | 45.97 | 31.83 | 49.328 | *psbB* | 53.55235 | 4.224353445 |
| MT239365 | 43.95 | 53.59 | 46.41 | 31.86 | 44.819 | *psbC* | 53.57238 | 8.753380585 |
| MT239365 | 42.47 | 51.98 | 43.22 | 32.2 | 46.111 | *psbD* | 53.79812 | 7.687122179 |
| MT239365 | 41.67 | 44.05 | 47.62 | 33.33 | 50.06 | *psbE* | 54.53121 | 4.471211875 |
| MT239365 | 39.17 | 47.5 | 45 | 25 | 44 | *psbF* | 48.65 | 4.65 |
| MT239365 | 37.84 | 37.84 | 47.3 | 28.38 | 41.647 | *psbH* | 51.14772 | 9.50072388 |
| MT239365 | 37.84 | 54.05 | 32.43 | 27.03 | 27.272 | *psbI* | 50.16269 | 22.8906897 |
| MT239365 | 41.46 | 46.34 | 48.78 | 29.27 | 35.964 | *psbJ* | 51.78527 | 15.82126637 |
| MT239365 | 35.48 | 37.1 | 33.87 | 35.48 | 51.793 | *psbK* | 55.84394 | 4.050944876 |
| MT239365 | 32.48 | 35.9 | 25.64 | 35.9 | 52.793 | *psbL* | 56.08638 | 3.293383551 |
| MT239365 | 29.52 | 45.71 | 25.71 | 17.14 | 47 | *psbM* | 42.67669 | -4.323313026 |
| MT239365 | 42.42 | 54.55 | 40.91 | 31.82 | 46.02 | *psbN* | 53.54567 | 7.525673867 |
| MT239365 | 34.26 | 38.89 | 30.56 | 33.33 | 56.893 | *psbT* | 54.53121 | -2.361788125 |
| MT239365 | 35.98 | 41.27 | 41.27 | 25.4 | 44.431 | *psbZ* | 48.95047 | 4.519466527 |
| MT239365 | 43.49 | 57.98 | 43.49 | 28.99 | 47.083 | *rbcL* | 51.58582 | 4.502816622 |
| MT239365 | 42.16 | 47.76 | 48.51 | 30.22 | 51.135 | *rpl2* | 52.45355 | 1.318546387 |
| MT239365 | 38.1 | 53.38 | 34.59 | 26.32 | 52.153 | *rpl14* | 49.63733 | -2.515672729 |
| MT239365 | 39.9 | 47.45 | 51.82 | 20.44 | 37.45 | *rpl16* | 45.18273 | 7.732725645 |
| MT239365 | 38.3 | 37.72 | 48.25 | 28.95 | 43.529 | *rpl20* | 51.55724 | 8.028237022 |
| MT239365 | 34.84 | 40.65 | 39.35 | 24.52 | 41.993 | *rpl22* | 48.2882 | 6.295195139 |
| MT239365 | 32.99 | 40.62 | 33.33 | 25 | 49.603 | *rpl23* | 48.65 | -0.953 |
| MT239365 | 35.78 | 35.29 | 48.53 | 23.53 | 47.675 | *rpl32* | 47.53844 | -0.136556515 |
| MT239365 | 35.78 | 35.29 | 48.53 | 23.53 | 47.675 | *rpl32* | 47.53844 | -0.136556515 |
| MT239365 | 37.44 | 39.73 | 35.62 | 36.99 | 53.171 | *rpl33* | 56.69207 | 3.521069653 |
| MT239365 | 36.84 | 39.47 | 47.37 | 23.68 | 36.35 | *rpl36* | 47.6523 | 11.30230236 |
| MT239365 | 34.79 | 43.27 | 36 | 25.09 | 45.563 | *rpoA* | 48.71769 | 3.154691738 |
| MT239365 | 38.21 | 48.98 | 36.96 | 28.68 | 48.431 | *rpoB* | 51.36377 | 2.932773815 |
| MT239365 | 37.95 | 49.42 | 36.88 | 27.55 | 48.343 | *rpoC1* | 50.54443 | 2.20143453 |
| MT239365 | 36.98 | 45.66 | 36.69 | 28.59 | 49.658 | *rpoC2* | 51.29908 | 1.641077142 |
| MT239365 | 37.29 | 41.53 | 40.68 | 29.66 | 50.833 | *rps2* | 52.06124 | 1.22823729 |
| MT239365 | 34.44 | 43.19 | 36.54 | 23.59 | 48.688 | *rps3* | 47.584 | -1.104003161 |
| MT239365 | 37.44 | 46.92 | 38.86 | 26.54 | 48.335 | *rps4* | 49.80059 | 1.465592106 |
| MT239365 | 39.74 | 51.28 | 42.31 | 25.64 | 46.54 | *rps7* | 49.13024 | 2.590244317 |
| MT239365 | 34.09 | 40.6 | 39.85 | 21.8 | 40.784 | *rps8* | 46.22086 | 5.436864738 |
| MT239365 | 41.49 | 48.92 | 53.96 | 21.58 | 51.001 | *rps11* | 46.05296 | -4.948044933 |
| MT239365 | 41.94 | 51.61 | 46.77 | 27.42 | 46.396 | *rps12* | 50.44926 | 4.053255793 |
| MT239365 | 41.91 | 45.54 | 46.53 | 33.66 | 45.876 | *rps14* | 54.74001 | 8.864008231 |
| MT239365 | 32.62 | 43.01 | 26.88 | 27.96 | 52.599 | *rps15* | 50.84342 | -1.755576284 |
| MT239365 | 32.62 | 43.01 | 26.88 | 27.96 | 52.599 | *rps15* | 50.84342 | -1.755576284 |
| MT239365 | 34.47 | 51.14 | 31.82 | 20.45 | 49.795 | *rps16* | 45.19036 | -4.604643088 |
| MT239365 | 34.47 | 51.14 | 31.82 | 20.45 | 49.795 | *rps16* | 45.19036 | -4.604643088 |
| MT239365 | 30.77 | 27.69 | 41.54 | 23.08 | 42.026 | *rps18* | 47.19641 | 5.17041278 |
| MT239365 | 34.05 | 43.01 | 37.63 | 21.51 | 52.166 | *rps19* | 45.99952 | -6.166477706 |
| MT239365 | 38.46 | 47.93 | 38.46 | 28.99 | 51.943 | *ycf3* | 51.58582 | -0.357183378 |
| MT239365 | 38.92 | 48.11 | 40 | 28.65 | 50.179 | *ycf4* | 51.34222 | 1.163219685 |
| MT239365 | 38.92 | 48.11 | 40 | 28.65 | 50.179 | *ycf4* | 51.34222 | 1.163219685 |
| MT239366 | 40.03 | 54.33 | 40.16 | 25.59 | 44.792 | *atpA* | 49.09282 | 4.300823297 |
| MT239366 | 41.82 | 56.31 | 41.68 | 27.45 | 46.122 | *atpB* | 50.47124 | 4.349235751 |
| MT239366 | 38.56 | 49.25 | 38.06 | 28.36 | 46.088 | *atpE* | 51.13328 | 5.045280975 |
| MT239366 | 38.02 | 45.95 | 34.59 | 33.51 | 45.383 | *atpF* | 54.64541 | 9.262410099 |
| MT239366 | 45.12 | 60.98 | 48.78 | 25.61 | 49.405 | *atpH* | 49.10779 | -0.297206193 |
| MT239366 | 38.84 | 49.19 | 37.1 | 30.24 | 46.118 | *atpI* | 52.46747 | 6.349467289 |
| MT239366 | 33.33 | 35.51 | 37.07 | 27.41 | 47.843 | *ccsA* | 50.44193 | 2.598927074 |
| MT239366 | 33.33 | 35.51 | 37.07 | 27.41 | 47.843 | *ccsA* | 50.44193 | 2.598927074 |
| MT239366 | 33.05 | 38.79 | 28.02 | 32.33 | 50.216 | *cemA* | 53.88383 | 3.667825532 |
| MT239366 | 33.05 | 38.79 | 28.02 | 32.33 | 50.216 | *cemA* | 53.88383 | 3.667825532 |
| MT239366 | 32.68 | 38.66 | 31.56 | 27.81 | 47.77 | *matK* | 50.73425 | 2.964251911 |
| MT239366 | 34.25 | 41.1 | 39.73 | 21.92 | 42.082 | *ndhA* | 46.31243 | 4.230433896 |
| MT239366 | 34.25 | 41.1 | 39.73 | 21.92 | 42.082 | *ndhA* | 46.31243 | 4.230433896 |
| MT239366 | 36.99 | 41.49 | 37.57 | 31.9 | 47.103 | *ndhB* | 53.59906 | 6.496056302 |
| MT239366 | 35.26 | 47.11 | 31.4 | 27.27 | 54.128 | *ndhC* | 50.33922 | -3.788782304 |
| MT239366 | 35.44 | 40.43 | 37.48 | 28.4 | 45.24 | *ndhD* | 51.16216 | 5.922161911 |
| MT239366 | 35.44 | 40.43 | 37.48 | 28.4 | 45.24 | *ndhD* | 51.16216 | 5.922161911 |
| MT239366 | 33.01 | 40.2 | 34.31 | 24.51 | 39.896 | *ndhE* | 48.28064 | 8.38464433 |
| MT239366 | 33.01 | 40.2 | 34.31 | 24.51 | 39.896 | *ndhE* | 48.28064 | 8.38464433 |
| MT239366 | 32.09 | 35.96 | 35.7 | 24.6 | 43.477 | *ndhF* | 48.34858 | 4.871583016 |
| MT239366 | 32.58 | 40.68 | 33.9 | 23.16 | 47.503 | *ndhG* | 47.25726 | -0.245735993 |
| MT239366 | 32.58 | 40.68 | 33.9 | 23.16 | 47.503 | *ndhG* | 47.25726 | -0.245735993 |
| MT239366 | 37.65 | 51.02 | 36.55 | 25.38 | 49.275 | *ndhH* | 48.93547 | -0.339532528 |
| MT239366 | 37.65 | 51.02 | 36.55 | 25.38 | 49.275 | *ndhH* | 48.93547 | -0.339532528 |
| MT239366 | 35.77 | 42.7 | 36.52 | 28.09 | 48.067 | *ndhI* | 50.93783 | 2.870832752 |
| MT239366 | 35.77 | 42.7 | 36.52 | 28.09 | 48.067 | *ndhI* | 50.93783 | 2.870832752 |
| MT239366 | 38.57 | 50.31 | 35.85 | 29.56 | 46.766 | *ndhJ* | 51.99068 | 5.224684669 |
| MT239366 | 37.17 | 43.81 | 42.48 | 25.22 | 49.166 | *ndhK* | 48.81538 | -0.350618773 |
| MT239366 | 40.29 | 52.02 | 36.45 | 32.4 | 50.157 | *petA* | 53.92983 | 3.772831103 |
| MT239366 | 40.29 | 52.02 | 36.45 | 32.4 | 50.157 | *petA* | 53.92983 | 3.772831103 |
| MT239366 | 39.66 | 48.61 | 42.13 | 28.24 | 44.435 | *petB* | 51.04652 | 6.61152209 |
| MT239366 | 37.27 | 50.31 | 39.13 | 22.36 | 45.147 | *petD* | 46.64803 | 1.501033365 |
| MT239366 | 34.21 | 50 | 28.95 | 23.68 | 43.133 | *petG* | 47.6523 | 4.519302359 |
| MT239366 | 32.29 | 31.25 | 43.75 | 21.88 | 55.5 | *petL* | 46.28191 | -9.218087523 |
| MT239366 | 41.11 | 50 | 43.33 | 30 | 30.698 | *petN* | 52.3 | 21.602 |
| MT239366 | 42.7 | 52.06 | 43.14 | 32.89 | 50.61 | *psaA* | 54.24901 | 3.639011348 |
| MT239366 | 40.91 | 48.3 | 43.13 | 31.29 | 48.706 | *psaB* | 53.18895 | 4.482948718 |
| MT239366 | 41.87 | 45.12 | 53.66 | 26.83 | 51.756 | *psaC* | 50.01516 | -1.740839759 |
| MT239366 | 41.87 | 45.12 | 53.66 | 26.83 | 51.756 | *psaC* | 50.01516 | -1.740839759 |
| MT239366 | 32.43 | 40.54 | 29.73 | 27.03 | 41.643 | *psaI* | 50.16269 | 8.519689698 |
| MT239366 | 32.43 | 40.54 | 29.73 | 27.03 | 41.643 | *psaI* | 50.16269 | 8.519689698 |
| MT239366 | 37.59 | 40.43 | 42.55 | 29.79 | 39.036 | *psaJ* | 52.15273 | 13.11673476 |
| MT239366 | 41.34 | 49.15 | 43.79 | 31.07 | 40.69 | *psbA* | 53.03936 | 12.34935769 |
| MT239366 | 44.14 | 54.62 | 45.97 | 31.83 | 48.994 | *psbB* | 53.55235 | 4.558353445 |
| MT239366 | 43.95 | 53.59 | 46.41 | 31.86 | 44.857 | *psbC* | 53.57238 | 8.715380585 |
| MT239366 | 42.66 | 51.98 | 43.22 | 32.77 | 46.551 | *psbD* | 54.17131 | 7.620314345 |
| MT239366 | 41.67 | 44.05 | 47.62 | 33.33 | 50.06 | *psbE* | 54.53121 | 4.471211875 |
| MT239366 | 39.17 | 47.5 | 45 | 25 | 44 | *psbF* | 48.65 | 4.65 |
| MT239366 | 37.84 | 37.84 | 47.3 | 28.38 | 41.647 | *psbH* | 51.14772 | 9.50072388 |
| MT239366 | 37.84 | 54.05 | 32.43 | 27.03 | 27.272 | *psbI* | 50.16269 | 22.8906897 |
| MT239366 | 41.46 | 46.34 | 48.78 | 29.27 | 35.964 | *psbJ* | 51.78527 | 15.82126637 |
| MT239366 | 35.48 | 38.71 | 33.87 | 33.87 | 50.008 | *psbK* | 54.87156 | 4.863564832 |
| MT239366 | 32.48 | 35.9 | 25.64 | 35.9 | 52.793 | *psbL* | 56.08638 | 3.293383551 |
| MT239366 | 29.52 | 45.71 | 25.71 | 17.14 | 47 | *psbM* | 42.67669 | -4.323313026 |
| MT239366 | 42.42 | 54.55 | 40.91 | 31.82 | 46.02 | *psbN* | 53.54567 | 7.525673867 |
| MT239366 | 34.26 | 38.89 | 30.56 | 33.33 | 56.893 | *psbT* | 54.53121 | -2.361788125 |
| MT239366 | 35.98 | 41.27 | 41.27 | 25.4 | 44.431 | *psbZ* | 48.95047 | 4.519466527 |
| MT239366 | 43.63 | 57.98 | 43.7 | 29.2 | 47.439 | *rbcL* | 51.7355 | 4.296504828 |
| MT239366 | 42.29 | 48.13 | 48.51 | 30.22 | 51.518 | *rpl2* | 52.45355 | 0.935546387 |
| MT239366 | 37.59 | 52.63 | 34.59 | 25.56 | 52.831 | *rpl14* | 49.07036 | -3.760637685 |
| MT239366 | 40.15 | 47.45 | 52.55 | 20.44 | 37.282 | *rpl16* | 45.18273 | 7.900725645 |
| MT239366 | 38.3 | 37.72 | 48.25 | 28.95 | 43.529 | *rpl20* | 51.55724 | 8.028237022 |
| MT239366 | 34.41 | 39.35 | 39.35 | 24.52 | 42.675 | *rpl22* | 48.2882 | 5.613195139 |
| MT239366 | 32.99 | 40.62 | 33.33 | 25 | 49.603 | *rpl23* | 48.65 | -0.953 |
| MT239366 | 35.78 | 35.29 | 48.53 | 23.53 | 47.675 | *rpl32* | 47.53844 | -0.136556515 |
| MT239366 | 35.78 | 35.29 | 48.53 | 23.53 | 47.675 | *rpl32* | 47.53844 | -0.136556515 |
| MT239366 | 36.99 | 38.36 | 35.62 | 36.99 | 50.543 | *rpl33* | 56.69207 | 6.149069653 |
| MT239366 | 36.84 | 39.47 | 47.37 | 23.68 | 36.35 | *rpl36* | 47.6523 | 11.30230236 |
| MT239366 | 34.68 | 42.28 | 36.4 | 25.37 | 45.554 | *rpoA* | 48.92797 | 3.373966954 |
| MT239366 | 38.05 | 48.88 | 36.78 | 28.49 | 48.641 | *rpoB* | 51.22707 | 2.586072326 |
| MT239366 | 37.9 | 49.42 | 36.88 | 27.41 | 48.287 | *rpoC1* | 50.44193 | 2.154927074 |
| MT239366 | 37.08 | 45.81 | 36.76 | 28.67 | 49.702 | *rpoC2* | 51.35659 | 1.65459038 |
| MT239366 | 37.43 | 41.95 | 40.68 | 29.66 | 50.439 | *rps2* | 52.06124 | 1.62223729 |
| MT239366 | 34.87 | 44.96 | 34.03 | 25.63 | 48.837 | *rps3* | 49.12276 | 0.285761517 |
| MT239366 | 36.84 | 45.93 | 38.28 | 26.32 | 46.617 | *rps4* | 49.63733 | 3.020327271 |
| MT239366 | 39.74 | 51.28 | 42.31 | 25.64 | 50.07 | *rps7* | 49.13024 | -0.939755683 |
| MT239366 | 34.09 | 39.85 | 39.85 | 22.56 | 44.946 | *rps8* | 46.80047 | 1.854474969 |
| MT239366 | 41.49 | 48.92 | 53.96 | 21.58 | 51.001 | *rps11* | 46.05296 | -4.948044933 |
| MT239366 | 42.74 | 47.58 | 48.39 | 32.26 | 49.966 | *rps12* | 53.83772 | 3.871720079 |
| MT239366 | 42.24 | 46.53 | 46.53 | 33.66 | 45.74 | *rps14* | 54.74001 | 9.000008231 |
| MT239366 | 32.62 | 43.01 | 26.88 | 27.96 | 52.599 | *rps15* | 50.84342 | -1.755576284 |
| MT239366 | 32.62 | 43.01 | 26.88 | 27.96 | 52.599 | *rps15* | 50.84342 | -1.755576284 |
| MT239366 | 34.47 | 51.14 | 31.82 | 20.45 | 49.795 | *rps16* | 45.19036 | -4.604643088 |
| MT239366 | 34.47 | 51.14 | 31.82 | 20.45 | 49.795 | *rps16* | 45.19036 | -4.604643088 |
| MT239366 | 33.03 | 33.33 | 41.44 | 24.32 | 37.752 | *rps18* | 48.13708 | 10.38508372 |
| MT239366 | 33.69 | 43.01 | 36.56 | 21.51 | 54.586 | *rps19* | 45.99952 | -8.586477706 |
| MT239366 | 38.52 | 45.51 | 37.72 | 32.34 | 54.592 | *ycf3* | 53.8904 | -0.701596111 |
| MT239366 | 38.92 | 48.11 | 40 | 28.65 | 50.179 | *ycf4* | 51.34222 | 1.163219685 |
| MT239366 | 38.92 | 48.11 | 40 | 28.65 | 50.179 | *ycf4* | 51.34222 | 1.163219685 |
| MT985162 | 38.69 | 54.06 | 30.57 | 31.45 | 49.184 | *accD* | 53.29719 | 4.113191376 |
| MT985162 | 40.03 | 54.13 | 40.16 | 25.79 | 44.765 | *atpA* | 49.2424 | 4.47740072 |
| MT985162 | 41.82 | 56.11 | 41.68 | 27.66 | 46.317 | *atpB* | 50.62483 | 4.307831035 |
| MT985162 | 38.31 | 49.25 | 38.06 | 27.61 | 47.054 | *atpE* | 50.5883 | 3.534303186 |
| MT985162 | 38.02 | 45.95 | 34.59 | 33.51 | 45.492 | *atpF* | 54.64541 | 9.153410099 |
| MT985162 | 45.93 | 60.98 | 48.78 | 28.05 | 51.174 | *atpH* | 50.9088 | -0.265195476 |
| MT985162 | 38.58 | 49.19 | 37.1 | 29.44 | 46.049 | *atpI* | 51.90583 | 5.856829597 |
| MT985162 | 33.33 | 35.51 | 37.07 | 27.41 | 47.843 | *ccsA* | 50.44193 | 2.598927074 |
| MT985162 | 33.33 | 35.51 | 37.07 | 27.41 | 47.843 | *ccsA* | 50.44193 | 2.598927074 |
| MT985162 | 33.33 | 39.13 | 28.26 | 32.61 | 46.447 | *cemA* | 54.06724 | 7.620241141 |
| MT985162 | 33.33 | 39.13 | 28.26 | 32.61 | 46.447 | *cemA* | 54.06724 | 7.620241141 |
| MT985162 | 36.99 | 47.56 | 37.8 | 25.61 | 54.141 | *infA* | 49.10779 | -5.033206193 |
| MT985162 | 32.61 | 39.05 | 31.56 | 27.22 | 47.256 | *matK* | 50.30249 | 3.046487833 |
| MT985162 | 34.16 | 40.93 | 39.56 | 21.98 | 41.646 | *ndhA* | 46.35821 | 4.712212705 |
| MT985162 | 34.16 | 40.93 | 39.56 | 21.98 | 41.646 | *ndhA* | 46.35821 | 4.712212705 |
| MT985162 | 37.12 | 41.38 | 38.13 | 31.85 | 46.595 | *ndhB* | 53.56571 | 6.970706806 |
| MT985162 | 34.99 | 47.11 | 31.4 | 26.45 | 56.087 | *ndhC* | 49.73385 | -6.353148423 |
| MT985162 | 35.44 | 40.43 | 37.48 | 28.4 | 45.366 | *ndhD* | 51.16216 | 5.796161911 |
| MT985162 | 35.4 | 40.32 | 37.72 | 28.14 | 44.95 | *ndhD* | 50.97409 | 6.024091892 |
| MT985162 | 33.33 | 40.59 | 34.65 | 24.75 | 39.896 | *ndhE* | 48.46172 | 8.56571884 |
| MT985162 | 33.33 | 40.59 | 34.65 | 24.75 | 39.896 | *ndhE* | 48.46172 | 8.56571884 |
| MT985162 | 32.09 | 35.96 | 35.83 | 24.47 | 42.541 | *ndhF* | 48.25044 | 5.709435995 |
| MT985162 | 32.77 | 41.57 | 33.71 | 23.03 | 47.492 | *ndhG* | 47.15837 | -0.333628448 |
| MT985162 | 32.77 | 41.57 | 33.71 | 23.03 | 47.492 | *ndhG* | 47.15837 | -0.333628448 |
| MT985162 | 37.65 | 51.02 | 36.55 | 25.38 | 49.275 | *ndhH* | 48.93547 | -0.339532528 |
| MT985162 | 37.65 | 51.02 | 36.55 | 25.38 | 49.275 | *ndhH* | 48.93547 | -0.339532528 |
| MT985162 | 35.77 | 42.7 | 36.52 | 28.09 | 48.067 | *ndhI* | 50.93783 | 2.870832752 |
| MT985162 | 35.77 | 42.7 | 36.52 | 28.09 | 48.067 | *ndhI* | 50.93783 | 2.870832752 |
| MT985162 | 38.78 | 50.31 | 37.11 | 28.93 | 45.032 | *ndhJ* | 51.54294 | 6.510939224 |
| MT985162 | 37.32 | 43.81 | 42.48 | 25.66 | 51.108 | *ndhK* | 49.14521 | -1.962792198 |
| MT985162 | 40.19 | 52.02 | 36.45 | 32.09 | 49.848 | *petA* | 53.72534 | 3.877338387 |
| MT985162 | 40.19 | 52.02 | 36.45 | 32.09 | 49.848 | *petA* | 53.72534 | 3.877338387 |
| MT985162 | 39.51 | 48.61 | 42.13 | 27.78 | 44.342 | *petB* | 50.71239 | 6.370387453 |
| MT985162 | 37.68 | 50.93 | 39.13 | 22.98 | 45.022 | *petD* | 47.12032 | 2.098323468 |
| MT985162 | 34.21 | 50 | 28.95 | 23.68 | 43.133 | *petG* | 47.6523 | 4.519302359 |
| MT985162 | 32.29 | 31.25 | 43.75 | 21.88 | 55.5 | *petL* | 46.28191 | -9.218087523 |
| MT985162 | 41.11 | 50 | 43.33 | 30 | 30.698 | *petN* | 52.3 | 21.602 |
| MT985162 | 42.7 | 52.2 | 43.14 | 32.76 | 50.489 | *psaA* | 54.16483 | 3.675825677 |
| MT985162 | 40.82 | 48.44 | 43.13 | 30.88 | 48.181 | *psaB* | 52.90948 | 4.728475383 |
| MT985162 | 41.87 | 45.12 | 53.66 | 26.83 | 51.756 | *psaC* | 50.01516 | -1.740839759 |
| MT985162 | 41.87 | 45.12 | 53.66 | 26.83 | 51.756 | *psaC* | 50.01516 | -1.740839759 |
| MT985162 | 32.43 | 40.54 | 29.73 | 27.03 | 41.643 | *psaI* | 50.16269 | 8.519689698 |
| MT985162 | 32.43 | 40.54 | 29.73 | 27.03 | 41.643 | *psaI* | 50.16269 | 8.519689698 |
| MT985162 | 37.59 | 40.43 | 42.55 | 29.79 | 39.036 | *psaJ* | 52.15273 | 13.11673476 |
| MT985162 | 41.43 | 49.44 | 43.79 | 31.07 | 41.524 | *psbA* | 53.03936 | 11.51535769 |
| MT985162 | 44.01 | 54.62 | 45.97 | 31.43 | 48.539 | *psbB* | 53.28369 | 4.744686767 |
| MT985162 | 44.09 | 54.01 | 46.41 | 31.86 | 45.106 | *psbC* | 53.57238 | 8.466380585 |
| MT985162 | 42.37 | 51.98 | 43.22 | 31.92 | 45.802 | *psbD* | 53.61238 | 7.810382492 |
| MT985162 | 41.67 | 44.05 | 47.62 | 33.33 | 50.06 | *psbE* | 54.53121 | 4.471211875 |
| MT985162 | 39.17 | 47.5 | 45 | 25 | 44 | *psbF* | 48.65 | 4.65 |
| MT985162 | 37.39 | 36.49 | 47.3 | 28.38 | 42.805 | *psbH* | 51.14772 | 8.34272388 |
| MT985162 | 37.84 | 54.05 | 32.43 | 27.03 | 27.272 | *psbI* | 50.16269 | 22.8906897 |
| MT985162 | 41.46 | 46.34 | 48.78 | 29.27 | 35.964 | *psbJ* | 51.78527 | 15.82126637 |
| MT985162 | 36.02 | 38.71 | 33.87 | 35.48 | 54.824 | *psbK* | 55.84394 | 1.019944876 |
| MT985162 | 32.48 | 35.9 | 25.64 | 35.9 | 52.793 | *psbL* | 56.08638 | 3.293383551 |
| MT985162 | 30.77 | 42.31 | 25 | 25 | 59.686 | *psbM* | 48.65 | -11.036 |
| MT985162 | 42.42 | 54.55 | 40.91 | 31.82 | 46.02 | *psbN* | 53.54567 | 7.525673867 |
| MT985162 | 34.26 | 38.89 | 30.56 | 33.33 | 56.893 | *psbT* | 54.53121 | -2.361788125 |
| MT985162 | 36.51 | 41.27 | 41.27 | 26.98 | 46.925 | *psbZ* | 50.12584 | 3.200842687 |
| MT985162 | 43.56 | 58.61 | 43.07 | 28.99 | 46.912 | *rbcL* | 51.58582 | 4.673816622 |
| MT985162 | 42.16 | 48.51 | 48.51 | 29.48 | 49.344 | *rpl2* | 51.93414 | 2.590137689 |
| MT985162 | 39.39 | 56.06 | 34.85 | 27.27 | 50.124 | *rpl14* | 50.33922 | 0.215217696 |
| MT985162 | 39.95 | 47.79 | 52.21 | 19.85 | 36.588 | *rpl16* | 44.73269 | 8.144686853 |
| MT985162 | 38.3 | 37.72 | 48.25 | 28.95 | 43.529 | *rpl20* | 51.55724 | 8.028237022 |
| MT985162 | 34.41 | 39.35 | 39.35 | 24.52 | 42.675 | *rpl22* | 48.2882 | 5.613195139 |
| MT985162 | 32.62 | 40.86 | 34.41 | 22.58 | 48.969 | *rpl23* | 46.81571 | -2.153285144 |
| MT985162 | 35.78 | 35.29 | 48.53 | 23.53 | 47.675 | *rpl32* | 47.53844 | -0.136556515 |
| MT985162 | 35.78 | 35.29 | 48.53 | 23.53 | 47.675 | *rpl32* | 47.53844 | -0.136556515 |
| MT985162 | 36.99 | 36.99 | 35.62 | 38.36 | 59.955 | *rpl33* | 57.40184 | -2.553159746 |
| MT985162 | 36.84 | 36.84 | 50 | 23.68 | 26.489 | *rpl36* | 47.6523 | 21.16330236 |
| MT985162 | 34.53 | 43.17 | 35.61 | 24.82 | 44.848 | *rpoA* | 48.51447 | 3.666473444 |
| MT985162 | 38.1 | 48.79 | 36.82 | 28.69 | 48.642 | *rpoB* | 51.37096 | 2.728955972 |
| MT985162 | 37.79 | 49.42 | 36.48 | 27.47 | 48.476 | *rpoC1* | 50.48588 | 2.009883871 |
| MT985162 | 36.83 | 45.3 | 36.62 | 28.59 | 49.62 | *rpoC2* | 51.29908 | 1.679077142 |
| MT985162 | 37.29 | 41.95 | 40.25 | 29.66 | 50.105 | *rps2* | 52.06124 | 1.95623729 |
| MT985162 | 35.15 | 44.77 | 34.31 | 26.36 | 46.493 | *rps3* | 49.66704 | 3.174042131 |
| MT985162 | 37.16 | 46.89 | 38.76 | 25.84 | 48.416 | *rps4* | 49.27975 | 0.863749898 |
| MT985162 | 39.53 | 51.28 | 42.31 | 25 | 46.355 | *rps7* | 48.65 | 2.295 |
| MT985162 | 33.58 | 39.85 | 39.85 | 21.05 | 40.338 | *rps8* | 45.64835 | 5.310351294 |
| MT985162 | 41.49 | 48.92 | 53.96 | 21.58 | 51.001 | *rps11* | 46.05296 | -4.948044933 |
| MT985162 | 42.2 | 50.81 | 46.77 | 29.03 | 44.647 | *rps12* | 51.61437 | 6.967374785 |
| MT985162 | 42.9 | 47.52 | 47.52 | 33.66 | 43.475 | *rps14* | 54.74001 | 11.26500823 |
| MT985162 | 31.9 | 43.01 | 24.73 | 27.96 | 55.317 | *rps15* | 50.84342 | -4.473576284 |
| MT985162 | 31.9 | 43.01 | 24.73 | 27.96 | 55.317 | *rps15* | 50.84342 | -4.473576284 |
| MT985162 | 34.47 | 51.14 | 31.82 | 20.45 | 50.19 | *rps16* | 45.19036 | -4.999643088 |
| MT985162 | 34.47 | 51.14 | 31.82 | 20.45 | 50.19 | *rps16* | 45.19036 | -4.999643088 |
| MT985162 | 33.04 | 32.74 | 40.71 | 25.66 | 38.032 | *rps18* | 49.14521 | 11.1132078 |
| MT985162 | 33.69 | 43.01 | 36.56 | 21.51 | 54.586 | *rps19* | 45.99952 | -8.586477706 |
| MT985162 | 36.29 | 37.5 | 32.26 | 39.11 | 53.852 | *ycf1* | 57.76437 | 3.912367184 |
| MT985162 | 36.29 | 37.5 | 32.26 | 39.11 | 53.852 | *ycf1* | 57.76437 | 3.912367184 |
| MT985162 | 38.26 | 47.93 | 38.46 | 28.4 | 51.379 | *ycf3* | 51.16216 | -0.216838089 |
| MT985162 | 38.92 | 48.11 | 40 | 28.65 | 50.179 | *ycf4* | 51.34222 | 1.163219685 |
| MT985162 | 38.92 | 48.11 | 40 | 28.65 | 50.179 | *ycf4* | 51.34222 | 1.163219685 |
| MZ073672 | 39.23 | 55.51 | 29.85 | 32.32 | 50.885 | *accD* | 53.87725 | 2.992245137 |
| MZ073672 | 39.9 | 54.13 | 40.16 | 25.39 | 44.196 | *atpA* | 48.94297 | 4.74696733 |
| MZ073672 | 41.82 | 56.31 | 41.48 | 27.66 | 46.309 | *atpB* | 50.62483 | 4.315831035 |
| MZ073672 | 38.81 | 49.25 | 38.81 | 28.36 | 45.944 | *atpE* | 51.13328 | 5.189280975 |
| MZ073672 | 37.66 | 45.41 | 34.59 | 32.97 | 44.75 | *atpF* | 54.30064 | 9.550636849 |
| MZ073672 | 45.53 | 60.98 | 48.78 | 26.83 | 50.468 | *atpH* | 50.01516 | -0.452839759 |
| MZ073672 | 38.84 | 49.19 | 37.1 | 30.24 | 46.118 | *atpI* | 52.46747 | 6.349467289 |
| MZ073672 | 33.33 | 35.51 | 37.38 | 27.1 | 47.881 | *ccsA* | 50.21424 | 2.33323521 |
| MZ073672 | 33.33 | 35.51 | 37.38 | 27.1 | 47.881 | *ccsA* | 50.21424 | 2.33323521 |
| MZ073672 | 33.48 | 39.57 | 28.26 | 32.61 | 49.604 | *cemA* | 54.06724 | 4.463241141 |
| MZ073672 | 33.48 | 39.57 | 28.26 | 32.61 | 49.604 | *cemA* | 54.06724 | 4.463241141 |
| MZ073672 | 36.59 | 47.56 | 37.8 | 24.39 | 51.463 | *infA* | 48.19 | -3.273004783 |
| MZ073672 | 32.74 | 39.05 | 31.76 | 27.42 | 47.526 | *matK* | 50.44926 | 2.923255793 |
| MZ073672 | 34.16 | 40.93 | 39.56 | 21.98 | 41.646 | *ndhA* | 46.35821 | 4.712212705 |
| MZ073672 | 34.16 | 40.93 | 39.56 | 21.98 | 41.646 | *ndhA* | 46.35821 | 4.712212705 |
| MZ073672 | 36.78 | 41.18 | 37.73 | 31.44 | 46.614 | *ndhB* | 53.29044 | 6.676439996 |
| MZ073672 | 34.71 | 46.28 | 31.4 | 26.45 | 56.563 | *ndhC* | 49.73385 | -6.829148423 |
| MZ073672 | 35.5 | 40.43 | 37.48 | 28.6 | 45.491 | *ndhD* | 51.30627 | 5.815270727 |
| MZ073672 | 35.5 | 40.43 | 37.48 | 28.6 | 45.491 | *ndhD* | 51.30627 | 5.815270727 |
| MZ073672 | 33.33 | 40.2 | 34.31 | 25.49 | 39.896 | *ndhE* | 49.01793 | 9.121929252 |
| MZ073672 | 33.33 | 40.2 | 34.31 | 25.49 | 39.896 | *ndhE* | 49.01793 | 9.121929252 |
| MZ073672 | 32.04 | 35.96 | 35.56 | 24.6 | 44.05 | *ndhF* | 48.34858 | 4.298583016 |
| MZ073672 | 32.04 | 35.96 | 35.56 | 24.6 | 44.05 | *ndhF* | 48.34858 | 4.298583016 |
| MZ073672 | 32.77 | 41.57 | 33.71 | 23.03 | 47.492 | *ndhG* | 47.15837 | -0.333628448 |
| MZ073672 | 32.77 | 41.57 | 33.71 | 23.03 | 47.492 | *ndhG* | 47.15837 | -0.333628448 |
| MZ073672 | 37.56 | 51.02 | 36.55 | 25.13 | 49.115 | *ndhH* | 48.74776 | -0.367238814 |
| MZ073672 | 37.56 | 51.02 | 36.55 | 25.13 | 49.115 | *ndhH* | 48.74776 | -0.367238814 |
| MZ073672 | 35.77 | 42.7 | 36.52 | 28.09 | 48.067 | *ndhI* | 50.93783 | 2.870832752 |
| MZ073672 | 35.77 | 42.7 | 36.52 | 28.09 | 48.067 | *ndhI* | 50.93783 | 2.870832752 |
| MZ073672 | 38.16 | 48.43 | 36.48 | 29.56 | 45.323 | *ndhJ* | 51.99068 | 6.667684669 |
| MZ073672 | 37.32 | 44.25 | 42.48 | 25.22 | 50.448 | *ndhK* | 48.81538 | -1.632618773 |
| MZ073672 | 39.98 | 52.02 | 36.45 | 31.46 | 49.689 | *petA* | 53.30394 | 3.614940907 |
| MZ073672 | 39.98 | 52.02 | 36.45 | 31.46 | 49.689 | *petA* | 53.30394 | 3.614940907 |
| MZ073672 | 39.81 | 48.61 | 42.13 | 28.7 | 44.409 | *petB* | 51.37814 | 6.969136849 |
| MZ073672 | 37.5 | 50.62 | 39.38 | 22.5 | 44.908 | *petD* | 46.75475 | 1.84675048 |
| MZ073672 | 34.21 | 50 | 28.95 | 23.68 | 43.133 | *petG* | 47.6523 | 4.519302359 |
| MZ073672 | 32.29 | 31.25 | 43.75 | 21.88 | 55.5 | *petL* | 46.28191 | -9.218087523 |
| MZ073672 | 41.11 | 50 | 43.33 | 30 | 30.698 | *petN* | 52.3 | 21.602 |
| MZ073672 | 42.61 | 52.2 | 43.14 | 32.49 | 50.335 | *psaA* | 53.98883 | 3.65383317 |
| MZ073672 | 40.77 | 48.3 | 43.13 | 30.88 | 48.015 | *psaB* | 52.90948 | 4.894475383 |
| MZ073672 | 41.87 | 45.12 | 53.66 | 26.83 | 51.756 | *psaC* | 50.01516 | -1.740839759 |
| MZ073672 | 41.87 | 45.12 | 53.66 | 26.83 | 51.756 | *psaC* | 50.01516 | -1.740839759 |
| MZ073672 | 32.43 | 40.54 | 29.73 | 27.03 | 41.643 | *psaI* | 50.16269 | 8.519689698 |
| MZ073672 | 32.43 | 40.54 | 29.73 | 27.03 | 41.643 | *psaI* | 50.16269 | 8.519689698 |
| MZ073672 | 36.88 | 38.3 | 42.55 | 29.79 | 38.418 | *psaJ* | 52.15273 | 13.73473476 |
| MZ073672 | 41.53 | 49.44 | 43.79 | 31.36 | 41.809 | *psbA* | 53.23636 | 11.4273626 |
| MZ073672 | 44.2 | 54.62 | 45.78 | 32.22 | 49.417 | *psbB* | 53.81133 | 4.394329544 |
| MZ073672 | 43.95 | 53.8 | 46.41 | 31.65 | 44.796 | *psbC* | 53.43183 | 8.635827636 |
| MZ073672 | 42.75 | 51.98 | 43.22 | 33.05 | 47.103 | *psbD* | 54.35212 | 7.249123057 |
| MZ073672 | 41.67 | 44.05 | 47.62 | 33.33 | 50.06 | *psbE* | 54.53121 | 4.471211875 |
| MZ073672 | 39.17 | 47.5 | 45 | 25 | 44 | *psbF* | 48.65 | 4.65 |
| MZ073672 | 37.84 | 37.84 | 47.3 | 28.38 | 41.647 | *psbH* | 51.14772 | 9.50072388 |
| MZ073672 | 37.84 | 54.05 | 32.43 | 27.03 | 27.272 | *psbI* | 50.16269 | 22.8906897 |
| MZ073672 | 41.46 | 46.34 | 48.78 | 29.27 | 35.964 | *psbJ* | 51.78527 | 15.82126637 |
| MZ073672 | 36.56 | 38.71 | 33.87 | 37.1 | 51.871 | *psbK* | 56.75123 | 4.880234098 |
| MZ073672 | 32.48 | 35.9 | 25.64 | 35.9 | 52.793 | *psbL* | 56.08638 | 3.293383551 |
| MZ073672 | 29.52 | 45.71 | 25.71 | 17.14 | 47 | *psbM* | 42.67669 | -4.323313026 |
| MZ073672 | 42.42 | 54.55 | 40.91 | 31.82 | 46.02 | *psbN* | 53.54567 | 7.525673867 |
| MZ073672 | 34.26 | 38.89 | 30.56 | 33.33 | 56.893 | *psbT* | 54.53121 | -2.361788125 |
| MZ073672 | 37.04 | 41.27 | 41.27 | 28.57 | 51.288 | *psbZ* | 51.28469 | -0.003313806 |
| MZ073672 | 43.7 | 58.4 | 43.07 | 29.62 | 47.63 | *rbcL* | 52.03303 | 4.403033837 |
| MZ073672 | 42.66 | 48.51 | 48.88 | 30.6 | 52.01 | *rpl2* | 52.71694 | 0.706936044 |
| MZ073672 | 39.39 | 55.3 | 35.61 | 27.27 | 51.503 | *rpl14* | 50.33922 | -1.163782304 |
| MZ073672 | 39.9 | 47.45 | 51.82 | 20.44 | 37.45 | *rpl16* | 45.18273 | 7.732725645 |
| MZ073672 | 38.01 | 38.6 | 47.37 | 28.07 | 41.667 | *rpl20* | 50.92332 | 9.256320953 |
| MZ073672 | 34.19 | 39.35 | 39.35 | 23.87 | 41.207 | *rpl22* | 47.7964 | 6.589401515 |
| MZ073672 | 32.29 | 41.67 | 32.29 | 22.92 | 48.815 | *rpl23* | 47.07466 | -1.740343012 |
| MZ073672 | 35.78 | 35.29 | 48.53 | 23.53 | 47.675 | *rpl32* | 47.53844 | -0.136556515 |
| MZ073672 | 35.78 | 35.29 | 48.53 | 23.53 | 47.675 | *rpl32* | 47.53844 | -0.136556515 |
| MZ073672 | 38.03 | 39.44 | 36.62 | 38.03 | 54.664 | *rpl33* | 57.23637 | 2.572369213 |
| MZ073672 | 37.72 | 39.47 | 50 | 23.68 | 31.625 | *rpl36* | 47.6523 | 16.02730236 |
| MZ073672 | 35.49 | 44.49 | 36.5 | 25.48 | 44.156 | *rpoA* | 49.01044 | 4.854436086 |
| MZ073672 | 38.18 | 48.83 | 36.9 | 28.83 | 48.741 | *rpoB* | 51.47137 | 2.730370889 |
| MZ073672 | 37.76 | 49.27 | 36.73 | 27.26 | 47.841 | *rpoC1* | 50.33187 | 2.490873723 |
| MZ073672 | 36.83 | 45.41 | 36.54 | 28.53 | 49.293 | *rpoC2* | 51.25589 | 1.962889244 |
| MZ073672 | 37.43 | 41.53 | 41.1 | 29.66 | 50.271 | *rps2* | 52.06124 | 1.79023729 |
| MZ073672 | 35.15 | 44.77 | 34.31 | 26.36 | 47.121 | *rps3* | 49.66704 | 2.546042131 |
| MZ073672 | 36.84 | 46.89 | 38.76 | 24.88 | 47.04 | *rps4* | 48.55967 | 1.51966973 |
| MZ073672 | 38.89 | 50 | 41.67 | 25 | 45.984 | *rps7* | 48.65 | 2.666 |
| MZ073672 | 33.33 | 38.35 | 39.85 | 21.8 | 40.455 | *rps8* | 46.22086 | 5.765864738 |
| MZ073672 | 41.49 | 48.92 | 53.96 | 21.58 | 50.163 | *rps11* | 46.05296 | -4.110044933 |
| MZ073672 | 42.74 | 50.81 | 48.39 | 29.03 | 48.506 | *rps12* | 51.61437 | 3.108374785 |
| MZ073672 | 42.9 | 47.52 | 47.52 | 33.66 | 43.475 | *rps14* | 54.74001 | 11.26500823 |
| MZ073672 | 31.9 | 43.01 | 25.81 | 26.88 | 51.34 | *rps15* | 50.05208 | -1.287922275 |
| MZ073672 | 31.9 | 43.01 | 25.81 | 26.88 | 51.34 | *rps15* | 50.05208 | -1.287922275 |
| MZ073672 | 34.47 | 51.14 | 31.82 | 20.45 | 49.795 | *rps16* | 45.19036 | -4.604643088 |
| MZ073672 | 34.47 | 51.14 | 31.82 | 20.45 | 49.795 | *rps16* | 45.19036 | -4.604643088 |
| MZ073672 | 33.06 | 33.33 | 41.67 | 24.17 | 37.385 | *rps18* | 48.02362 | 10.63862352 |
| MZ073672 | 40.14 | 46.24 | 43.01 | 31.18 | 48.859 | *ycf3* | 53.11426 | 4.25526167 |
| MZ073672 | 38.92 | 48.11 | 40 | 28.65 | 50.179 | *ycf4* | 51.34222 | 1.163219685 |
| MZ073672 | 38.92 | 48.11 | 40 | 28.65 | 50.179 | *ycf4* | 51.34222 | 1.163219685 |
| NC_047438 | 40.03 | 54.13 | 40.16 | 25.79 | 44.765 | *atpA* | 49.2424 | 4.47740072 |
| NC_047438 | 41.85 | 56.34 | 41.65 | 27.57 | 46.473 | *atpB* | 50.55906 | 4.086061663 |
| NC_047438 | 38.31 | 49.25 | 38.06 | 27.61 | 47.054 | *atpE* | 50.5883 | 3.534303186 |
| NC_047438 | 45.93 | 60.98 | 48.78 | 28.05 | 51.174 | *atpH* | 50.9088 | -0.265195476 |
| NC_047438 | 38.44 | 49.19 | 37.1 | 29.03 | 45.642 | *atpI* | 51.61437 | 5.972374785 |
| NC_047438 | 33.33 | 39.13 | 28.26 | 32.61 | 46.447 | *cemA* | 54.06724 | 7.620241141 |
| NC_047438 | 33.33 | 39.13 | 28.26 | 32.61 | 46.447 | *cemA* | 54.06724 | 7.620241141 |
| NC_047438 | 36.51 | 41.27 | 41.27 | 26.98 | 46.925 | *lhbA* | 50.12584 | 3.200842687 |
| NC_047438 | 32.54 | 38.86 | 31.56 | 27.22 | 47.189 | *matK* | 50.30249 | 3.113487833 |
| NC_047438 | 34.33 | 41.03 | 39.67 | 22.28 | 42.663 | *ndhA* | 46.58704 | 3.924036624 |
| NC_047438 | 34.33 | 41.03 | 39.67 | 22.28 | 42.663 | *ndhA* | 46.58704 | 3.924036624 |
| NC_047438 | 37.13 | 41.32 | 38.12 | 31.94 | 47.097 | *ndhB* | 53.6257 | 6.528700881 |
| NC_047438 | 34.99 | 47.11 | 31.4 | 26.45 | 56.087 | *ndhC* | 49.73385 | -6.353148423 |
| NC_047438 | 35.37 | 40.43 | 37.48 | 28.21 | 45.35 | *ndhD* | 51.02481 | 5.674805444 |
| NC_047438 | 35.37 | 40.43 | 37.48 | 28.21 | 45.35 | *ndhD* | 51.02481 | 5.674805444 |
| NC_047438 | 33.33 | 40.2 | 34.31 | 25.49 | 39.896 | *ndhE* | 49.01793 | 9.121929252 |
| NC_047438 | 33.33 | 40.2 | 34.31 | 25.49 | 39.896 | *ndhE* | 49.01793 | 9.121929252 |
| NC_047438 | 31.91 | 35.96 | 35.43 | 24.33 | 42.531 | *ndhF* | 48.14464 | 5.613643957 |
| NC_047438 | 37.65 | 51.02 | 36.55 | 25.38 | 49.275 | *ndhH* | 48.93547 | -0.339532528 |
| NC_047438 | 37.65 | 51.02 | 36.55 | 25.38 | 49.275 | *ndhH* | 48.93547 | -0.339532528 |
| NC_047438 | 35.77 | 42.7 | 36.52 | 28.09 | 48.067 | *ndhI* | 50.93783 | 2.870832752 |
| NC_047438 | 35.77 | 42.7 | 36.52 | 28.09 | 48.067 | *ndhI* | 50.93783 | 2.870832752 |
| NC_047438 | 38.78 | 50.31 | 37.11 | 28.93 | 45.032 | *ndhJ* | 51.54294 | 6.510939224 |
| NC_047438 | 40.19 | 52.02 | 36.45 | 32.09 | 49.848 | *petA* | 53.72534 | 3.877338387 |
| NC_047438 | 40.19 | 52.02 | 36.45 | 32.09 | 49.848 | *petA* | 53.72534 | 3.877338387 |
| NC_047438 | 39.51 | 48.61 | 42.13 | 27.78 | 44.342 | *petB* | 50.71239 | 6.370387453 |
| NC_047438 | 37.47 | 50.31 | 39.13 | 22.98 | 45.475 | *petD* | 47.12032 | 1.645323468 |
| NC_047438 | 34.21 | 50 | 28.95 | 23.68 | 43.133 | *petG* | 47.6523 | 4.519302359 |
| NC_047438 | 32.29 | 31.25 | 43.75 | 21.88 | 55.5 | *petL* | 46.28191 | -9.218087523 |
| NC_047438 | 40.62 | 50 | 40.62 | 31.25 | 30.698 | *petN* | 53.16182 | 22.46381507 |
| NC_047438 | 42.65 | 52.2 | 43.14 | 32.62 | 50.415 | *psaA* | 54.07376 | 3.658761548 |
| NC_047438 | 40.77 | 48.44 | 43.13 | 30.75 | 48.142 | *psaB* | 52.82025 | 4.678247937 |
| NC_047438 | 41.87 | 45.12 | 53.66 | 26.83 | 51.756 | *psaC* | 50.01516 | -1.740839759 |
| NC_047438 | 41.87 | 45.12 | 53.66 | 26.83 | 51.756 | *psaC* | 50.01516 | -1.740839759 |
| NC_047438 | 32.43 | 40.54 | 29.73 | 27.03 | 41.643 | *psaI* | 50.16269 | 8.519689698 |
| NC_047438 | 32.43 | 40.54 | 29.73 | 27.03 | 41.643 | *psaI* | 50.16269 | 8.519689698 |
| NC_047438 | 37.59 | 40.43 | 42.55 | 29.79 | 39.036 | *psaJ* | 52.15273 | 13.11673476 |
| NC_047438 | 41.43 | 49.44 | 43.79 | 31.07 | 41.524 | *psbA* | 53.03936 | 11.51535769 |
| NC_047438 | 44.01 | 54.62 | 45.97 | 31.43 | 48.539 | *psbB* | 53.28369 | 4.744686767 |
| NC_047438 | 44.35 | 54.13 | 46.28 | 32.64 | 46.25 | *psbC* | 54.0868 | 7.836796058 |
| NC_047438 | 42.47 | 51.98 | 43.22 | 32.2 | 46.148 | *psbD* | 53.79812 | 7.650122179 |
| NC_047438 | 41.67 | 44.05 | 47.62 | 33.33 | 50.06 | *psbE* | 54.53121 | 4.471211875 |
| NC_047438 | 39.17 | 47.5 | 45 | 25 | 44 | *psbF* | 48.65 | 4.65 |
| NC_047438 | 37.39 | 36.49 | 47.3 | 28.38 | 41.972 | *psbH* | 51.14772 | 9.17572388 |
| NC_047438 | 37.84 | 54.05 | 32.43 | 27.03 | 27.272 | *psbI* | 50.16269 | 22.8906897 |
| NC_047438 | 41.46 | 46.34 | 48.78 | 29.27 | 35.964 | *psbJ* | 51.78527 | 15.82126637 |
| NC_047438 | 36.02 | 38.71 | 33.87 | 35.48 | 54.824 | *psbK* | 55.84394 | 1.019944876 |
| NC_047438 | 32.48 | 35.9 | 25.64 | 35.9 | 52.793 | *psbL* | 56.08638 | 3.293383551 |
| NC_047438 | 29.52 | 45.71 | 25.71 | 17.14 | 47 | *psbM* | 42.67669 | -4.323313026 |
| NC_047438 | 42.42 | 54.55 | 40.91 | 31.82 | 46.02 | *psbN* | 53.54567 | 7.525673867 |
| NC_047438 | 34.19 | 38.46 | 30.77 | 33.33 | 61 | *psbT* | 54.53121 | -6.468788125 |
| NC_047438 | 43.77 | 58.61 | 43.49 | 29.2 | 47.033 | *rbcL* | 51.7355 | 4.702504828 |
| NC_047438 | 42.16 | 48.51 | 48.51 | 29.48 | 49.344 | *rpl2* | 51.93414 | 2.590137689 |
| NC_047438 | 39.14 | 55.3 | 34.85 | 27.27 | 50.175 | *rpl14* | 50.33922 | 0.164217696 |
| NC_047438 | 39.9 | 47.45 | 51.82 | 20.44 | 37.45 | *rpl16* | 45.18273 | 7.732725645 |
| NC_047438 | 34.41 | 39.35 | 39.35 | 24.52 | 42.675 | *rpl22* | 48.2882 | 5.613195139 |
| NC_047438 | 32.62 | 40.86 | 34.41 | 22.58 | 48.969 | *rpl23* | 46.81571 | -2.153285144 |
| NC_047438 | 35.78 | 35.29 | 48.53 | 23.53 | 47.675 | *rpl32* | 47.53844 | -0.136556515 |
| NC_047438 | 35.78 | 35.29 | 48.53 | 23.53 | 47.675 | *rpl32* | 47.53844 | -0.136556515 |
| NC_047438 | 36.99 | 38.36 | 35.62 | 36.99 | 50.543 | *rpl33* | 56.69207 | 6.149069653 |
| NC_047438 | 36.84 | 36.84 | 50 | 23.68 | 26.489 | *rpl36* | 47.6523 | 21.16330236 |
| NC_047438 | 34.41 | 43.17 | 35.25 | 24.82 | 45.162 | *rpoA* | 48.51447 | 3.352473444 |
| NC_047438 | 37.92 | 48.83 | 36.76 | 28.16 | 48.475 | *rpoB* | 50.98859 | 2.513587363 |
| NC_047438 | 37.74 | 49.56 | 36.34 | 27.33 | 48.366 | *rpoC1* | 50.38326 | 2.017260391 |
| NC_047438 | 36.89 | 45.4 | 36.79 | 28.48 | 49.543 | *rpoC2* | 51.21986 | 1.676864991 |
| NC_047438 | 37.29 | 41.95 | 40.25 | 29.66 | 50.105 | *rps2* | 52.06124 | 1.95623729 |
| NC_047438 | 34.47 | 46.02 | 32.39 | 25 | 48.388 | *rps3* | 48.65 | 0.262 |
| NC_047438 | 37 | 46.41 | 38.76 | 25.84 | 47.297 | *rps4* | 49.27975 | 1.982749898 |
| NC_047438 | 39.53 | 51.28 | 42.31 | 25 | 46.355 | *rps7* | 48.65 | 2.295 |
| NC_047438 | 33.83 | 39.85 | 39.85 | 21.8 | 40.338 | *rps8* | 46.22086 | 5.882864738 |
| NC_047438 | 41.25 | 48.92 | 53.96 | 20.86 | 50.419 | *rps11* | 45.5033 | -4.91569828 |
| NC_047438 | 42.2 | 50.81 | 46.77 | 29.03 | 44.647 | *rps12* | 51.61437 | 6.967374785 |
| NC_047438 | 42.9 | 47.52 | 47.52 | 33.66 | 43.475 | *rps14* | 54.74001 | 11.26500823 |
| NC_047438 | 31.9 | 43.01 | 24.73 | 27.96 | 55.317 | *rps15* | 50.84342 | -4.473576284 |
| NC_047438 | 31.9 | 43.01 | 24.73 | 27.96 | 55.317 | *rps15* | 50.84342 | -4.473576284 |
| NC_047438 | 33.04 | 33.04 | 40 | 26.09 | 37.851 | *rps18* | 49.46621 | 11.61521359 |
| NC_047438 | 33.69 | 43.01 | 36.56 | 21.51 | 54.586 | *rps19* | 45.99952 | -8.586477706 |
| NC_047438 | 38.82 | 48.24 | 38.82 | 29.41 | 52.262 | *ycf3* | 51.88458 | -0.377416503 |
| NC_047438 | 38.92 | 48.11 | 40 | 28.65 | 50.179 | *ycf4* | 51.34222 | 1.163219685 |
| NC_047438 | 38.92 | 48.11 | 40 | 28.65 | 50.179 | *ycf4* | 51.34222 | 1.163219685 |
| NC_050162 | 39.21 | 55.66 | 30.43 | 31.54 | 49.15 | *accD* | 53.35787 | 4.207870292 |
| NC_050162 | 40.03 | 54.13 | 40.16 | 25.79 | 44.765 | *atpA* | 49.2424 | 4.47740072 |
| NC_050162 | 41.82 | 56.11 | 41.68 | 27.66 | 46.317 | *atpB* | 50.62483 | 4.307831035 |
| NC_050162 | 38.31 | 49.25 | 38.06 | 27.61 | 47.054 | *atpE* | 50.5883 | 3.534303186 |
| NC_050162 | 38.2 | 46.49 | 34.59 | 33.51 | 45.429 | *atpF* | 54.64541 | 9.216410099 |
| NC_050162 | 45.93 | 60.98 | 48.78 | 28.05 | 51.174 | *atpH* | 50.9088 | -0.265195476 |
| NC_050162 | 38.58 | 49.19 | 37.1 | 29.44 | 46.049 | *atpI* | 51.90583 | 5.856829597 |
| NC_050162 | 33.33 | 35.51 | 37.07 | 27.41 | 47.843 | *ccsA* | 50.44193 | 2.598927074 |
| NC_050162 | 33.33 | 35.51 | 37.07 | 27.41 | 47.843 | *ccsA* | 50.44193 | 2.598927074 |
| NC_050162 | 33.33 | 39.13 | 28.26 | 32.61 | 46.447 | *cemA* | 54.06724 | 7.620241141 |
| NC_050162 | 33.33 | 39.13 | 28.26 | 32.61 | 46.447 | *cemA* | 54.06724 | 7.620241141 |
| NC_050162 | 36.99 | 47.56 | 37.8 | 25.61 | 54.141 | *infA* | 49.10779 | -5.033206193 |
| NC_050162 | 32.48 | 38.66 | 31.56 | 27.22 | 47.21 | *matK* | 50.30249 | 3.092487833 |
| NC_050162 | 34.16 | 40.93 | 39.56 | 21.98 | 41.646 | *ndhA* | 46.35821 | 4.712212705 |
| NC_050162 | 34.16 | 40.93 | 39.56 | 21.98 | 41.646 | *ndhA* | 46.35821 | 4.712212705 |
| NC_050162 | 37.19 | 41.78 | 37.93 | 31.85 | 46.84 | *ndhB* | 53.56571 | 6.725706806 |
| NC_050162 | 34.99 | 47.11 | 31.4 | 26.45 | 56.087 | *ndhC* | 49.73385 | -6.353148423 |
| NC_050162 | 35.44 | 40.43 | 37.48 | 28.4 | 45.366 | *ndhD* | 51.16216 | 5.796161911 |
| NC_050162 | 35.44 | 40.43 | 37.48 | 28.4 | 45.366 | *ndhD* | 51.16216 | 5.796161911 |
| NC_050162 | 33.33 | 40.2 | 34.31 | 25.49 | 39.896 | *ndhE* | 49.01793 | 9.121929252 |
| NC_050162 | 33.33 | 40.2 | 34.31 | 25.49 | 39.896 | *ndhE* | 49.01793 | 9.121929252 |
| NC_050162 | 32.04 | 35.96 | 35.83 | 24.33 | 42.563 | *ndhF* | 48.14464 | 5.581643957 |
| NC_050162 | 32.77 | 41.57 | 33.71 | 23.03 | 47.492 | *ndhG* | 47.15837 | -0.333628448 |
| NC_050162 | 32.77 | 41.57 | 33.71 | 23.03 | 47.492 | *ndhG* | 47.15837 | -0.333628448 |
| NC_050162 | 37.65 | 51.02 | 36.55 | 25.38 | 49.275 | *ndhH* | 48.93547 | -0.339532528 |
| NC_050162 | 37.65 | 51.02 | 36.55 | 25.38 | 49.275 | *ndhH* | 48.93547 | -0.339532528 |
| NC_050162 | 35.77 | 42.7 | 36.52 | 28.09 | 48.067 | *ndhI* | 50.93783 | 2.870832752 |
| NC_050162 | 35.77 | 42.7 | 36.52 | 28.09 | 48.067 | *ndhI* | 50.93783 | 2.870832752 |
| NC_050162 | 38.78 | 50.31 | 37.11 | 28.93 | 45.032 | *ndhJ* | 51.54294 | 6.510939224 |
| NC_050162 | 37.32 | 43.81 | 42.48 | 25.66 | 51.108 | *ndhK* | 49.14521 | -1.962792198 |
| NC_050162 | 40.19 | 52.02 | 36.45 | 32.09 | 49.848 | *petA* | 53.72534 | 3.877338387 |
| NC_050162 | 40.19 | 52.02 | 36.45 | 32.09 | 49.848 | *petA* | 53.72534 | 3.877338387 |
| NC_050162 | 39.51 | 48.61 | 42.13 | 27.78 | 44.342 | *petB* | 50.71239 | 6.370387453 |
| NC_050162 | 37.71 | 50.62 | 39.38 | 23.12 | 45.237 | *petD* | 47.22684 | 1.989840698 |
| NC_050162 | 34.21 | 50 | 28.95 | 23.68 | 43.133 | *petG* | 47.6523 | 4.519302359 |
| NC_050162 | 32.29 | 31.25 | 43.75 | 21.88 | 55.5 | *petL* | 46.28191 | -9.218087523 |
| NC_050162 | 41.11 | 50 | 43.33 | 30 | 30.698 | *petN* | 52.3 | 21.602 |
| NC_050162 | 42.7 | 52.2 | 43.14 | 32.76 | 50.489 | *psaA* | 54.16483 | 3.675825677 |
| NC_050162 | 40.82 | 48.44 | 43.13 | 30.88 | 48.181 | *psaB* | 52.90948 | 4.728475383 |
| NC_050162 | 41.87 | 45.12 | 53.66 | 26.83 | 51.756 | *psaC* | 50.01516 | -1.740839759 |
| NC_050162 | 41.87 | 45.12 | 53.66 | 26.83 | 51.756 | *psaC* | 50.01516 | -1.740839759 |
| NC_050162 | 32.43 | 40.54 | 29.73 | 27.03 | 41.643 | *psaI* | 50.16269 | 8.519689698 |
| NC_050162 | 32.43 | 40.54 | 29.73 | 27.03 | 41.643 | *psaI* | 50.16269 | 8.519689698 |
| NC_050162 | 37.59 | 40.43 | 42.55 | 29.79 | 39.036 | *psaJ* | 52.15273 | 13.11673476 |
| NC_050162 | 41.34 | 49.44 | 43.79 | 30.79 | 41.44 | *psbA* | 52.84773 | 11.40773339 |
| NC_050162 | 44.01 | 54.62 | 45.97 | 31.43 | 48.539 | *psbB* | 53.28369 | 4.744686767 |
| NC_050162 | 44.09 | 54.01 | 46.41 | 31.86 | 45.106 | *psbC* | 53.57238 | 8.466380585 |
| NC_050162 | 42.47 | 51.98 | 43.22 | 32.2 | 46.148 | *psbD* | 53.79812 | 7.650122179 |
| NC_050162 | 41.67 | 44.05 | 47.62 | 33.33 | 50.06 | *psbE* | 54.53121 | 4.471211875 |
| NC_050162 | 39.17 | 47.5 | 45 | 25 | 44 | *psbF* | 48.65 | 4.65 |
| NC_050162 | 37.39 | 36.49 | 47.3 | 28.38 | 42.805 | *psbH* | 51.14772 | 8.34272388 |
| NC_050162 | 37.84 | 54.05 | 32.43 | 27.03 | 27.272 | *psbI* | 50.16269 | 22.8906897 |
| NC_050162 | 41.46 | 46.34 | 48.78 | 29.27 | 35.964 | *psbJ* | 51.78527 | 15.82126637 |
| NC_050162 | 36.02 | 38.71 | 33.87 | 35.48 | 54.824 | *psbK* | 55.84394 | 1.019944876 |
| NC_050162 | 32.48 | 35.9 | 25.64 | 35.9 | 52.793 | *psbL* | 56.08638 | 3.293383551 |
| NC_050162 | 29.52 | 45.71 | 25.71 | 17.14 | 47 | *psbM* | 42.67669 | -4.323313026 |
| NC_050162 | 43.18 | 56.82 | 40.91 | 31.82 | 49.02 | *psbN* | 53.54567 | 4.525673867 |
| NC_050162 | 34.26 | 38.89 | 30.56 | 33.33 | 56.893 | *psbT* | 54.53121 | -2.361788125 |
| NC_050162 | 36.51 | 41.27 | 41.27 | 26.98 | 46.925 | *psbZ* | 50.12584 | 3.200842687 |
| NC_050162 | 43.77 | 58.61 | 43.49 | 29.2 | 47.033 | *rbcL* | 51.7355 | 4.702504828 |
| NC_050162 | 42.16 | 48.51 | 48.51 | 29.48 | 49.344 | *rpl2* | 51.93414 | 2.590137689 |
| NC_050162 | 39.39 | 56.06 | 34.85 | 27.27 | 50.124 | *rpl14* | 50.33922 | 0.215217696 |
| NC_050162 | 39.66 | 46.72 | 51.09 | 21.17 | 37.651 | *rpl16* | 45.73996 | 8.088964208 |
| NC_050162 | 38.3 | 37.72 | 48.25 | 28.95 | 43.529 | *rpl20* | 51.55724 | 8.028237022 |
| NC_050162 | 34.41 | 39.35 | 39.35 | 24.52 | 42.675 | *rpl22* | 48.2882 | 5.613195139 |
| NC_050162 | 32.62 | 40.86 | 34.41 | 22.58 | 48.969 | *rpl23* | 46.81571 | -2.153285144 |
| NC_050162 | 35.78 | 35.29 | 48.53 | 23.53 | 47.675 | *rpl32* | 47.53844 | -0.136556515 |
| NC_050162 | 35.78 | 35.29 | 48.53 | 23.53 | 47.675 | *rpl32* | 47.53844 | -0.136556515 |
| NC_050162 | 36.99 | 38.36 | 35.62 | 36.99 | 50.543 | *rpl33* | 56.69207 | 6.149069653 |
| NC_050162 | 36.84 | 36.84 | 50 | 23.68 | 26.489 | *rpl36* | 47.6523 | 21.16330236 |
| NC_050162 | 34.41 | 43.17 | 35.25 | 24.82 | 45.162 | *rpoA* | 48.51447 | 3.352473444 |
| NC_050162 | 38.01 | 48.69 | 36.89 | 28.46 | 48.639 | *rpoB* | 51.20545 | 2.56644661 |
| NC_050162 | 37.79 | 49.56 | 36.48 | 27.33 | 48.306 | *rpoC1* | 50.38326 | 2.077260391 |
| NC_050162 | 36.86 | 45.37 | 36.62 | 28.59 | 49.652 | *rpoC2* | 51.29908 | 1.647077142 |
| NC_050162 | 37.29 | 41.95 | 40.25 | 29.66 | 50.105 | *rps2* | 52.06124 | 1.95623729 |
| NC_050162 | 35.15 | 44.77 | 34.31 | 26.36 | 46.493 | *rps3* | 49.66704 | 3.174042131 |
| NC_050162 | 34.47 | 46.02 | 32.39 | 25 | 48.388 | *rpS3* | 48.65 | 0.262 |
| NC_050162 | 37.16 | 46.89 | 38.76 | 25.84 | 48.416 | *rps4* | 49.27975 | 0.863749898 |
| NC_050162 | 39.53 | 51.28 | 42.31 | 25 | 46.355 | *rps7* | 48.65 | 2.295 |
| NC_050162 | 33.83 | 39.85 | 39.85 | 21.8 | 40.338 | *rps8* | 46.22086 | 5.882864738 |
| NC_050162 | 41.49 | 48.92 | 53.96 | 21.58 | 51.001 | *rps11* | 46.05296 | -4.948044933 |
| NC_050162 | 42.47 | 51.61 | 46.77 | 29.03 | 44.522 | *rps12* | 51.61437 | 7.092374785 |
| NC_050162 | 42.9 | 47.52 | 47.52 | 33.66 | 43.475 | *rps14* | 54.74001 | 11.26500823 |
| NC_050162 | 31.9 | 43.01 | 24.73 | 27.96 | 55.317 | *rps15* | 50.84342 | -4.473576284 |
| NC_050162 | 31.9 | 43.01 | 24.73 | 27.96 | 55.317 | *rps15* | 50.84342 | -4.473576284 |
| NC_050162 | 34.47 | 51.14 | 31.82 | 20.45 | 50.19 | *rps16* | 45.19036 | -4.999643088 |
| NC_050162 | 34.47 | 51.14 | 31.82 | 20.45 | 50.19 | *rps16* | 45.19036 | -4.999643088 |
| NC_050162 | 33.04 | 32.74 | 40.71 | 25.66 | 38.032 | *rps18* | 49.14521 | 11.1132078 |
| NC_050162 | 33.69 | 43.01 | 36.56 | 21.51 | 54.586 | *rps19* | 45.99952 | -8.586477706 |
| NC_050162 | 35.24 | 41.4 | 24.2 | 40.13 | 61 | *ycf1* | 58.22599 | -2.774007399 |
| NC_050162 | 38.46 | 47.93 | 38.46 | 28.99 | 51.943 | *ycf3* | 51.58582 | -0.357183378 |
| NC_050162 | 38.92 | 48.11 | 40 | 28.65 | 50.179 | *ycf4* | 51.34222 | 1.163219685 |
| NC_050162 | 38.92 | 48.11 | 40 | 28.65 | 50.179 | *ycf4* | 51.34222 | 1.163219685 |
| NC_050162 | 38.61 | 41.58 | 38.61 | 35.64 | 61 | *Ycf15* | 55.93688 | -5.063123455 |
| NC_050162 | 52.07 | 48.76 | 52.07 | 55.37 | 51.234 | *ycf68* | 59.89231 | 8.658312862 |
| NC_057644 | 38.42 | 52.21 | 32.9 | 30.15 | 50.404 | *accD* | 52.40477 | 2.000773212 |
| NC_057644 | 40.09 | 54.33 | 40.16 | 25.79 | 44.967 | *atpA* | 49.2424 | 4.27540072 |
| NC_057644 | 41.55 | 56.11 | 41.48 | 27.05 | 45.645 | *atpB* | 50.17742 | 4.532421807 |
| NC_057644 | 38.81 | 50 | 38.06 | 28.36 | 47.296 | *atpE* | 51.13328 | 3.837280975 |
| NC_057644 | 37.19 | 45.26 | 34.21 | 32.11 | 45.565 | *atpF* | 53.73859 | 8.173589769 |
| NC_057644 | 45.53 | 60.98 | 48.78 | 26.83 | 50.468 | *atpH* | 50.01516 | -0.452839759 |
| NC_057644 | 38.58 | 48.79 | 37.1 | 29.84 | 46.088 | *atpI* | 52.18786 | 6.099858722 |
| NC_057644 | 33.44 | 35.51 | 37.38 | 27.41 | 47.949 | *ccsA* | 50.44193 | 2.492927074 |
| NC_057644 | 33.48 | 39.57 | 28.26 | 32.61 | 46.464 | *cemA* | 54.06724 | 7.603241141 |
| NC_057644 | 36.59 | 47.56 | 37.8 | 24.39 | 51.463 | *infA* | 48.19 | -3.273004783 |
| NC_057644 | 32.02 | 37.6 | 31.1 | 27.36 | 47.605 | *matK* | 50.40527 | 2.800268062 |
| NC_057644 | 34.25 | 41.21 | 39.56 | 21.98 | 41.765 | *ndhA* | 46.35821 | 4.593212705 |
| NC_057644 | 37.18 | 41.83 | 37.45 | 32.27 | 48.123 | *ndhB* | 53.84431 | 5.721312659 |
| NC_057644 | 35.26 | 47.11 | 31.4 | 27.27 | 56.338 | *ndhC* | 50.33922 | -5.998782304 |
| NC_057644 | 35.44 | 40.24 | 37.48 | 28.6 | 45.15 | *ndhD* | 51.30627 | 6.156270727 |
| NC_057644 | 33.33 | 40.2 | 34.31 | 25.49 | 39.896 | *ndhE* | 49.01793 | 9.121929252 |
| NC_057644 | 31.86 | 35.7 | 35.29 | 24.6 | 44.087 | *ndhF* | 48.34858 | 4.261583016 |
| NC_057644 | 32.77 | 41.57 | 33.71 | 23.03 | 47.492 | *ndhG* | 47.15837 | -0.333628448 |
| NC_057644 | 37.65 | 51.02 | 36.55 | 25.38 | 49.275 | *ndhH* | 48.93547 | -0.339532528 |
| NC_057644 | 35.77 | 42.7 | 36.52 | 28.09 | 48.067 | *ndhI* | 50.93783 | 2.870832752 |
| NC_057644 | 38.36 | 50.31 | 35.22 | 29.56 | 46.727 | *ndhJ* | 51.99068 | 5.263684669 |
| NC_057644 | 37.02 | 43.81 | 42.48 | 24.78 | 49.848 | *ndhK* | 48.48433 | -1.363668702 |
| NC_057644 | 40.29 | 52.34 | 36.45 | 32.09 | 50.031 | *petA* | 53.72534 | 3.694338387 |
| NC_057644 | 40.23 | 48.46 | 42.73 | 29.52 | 46.817 | *petB* | 51.96242 | 5.145422757 |
| NC_057644 | 37.71 | 48.57 | 40.57 | 24 | 43.881 | *petD* | 47.89491 | 4.013911839 |
| NC_057644 | 34.21 | 50 | 28.95 | 23.68 | 43.133 | *petG* | 47.6523 | 4.519302359 |
| NC_057644 | 32.29 | 31.25 | 43.75 | 21.88 | 55.5 | *petL* | 46.28191 | -9.218087523 |
| NC_057644 | 41.11 | 50 | 43.33 | 30 | 30.698 | *petN* | 52.3 | 21.602 |
| NC_057644 | 42.7 | 52.06 | 43.14 | 32.89 | 50.61 | *psaA* | 54.24901 | 3.639011348 |
| NC_057644 | 40.82 | 48.44 | 43.13 | 30.88 | 48.353 | *psaB* | 52.90948 | 4.556475383 |
| NC_057644 | 41.87 | 45.12 | 53.66 | 26.83 | 51.756 | *psaC* | 50.01516 | -1.740839759 |
| NC_057644 | 32.43 | 40.54 | 29.73 | 27.03 | 41.643 | *psaI* | 50.16269 | 8.519689698 |
| NC_057644 | 38.52 | 40 | 40 | 35.56 | 47.459 | *psaJ* | 55.8905 | 8.431498256 |
| NC_057644 | 41.53 | 49.44 | 43.79 | 31.36 | 40.941 | *psbA* | 53.23636 | 12.2953626 |
| NC_057644 | 44.07 | 54.62 | 45.97 | 31.63 | 49.071 | *psbB* | 53.4184 | 4.347397798 |
| NC_057644 | 43.88 | 53.59 | 46.41 | 31.65 | 44.783 | *psbC* | 53.43183 | 8.648827636 |
| NC_057644 | 42.47 | 51.98 | 43.22 | 32.2 | 46.07 | *psbD* | 53.79812 | 7.728122179 |
| NC_057644 | 41.67 | 44.05 | 47.62 | 33.33 | 50.06 | *psbE* | 54.53121 | 4.471211875 |
| NC_057644 | 40 | 47.5 | 45 | 27.5 | 36.5 | *psbF* | 50.50785 | 14.00784823 |
| NC_057644 | 37.84 | 37.84 | 47.3 | 28.38 | 41.647 | *psbH* | 51.14772 | 9.50072388 |
| NC_057644 | 37.84 | 54.05 | 32.43 | 27.03 | 27.272 | *psbI* | 50.16269 | 22.8906897 |
| NC_057644 | 41.46 | 46.34 | 48.78 | 29.27 | 35.964 | *psbJ* | 51.78527 | 15.82126637 |
| NC_057644 | 35.48 | 37.1 | 33.87 | 35.48 | 48.859 | *psbK* | 55.84394 | 6.984944876 |
| NC_057644 | 32.48 | 35.9 | 25.64 | 35.9 | 52.793 | *psbL* | 56.08638 | 3.293383551 |
| NC_057644 | 30.48 | 45.71 | 25.71 | 20 | 47 | *psbM* | 44.84706 | -2.152941176 |
| NC_057644 | 42.42 | 54.55 | 40.91 | 31.82 | 46.02 | *psbN* | 53.54567 | 7.525673867 |
| NC_057644 | 34.26 | 38.89 | 30.56 | 33.33 | 56.893 | *psbT* | 54.53121 | -2.361788125 |
| NC_057644 | 36.51 | 41.27 | 41.27 | 26.98 | 47.179 | *psbZ* | 50.12584 | 2.946842687 |
| NC_057644 | 43.35 | 58.19 | 43.07 | 28.78 | 46.569 | *rbcL* | 51.43554 | 4.866537557 |
| NC_057644 | 42.29 | 47.76 | 48.51 | 30.6 | 51.751 | *rpl2* | 52.71694 | 0.965936044 |
| NC_057644 | 38.35 | 53.38 | 34.59 | 27.07 | 52.35 | *rpl14* | 50.19215 | -2.157849933 |
| NC_057644 | 39.72 | 49.17 | 51.67 | 18.33 | 35.35 | *rpl16* | 43.57652 | 8.226522799 |
| NC_057644 | 38.01 | 36.84 | 48.25 | 28.95 | 43.234 | *rpl20* | 51.55724 | 8.323237022 |
| NC_057644 | 34.41 | 39.35 | 39.35 | 24.52 | 41.791 | *rpl22* | 48.2882 | 6.497195139 |
| NC_057644 | 32.99 | 40.62 | 33.33 | 25 | 49.603 | *rpl23* | 48.65 | -0.953 |
| NC_057644 | 35.78 | 35.29 | 48.53 | 23.53 | 47.675 | *rpl32* | 47.53844 | -0.136556515 |
| NC_057644 | 38.03 | 40.85 | 36.62 | 36.62 | 53.361 | *rpl33* | 56.49038 | 3.129380429 |
| NC_057644 | 36.84 | 39.47 | 47.37 | 23.68 | 36.35 | *rpl36* | 47.6523 | 11.30230236 |
| NC_057644 | 34.42 | 43.27 | 35.64 | 24.36 | 45.566 | *rpoA* | 48.16732 | 2.601321775 |
| NC_057644 | 38.18 | 48.88 | 36.78 | 28.86 | 48.882 | *rpoB* | 51.49286 | 2.610855211 |
| NC_057644 | 37.66 | 49.27 | 36.88 | 26.82 | 47.808 | *rpoC1* | 50.00777 | 2.199773973 |
| NC_057644 | 36.75 | 45.38 | 36.58 | 28.29 | 49.549 | *rpoC2* | 51.08269 | 1.533692658 |
| NC_057644 | 37.43 | 41.53 | 40.25 | 30.51 | 51.936 | *rps2* | 52.65477 | 0.718768027 |
| NC_057644 | 34.73 | 44.35 | 34.31 | 25.52 | 48.929 | *rps3* | 49.0404 | 0.111404673 |
| NC_057644 | 37 | 46.41 | 38.28 | 26.32 | 48.144 | *rps4* | 49.63733 | 1.493327271 |
| NC_057644 | 39.32 | 51.28 | 42.31 | 24.36 | 45.605 | *rps7* | 48.16732 | 2.562321775 |
| NC_057644 | 34.09 | 40.6 | 39.85 | 21.8 | 40.502 | *rps8* | 46.22086 | 5.718864738 |
| NC_057644 | 41.73 | 49.64 | 53.96 | 21.58 | 50.703 | *rps11* | 46.05296 | -4.650044933 |
| NC_057644 | 41.67 | 51.61 | 46.77 | 26.61 | 45.738 | *rps12* | 49.85245 | 4.114452903 |
| NC_057644 | 41.91 | 45.54 | 46.53 | 33.66 | 45.876 | *rps14* | 54.74001 | 8.864008231 |
| NC_057644 | 32.62 | 43.01 | 26.88 | 27.96 | 52.599 | *rps15* | 50.84342 | -1.755576284 |
| NC_057644 | 34.1 | 50.57 | 31.03 | 20.69 | 50.766 | *rps16* | 45.37353 | -5.392468556 |
| NC_057644 | 33.04 | 34.51 | 38.94 | 25.66 | 38.06 | *rps18* | 49.14521 | 11.0852078 |
| NC_057644 | 33.69 | 43.01 | 36.56 | 21.51 | 48.543 | *rps19* | 45.99952 | -2.543477706 |
| NC_057644 | 38.66 | 47.93 | 38.46 | 29.59 | 51.258 | *ycf3* | 52.01187 | 0.75386583 |
| NC_057644 | 39.1 | 48.65 | 40 | 28.65 | 50.839 | *ycf4* | 51.34222 | 0.503219685 |
| NC_057644 | 39.29 | 42.86 | 30.36 | 44.64 | 58.587 | *ycf15* | 59.78745 | 1.200445875 |
| NC_058233 | 38.57 | 52.87 | 31.42 | 31.42 | 50.415 | *accD* | 53.27693 | 2.861931692 |
| NC_058233 | 40.09 | 54.33 | 40.16 | 25.79 | 44.967 | *atpA* | 49.2424 | 4.27540072 |
| NC_058233 | 41.55 | 56.11 | 41.48 | 27.05 | 45.645 | *atpB* | 50.17742 | 4.532421807 |
| NC_058233 | 38.81 | 50 | 38.06 | 28.36 | 47.296 | *atpE* | 51.13328 | 3.837280975 |
| NC_058233 | 37.19 | 45.26 | 34.21 | 32.11 | 45.565 | *atpF* | 53.73859 | 8.173589769 |
| NC_058233 | 45.53 | 60.98 | 48.78 | 26.83 | 50.468 | *atpH* | 50.01516 | -0.452839759 |
| NC_058233 | 38.58 | 48.79 | 37.1 | 29.84 | 46.088 | *atpI* | 52.18786 | 6.099858722 |
| NC_058233 | 33.44 | 35.51 | 37.38 | 27.41 | 47.916 | *ccsA* | 50.44193 | 2.525927074 |
| NC_058233 | 33.48 | 39.57 | 28.26 | 32.61 | 46.464 | *cemA* | 54.06724 | 7.603241141 |
| NC_058233 | 36.59 | 47.56 | 37.8 | 24.39 | 51.463 | *infA* | 48.19 | -3.273004783 |
| NC_058233 | 32.09 | 37.6 | 31.3 | 27.36 | 47.655 | *matK* | 50.40527 | 2.750268062 |
| NC_058233 | 34.25 | 41.21 | 39.56 | 21.98 | 41.765 | *ndhA* | 46.35821 | 4.593212705 |
| NC_058233 | 37.18 | 41.83 | 37.45 | 32.27 | 48.123 | *ndhB* | 53.84431 | 5.721312659 |
| NC_058233 | 35.26 | 47.11 | 31.4 | 27.27 | 56.338 | *ndhC* | 50.33922 | -5.998782304 |
| NC_058233 | 35.5 | 40.24 | 37.48 | 28.8 | 45.253 | *ndhD* | 51.44987 | 6.196874797 |
| NC_058233 | 33.33 | 40.2 | 34.31 | 25.49 | 39.896 | *ndhE* | 49.01793 | 9.121929252 |
| NC_058233 | 31.91 | 35.83 | 35.29 | 24.6 | 44.174 | *ndhF* | 48.34858 | 4.174583016 |
| NC_058233 | 32.77 | 41.57 | 33.71 | 23.03 | 47.492 | *ndhG* | 47.15837 | -0.333628448 |
| NC_058233 | 37.65 | 51.02 | 36.55 | 25.38 | 49.275 | *ndhH* | 48.93547 | -0.339532528 |
| NC_058233 | 35.77 | 42.7 | 36.52 | 28.09 | 48.067 | *ndhI* | 50.93783 | 2.870832752 |
| NC_058233 | 38.36 | 50.31 | 35.22 | 29.56 | 46.727 | *ndhJ* | 51.99068 | 5.263684669 |
| NC_058233 | 37.17 | 44.25 | 42.48 | 24.78 | 50.268 | *ndhK* | 48.48433 | -1.783668702 |
| NC_058233 | 40.29 | 52.34 | 36.45 | 32.09 | 50.031 | *petA* | 53.72534 | 3.694338387 |
| NC_058233 | 40.23 | 48.46 | 42.73 | 29.52 | 46.817 | *petB* | 51.96242 | 5.145422757 |
| NC_058233 | 37.71 | 48.57 | 40.57 | 24 | 43.881 | *petD* | 47.89491 | 4.013911839 |
| NC_058233 | 34.21 | 50 | 28.95 | 23.68 | 43.133 | *petG* | 47.6523 | 4.519302359 |
| NC_058233 | 32.29 | 31.25 | 43.75 | 21.88 | 55.5 | *petL* | 46.28191 | -9.218087523 |
| NC_058233 | 41.11 | 50 | 43.33 | 30 | 30.698 | *petN* | 52.3 | 21.602 |
| NC_058233 | 42.7 | 52.06 | 43.14 | 32.89 | 50.61 | *psaA* | 54.24901 | 3.639011348 |
| NC_058233 | 40.82 | 48.44 | 43.13 | 30.88 | 48.322 | *psaB* | 52.90948 | 4.587475383 |
| NC_058233 | 41.87 | 45.12 | 53.66 | 26.83 | 51.756 | *psaC* | 50.01516 | -1.740839759 |
| NC_058233 | 32.43 | 40.54 | 29.73 | 27.03 | 41.643 | *psaI* | 50.16269 | 8.519689698 |
| NC_058233 | 38.52 | 40 | 40 | 35.56 | 47.459 | *psaJ* | 55.8905 | 8.431498256 |
| NC_058233 | 41.53 | 49.44 | 43.79 | 31.36 | 40.941 | *psbA* | 53.23636 | 12.2953626 |
| NC_058233 | 44.14 | 54.62 | 45.97 | 31.83 | 49.293 | *psbB* | 53.55235 | 4.259353445 |
| NC_058233 | 43.88 | 53.59 | 46.41 | 31.65 | 44.783 | *psbC* | 53.43183 | 8.648827636 |
| NC_058233 | 42.47 | 51.98 | 43.22 | 32.2 | 46.07 | *psbD* | 53.79812 | 7.728122179 |
| NC_058233 | 41.67 | 44.05 | 47.62 | 33.33 | 50.06 | *psbE* | 54.53121 | 4.471211875 |
| NC_058233 | 40 | 47.5 | 45 | 27.5 | 36.5 | *psbF* | 50.50785 | 14.00784823 |
| NC_058233 | 37.84 | 37.84 | 47.3 | 28.38 | 41.647 | *psbH* | 51.14772 | 9.50072388 |
| NC_058233 | 37.84 | 54.05 | 32.43 | 27.03 | 27.272 | *psbI* | 50.16269 | 22.8906897 |
| NC_058233 | 41.46 | 46.34 | 48.78 | 29.27 | 35.964 | *psbJ* | 51.78527 | 15.82126637 |
| NC_058233 | 35.48 | 37.1 | 33.87 | 35.48 | 48.859 | *psbK* | 55.84394 | 6.984944876 |
| NC_058233 | 32.48 | 35.9 | 25.64 | 35.9 | 52.793 | *psbL* | 56.08638 | 3.293383551 |
| NC_058233 | 30.48 | 45.71 | 25.71 | 20 | 47 | *psbM* | 44.84706 | -2.152941176 |
| NC_058233 | 42.42 | 54.55 | 40.91 | 31.82 | 46.02 | *psbN* | 53.54567 | 7.525673867 |
| NC_058233 | 34.26 | 38.89 | 30.56 | 33.33 | 56.893 | *psbT* | 54.53121 | -2.361788125 |
| NC_058233 | 36.51 | 41.27 | 41.27 | 26.98 | 47.179 | *psbZ* | 50.12584 | 2.946842687 |
| NC_058233 | 43.35 | 58.19 | 43.07 | 28.78 | 46.569 | *rbcL* | 51.43554 | 4.866537557 |
| NC_058233 | 42.29 | 47.76 | 48.51 | 30.6 | 51.751 | *rpl2* | 52.71694 | 0.965936044 |
| NC_058233 | 38.35 | 52.63 | 35.34 | 27.07 | 50.276 | *rpl14* | 50.19215 | -0.083849933 |
| NC_058233 | 40 | 49.17 | 51.67 | 19.17 | 35.744 | *rpl16* | 44.21473 | 8.470730418 |
| NC_058233 | 38.01 | 36.84 | 48.25 | 28.95 | 43.234 | *rpl20* | 51.55724 | 8.323237022 |
| NC_058233 | 34.62 | 39.35 | 39.35 | 25.16 | 43.264 | *rpl22* | 48.77031 | 5.506306808 |
| NC_058233 | 32.99 | 40.62 | 33.33 | 25 | 49.603 | *rpl23* | 48.65 | -0.953 |
| NC_058233 | 35.78 | 35.29 | 48.53 | 23.53 | 47.675 | *rpl32* | 47.53844 | -0.136556515 |
| NC_058233 | 38.03 | 40.85 | 36.62 | 36.62 | 53.361 | *rpl33* | 56.49038 | 3.129380429 |
| NC_058233 | 36.84 | 39.47 | 47.37 | 23.68 | 36.35 | *rpl36* | 47.6523 | 11.30230236 |
| NC_058233 | 34.42 | 43.27 | 35.64 | 24.36 | 45.648 | *rpoA* | 48.16732 | 2.519321775 |
| NC_058233 | 38.08 | 48.79 | 36.69 | 28.77 | 48.793 | *rpoB* | 51.42837 | 2.635366989 |
| NC_058233 | 37.66 | 49.27 | 36.73 | 26.97 | 47.986 | *rpoC1* | 50.11847 | 2.13247043 |
| NC_058233 | 36.75 | 45.38 | 36.58 | 28.29 | 49.549 | *rpoC2* | 51.08269 | 1.533692658 |
| NC_058233 | 37.43 | 41.53 | 40.25 | 30.51 | 51.936 | *rps2* | 52.65477 | 0.718768027 |
| NC_058233 | 34.73 | 44.35 | 34.31 | 25.52 | 48.929 | *rps3* | 49.0404 | 0.111404673 |
| NC_058233 | 36.84 | 46.41 | 38.28 | 25.84 | 47.503 | *rps4* | 49.27975 | 1.776749898 |
| NC_058233 | 39.32 | 51.28 | 42.31 | 24.36 | 45.605 | *rps7* | 48.16732 | 2.562321775 |
| NC_058233 | 34.09 | 40.6 | 39.85 | 21.8 | 40.502 | *rps8* | 46.22086 | 5.718864738 |
| NC_058233 | 41.73 | 49.64 | 53.96 | 21.58 | 50.703 | *rps11* | 46.05296 | -4.650044933 |
| NC_058233 | 41.67 | 51.61 | 46.77 | 26.61 | 45.738 | *rps12* | 49.85245 | 4.114452903 |
| NC_058233 | 41.91 | 45.54 | 46.53 | 33.66 | 45.876 | *rps14* | 54.74001 | 8.864008231 |
| NC_058233 | 32.62 | 43.01 | 26.88 | 27.96 | 52.599 | *rps15* | 50.84342 | -1.755576284 |
| NC_058233 | 34.1 | 50.57 | 31.03 | 20.69 | 50.766 | *rps16* | 45.37353 | -5.392468556 |
| NC_058233 | 33.04 | 34.51 | 38.94 | 25.66 | 38.06 | *rps18* | 49.14521 | 11.0852078 |
| NC_058233 | 33.69 | 43.01 | 36.56 | 21.51 | 48.543 | *rps19* | 45.99952 | -2.543477706 |
| NC_058233 | 38.66 | 47.93 | 38.46 | 29.59 | 51.258 | *ycf3* | 52.01187 | 0.75386583 |
| NC_058233 | 39.1 | 48.65 | 40 | 28.65 | 50.839 | *ycf4* | 51.34222 | 0.503219685 |
| NC_058233 | 39.88 | 44.64 | 30.36 | 44.64 | 58.587 | *ycf15* | 59.78745 | 1.200445875 |
| MN182619.2 | 37.01 | 51.1 | 29.71 | 30.22 | 49.968 | *accD* | 52.45355 | 2.485546387 |
| MN182619.2 | 40.03 | 54.33 | 40.16 | 25.59 | 44.396 | *atpA* | 49.09282 | 4.696823297 |
| MN182619.2 | 41.88 | 56.31 | 41.68 | 27.66 | 46.197 | *atpB* | 50.62483 | 4.427831035 |
| MN182619.2 | 38.31 | 48.51 | 38.06 | 28.36 | 45.286 | *atpE* | 51.13328 | 5.847280975 |
| MN182619.2 | 38.02 | 45.95 | 34.59 | 33.51 | 44.695 | *atpF* | 54.64541 | 9.950410099 |
| MN182619.2 | 45.53 | 60.98 | 48.78 | 26.83 | 50.468 | *atpH* | 50.01516 | -0.452839759 |
| MN182619.2 | 38.84 | 49.19 | 37.1 | 30.24 | 46.118 | *atpI* | 52.46747 | 6.349467289 |
| MN182619.2 | 33.33 | 35.2 | 37.38 | 27.41 | 47.885 | *ccsA* | 50.44193 | 2.556927074 |
| MN182619.2 | 33.62 | 39.57 | 28.26 | 33.04 | 46.487 | *cemA* | 54.34569 | 7.858694936 |
| MN182619.2 | 35.77 | 46.34 | 37.8 | 23.17 | 50.637 | *infA* | 47.26487 | -3.372130897 |
| MN182619.2 | 32.61 | 39.05 | 31.36 | 27.42 | 47.32 | *matK* | 50.44926 | 3.129255793 |
| MN182619.2 | 34.34 | 41.21 | 39.56 | 22.25 | 41.855 | *ndhA* | 46.56416 | 4.709160136 |
| MN182619.2 | 36.98 | 41.38 | 38.13 | 31.44 | 45.878 | *ndhB* | 53.29044 | 7.412439996 |
| MN182619.2 | 35.54 | 47.11 | 31.4 | 28.1 | 57.129 | *ndhC* | 50.94509 | -6.183913089 |
| MN182619.2 | 35.33 | 40.12 | 37.92 | 27.94 | 45.185 | *ndhD* | 50.82888 | 5.643882101 |
| MN182619.2 | 33.01 | 40.2 | 34.31 | 24.51 | 40.342 | *ndhE* | 48.28064 | 7.93864433 |
| MN182619.2 | 31.82 | 35.83 | 35.29 | 24.33 | 43.044 | *ndhF* | 48.14464 | 5.100643957 |
| MN182619.2 | 32.77 | 41.57 | 33.71 | 23.03 | 47.73 | *ndhG* | 47.15837 | -0.571628448 |
| MN182619.2 | 37.65 | 51.02 | 36.55 | 25.38 | 49.275 | *ndhH* | 48.93547 | -0.339532528 |
| MN182619.2 | 35.98 | 42.61 | 36.93 | 28.41 | 48.052 | *ndhI* | 51.16938 | 3.117379095 |
| MN182619.2 | 38.78 | 49.69 | 36.48 | 30.19 | 47.744 | *ndhJ* | 52.43265 | 4.688653105 |
| MN182619.2 | 36.81 | 43.91 | 42.17 | 24.35 | 49.309 | *ndhK* | 48.15976 | -1.149237014 |
| MN182619.2 | 38.66 | 47.93 | 38.46 | 29.59 | 51.586 | *pafI* | 52.01187 | 0.42586583 |
| MN182619.2 | 38.38 | 48.11 | 39.46 | 27.57 | 48.808 | *pafII* | 50.55906 | 1.751061663 |
| MN182619.2 | 42.42 | 54.55 | 40.91 | 31.82 | 46.02 | *pbf1* | 53.54567 | 7.525673867 |
| MN182619.2 | 39.98 | 51.71 | 36.45 | 31.78 | 49.443 | *petA* | 53.51894 | 4.075936284 |
| MN182619.2 | 39.51 | 48.15 | 42.13 | 28.24 | 44.175 | *petB* | 51.04652 | 6.87152209 |
| MN182619.2 | 37.71 | 51.25 | 39.38 | 22.5 | 44.702 | *petD* | 46.75475 | 2.05275048 |
| MN182619.2 | 34.21 | 50 | 28.95 | 23.68 | 43.133 | *petG* | 47.6523 | 4.519302359 |
| MN182619.2 | 31.25 | 28.12 | 43.75 | 21.88 | 55.5 | *petL* | 46.28191 | -9.218087523 |
| MN182619.2 | 41.11 | 50 | 43.33 | 30 | 30.698 | *petN* | 52.3 | 21.602 |
| MN182619.2 | 42.52 | 52.06 | 43.01 | 32.49 | 50.515 | *psaA* | 53.98883 | 3.47383317 |
| MN182619.2 | 40.91 | 48.44 | 42.99 | 31.29 | 48.467 | *psaB* | 53.18895 | 4.721948718 |
| MN182619.2 | 41.87 | 45.12 | 53.66 | 26.83 | 51.756 | *psaC* | 50.01516 | -1.740839759 |
| MN182619.2 | 32.43 | 40.54 | 29.73 | 27.03 | 41.643 | *psaI* | 50.16269 | 8.519689698 |
| MN182619.2 | 36.88 | 40.43 | 40.43 | 29.79 | 37.61 | *psaJ* | 52.15273 | 14.54273476 |
| MN182619.2 | 41.24 | 49.44 | 43.79 | 30.51 | 40.206 | *psbA* | 52.65477 | 12.44876803 |
| MN182619.2 | 44.14 | 54.81 | 45.97 | 31.63 | 48.625 | *psbB* | 53.4184 | 4.793397798 |
| MN182619.2 | 44.16 | 54.98 | 46.54 | 30.95 | 45.441 | *psbC* | 52.9574 | 7.516400164 |
| MN182619.2 | 42.47 | 51.98 | 43.22 | 32.2 | 45.766 | *psbD* | 53.79812 | 8.032122179 |
| MN182619.2 | 41.67 | 44.05 | 47.62 | 33.33 | 45.12 | *psbE* | 54.53121 | 9.411211875 |
| MN182619.2 | 39.17 | 47.5 | 45 | 25 | 44 | *psbF* | 48.65 | 4.65 |
| MN182619.2 | 37.84 | 37.84 | 47.3 | 28.38 | 41.647 | *psbH* | 51.14772 | 9.50072388 |
| MN182619.2 | 36.94 | 54.05 | 32.43 | 24.32 | 25.494 | *psbI* | 48.13708 | 22.64308372 |
| MN182619.2 | 41.46 | 46.34 | 48.78 | 29.27 | 35.964 | *psbJ* | 51.78527 | 15.82126637 |
| MN182619.2 | 35.48 | 38.71 | 33.87 | 33.87 | 50.008 | *psbK* | 54.87156 | 4.863564832 |
| MN182619.2 | 32.48 | 35.9 | 25.64 | 35.9 | 52.793 | *psbL* | 56.08638 | 3.293383551 |
| MN182619.2 | 29.52 | 45.71 | 25.71 | 17.14 | 47 | *psbM* | 42.67669 | -4.323313026 |
| MN182619.2 | 34.26 | 38.89 | 30.56 | 33.33 | 56.893 | *psbT* | 54.53121 | -2.361788125 |
| MN182619.2 | 36.51 | 41.27 | 41.27 | 26.98 | 46.925 | *psbZ* | 50.12584 | 3.200842687 |
| MN182619.2 | 43.49 | 58.4 | 43.28 | 28.78 | 47.347 | *rbcL* | 51.43554 | 4.088537557 |
| MN182619.2 | 42.29 | 47.76 | 48.88 | 30.22 | 50.449 | *rpl2* | 52.45355 | 2.004546387 |
| MN182619.2 | 39.6 | 54.89 | 37.59 | 26.32 | 48.262 | *rpl14* | 49.63733 | 1.375327271 |
| MN182619.2 | 39.71 | 47.79 | 51.47 | 19.85 | 38.978 | *rpl16* | 44.73269 | 5.754686853 |
| MN182619.2 | 37.43 | 36.84 | 47.37 | 28.07 | 43 | *rpl20* | 50.92332 | 7.923320953 |
| MN182619.2 | 33.76 | 39.35 | 38.06 | 23.87 | 41.773 | *rpl22* | 47.7964 | 6.023401515 |
| MN182619.2 | 33.33 | 40.62 | 34.38 | 25 | 50.734 | *rpl23* | 48.65 | -2.084 |
| MN182619.2 | 35.78 | 35.29 | 48.53 | 23.53 | 47.675 | *rpl32* | 47.53844 | -0.136556515 |
| MN182619.2 | 36.53 | 36.99 | 35.62 | 36.99 | 52.088 | *rpl33* | 56.69207 | 4.604069653 |
| MN182619.2 | 36.84 | 39.47 | 50 | 21.05 | 31.625 | *rpl36* | 45.64835 | 14.02335129 |
| MN182619.2 | 34.98 | 44.11 | 36.12 | 24.71 | 42.861 | *rpoA* | 48.43156 | 5.570561184 |
| MN182619.2 | 37.84 | 48.17 | 36.81 | 28.54 | 48 | *rpoB* | 51.26309 | 3.263090358 |
| MN182619.2 | 37.8 | 49.13 | 36.88 | 27.41 | 48.247 | *rpoC1* | 50.44193 | 2.194927074 |
| MN182619.2 | 36.83 | 45.15 | 36.69 | 28.67 | 49.539 | *rpoC2* | 51.35659 | 1.81759038 |
| MN182619.2 | 37.41 | 41.77 | 40.51 | 29.96 | 50.514 | *rps2* | 52.272 | 1.758001449 |
| MN182619.2 | 34.89 | 44.92 | 34.75 | 25 | 49.019 | *rps3* | 48.65 | -0.369 |
| MN182619.2 | 34.14 | 46.06 | 30.91 | 25.45 | 48.905 | *rpS3* | 48.98795 | 0.08295253 |
| MN182619.2 | 37 | 46.89 | 38.76 | 25.36 | 47.179 | *rps4* | 48.92047 | 1.741465779 |
| MN182619.2 | 39.74 | 51.28 | 42.31 | 25.64 | 46.54 | *rps7* | 49.13024 | 2.590244317 |
| MN182619.2 | 34.09 | 39.85 | 39.1 | 23.31 | 44.244 | *rps8* | 47.37131 | 3.127308681 |
| MN182619.2 | 41.25 | 48.2 | 53.96 | 21.58 | 51.754 | *rps11* | 46.05296 | -5.701044933 |
| MN182619.2 | 41.67 | 50.81 | 46.77 | 27.42 | 44.217 | *rps12* | 50.44926 | 6.232255793 |
| MN182619.2 | 43.23 | 47.52 | 48.51 | 33.66 | 42.883 | *rps14* | 54.74001 | 11.85700823 |
| MN182619.2 | 33.33 | 43.01 | 29.03 | 27.96 | 46.014 | *rps15* | 50.84342 | 4.829423716 |
| MN182619.2 | 34.85 | 51.14 | 31.82 | 21.59 | 48.645 | *rps16* | 46.06059 | -2.584411915 |
| MN182619.2 | 32.74 | 32.74 | 41.59 | 23.89 | 37.451 | *rps18* | 47.81156 | 10.36056151 |
| MN182619.2 | 33.95 | 41.67 | 35.19 | 25 | 50.818 | *rps19* | 48.65 | -2.168 |
| MN182619.2 | 35.68 | 43.66 | 36.62 | 26.76 | 52.033 | *rps19* | 49.96344 | -2.069562866 |
| MN182619.2 | 36.55 | 37.75 | 32.93 | 38.96 | 55.311 | *ycf1* | 57.69339 | 2.382394039 |
| MN182619.2 | 38.61 | 41.58 | 39.6 | 34.65 | 61 | *Ycf15* | 55.35089 | -5.649110688 |
| MN182619.2 | 51.2 | 50.4 | 48 | 55.2 | 61 | *ycf68* | 59.93138 | -1.068615424 |
| MT239364 | 37.49 | 29.42 | 31.24 | 51.82 | 50.143 | *accD* | 60.44145 | 10.29845401 |
| MT239364 | 40.03 | 54.33 | 40.16 | 25.59 | 44.396 | *atpA* | 49.09282 | 4.696823297 |
| MT239364 | 41.88 | 56.31 | 41.68 | 27.66 | 46.197 | *atpB* | 50.62483 | 4.427831035 |
| MT239364 | 38.31 | 48.51 | 38.06 | 28.36 | 45.286 | *atpE* | 51.13328 | 5.847280975 |
| MT239364 | 38.02 | 45.95 | 34.59 | 33.51 | 44.695 | *atpF* | 54.64541 | 9.950410099 |
| MT239364 | 45.53 | 60.98 | 48.78 | 26.83 | 50.468 | *atpH* | 50.01516 | -0.452839759 |
| MT239364 | 38.84 | 49.19 | 37.1 | 30.24 | 46.118 | *atpI* | 52.46747 | 6.349467289 |
| MT239364 | 33.33 | 35.2 | 37.38 | 27.41 | 47.885 | *ccsA* | 50.44193 | 2.556927074 |
| MT239364 | 33.48 | 39.57 | 28.26 | 32.61 | 46.464 | *cemA* | 54.06724 | 7.603241141 |
| MT239364 | 35.77 | 46.34 | 37.8 | 23.17 | 50.637 | *infA* | 47.26487 | -3.372130897 |
| MT239364 | 32.61 | 39.05 | 31.36 | 27.42 | 47.32 | *matK* | 50.44926 | 3.129255793 |
| MT239364 | 34.25 | 41.21 | 39.56 | 21.98 | 41.765 | *ndhA* | 46.35821 | 4.593212705 |
| MT239364 | 37.05 | 41.58 | 38.13 | 31.44 | 46.017 | *ndhB* | 53.29044 | 7.273439996 |
| MT239364 | 35.54 | 47.11 | 31.4 | 28.1 | 57.129 | *ndhC* | 50.94509 | -6.183913089 |
| MT239364 | 35.58 | 40.29 | 38.04 | 28.43 | 45.36 | *ndhD* | 51.18381 | 5.823809787 |
| MT239364 | 32.68 | 40.2 | 34.31 | 23.53 | 39.819 | *ndhE* | 47.53844 | 7.719443485 |
| MT239364 | 31.73 | 35.83 | 35.16 | 24.2 | 42.783 | *ndhF* | 48.04632 | 5.263323928 |
| MT239364 | 32.77 | 41.57 | 33.71 | 23.03 | 47.492 | *ndhG* | 47.15837 | -0.333628448 |
| MT239364 | 37.65 | 51.02 | 36.55 | 25.38 | 49.275 | *ndhH* | 48.93547 | -0.339532528 |
| MT239364 | 35.98 | 42.61 | 36.93 | 28.41 | 48.052 | *ndhI* | 51.16938 | 3.117379095 |
| MT239364 | 38.57 | 49.69 | 36.48 | 29.56 | 46.506 | *ndhJ* | 51.99068 | 5.484684669 |
| MT239364 | 36.81 | 43.91 | 42.17 | 24.35 | 49.309 | *ndhK* | 48.15976 | -1.149237014 |
| MT239364 | 38.66 | 47.93 | 38.46 | 29.59 | 51.586 | *pafI* | 52.01187 | 0.42586583 |
| MT239364 | 38.38 | 48.11 | 39.46 | 27.57 | 48.808 | *pafII* | 50.55906 | 1.751061663 |
| MT239364 | 42.42 | 54.55 | 40.91 | 31.82 | 46.02 | *pbf1* | 53.54567 | 7.525673867 |
| MT239364 | 39.98 | 52.02 | 36.45 | 31.46 | 49.098 | *petA* | 53.30394 | 4.205940907 |
| MT239364 | 39.66 | 48.61 | 42.13 | 28.24 | 44.435 | *petB* | 51.04652 | 6.61152209 |
| MT239364 | 37.47 | 50.93 | 39.13 | 22.36 | 44.702 | *petD* | 46.64803 | 1.946033365 |
| MT239364 | 34.21 | 50 | 28.95 | 23.68 | 43.133 | *petG* | 47.6523 | 4.519302359 |
| MT239364 | 31.25 | 28.12 | 43.75 | 21.88 | 55.5 | *petL* | 46.28191 | -9.218087523 |
| MT239364 | 41.11 | 50 | 43.33 | 30 | 30.698 | *petN* | 52.3 | 21.602 |
| MT239364 | 42.52 | 52.06 | 43.14 | 32.36 | 50.166 | *psaA* | 53.90355 | 3.737554479 |
| MT239364 | 40.86 | 48.44 | 42.99 | 31.16 | 48.349 | *psaB* | 53.10066 | 4.751658818 |
| MT239364 | 41.87 | 45.12 | 53.66 | 26.83 | 51.756 | *psaC* | 50.01516 | -1.740839759 |
| MT239364 | 32.43 | 40.54 | 29.73 | 27.03 | 41.643 | *psaI* | 50.16269 | 8.519689698 |
| MT239364 | 36.88 | 40.43 | 40.43 | 29.79 | 37.61 | *psaJ* | 52.15273 | 14.54273476 |
| MT239364 | 41.24 | 49.44 | 43.79 | 30.51 | 40.206 | *psbA* | 52.65477 | 12.44876803 |
| MT239364 | 44.01 | 54.81 | 45.97 | 31.24 | 48.563 | *psbB* | 53.15503 | 4.592027132 |
| MT239364 | 44.3 | 54.7 | 46.58 | 31.62 | 45.752 | *psbC* | 53.41168 | 7.659680045 |
| MT239364 | 42.47 | 51.98 | 43.22 | 32.2 | 45.766 | *psbD* | 53.79812 | 8.032122179 |
| MT239364 | 41.67 | 44.05 | 47.62 | 33.33 | 45.12 | *psbE* | 54.53121 | 9.411211875 |
| MT239364 | 39.17 | 47.5 | 45 | 25 | 44 | *psbF* | 48.65 | 4.65 |
| MT239364 | 37.84 | 37.84 | 47.3 | 28.38 | 41.647 | *psbH* | 51.14772 | 9.50072388 |
| MT239364 | 37.84 | 54.05 | 32.43 | 27.03 | 27.272 | *psbI* | 50.16269 | 22.8906897 |
| MT239364 | 42.28 | 46.34 | 48.78 | 31.71 | 38.933 | *psbJ* | 53.47207 | 14.53907166 |
| MT239364 | 35.48 | 38.71 | 33.87 | 33.87 | 50.008 | *psbK* | 54.87156 | 4.863564832 |
| MT239364 | 32.48 | 35.9 | 25.64 | 35.9 | 52.793 | *psbL* | 56.08638 | 3.293383551 |
| MT239364 | 29.52 | 45.71 | 25.71 | 17.14 | 47 | *psbM* | 42.67669 | -4.323313026 |
| MT239364 | 34.26 | 38.89 | 30.56 | 33.33 | 56.893 | *psbT* | 54.53121 | -2.361788125 |
| MT239364 | 36.51 | 41.27 | 41.27 | 26.98 | 46.925 | *psbZ* | 50.12584 | 3.200842687 |
| MT239364 | 43.56 | 58.4 | 43.28 | 28.99 | 47.34 | *rbcL* | 51.58582 | 4.245816622 |
| MT239364 | 42.29 | 47.76 | 48.88 | 30.22 | 50.449 | *rpl2* | 52.45355 | 2.004546387 |
| MT239364 | 39.35 | 54.89 | 36.84 | 26.32 | 48.59 | *rpl14* | 49.63733 | 1.047327271 |
| MT239364 | 39.71 | 47.79 | 51.47 | 19.85 | 38.978 | *rpl16* | 44.73269 | 5.754686853 |
| MT239364 | 37.72 | 37.72 | 47.37 | 28.07 | 43 | *rpl20* | 50.92332 | 7.923320953 |
| MT239364 | 34.19 | 39.35 | 38.71 | 24.52 | 41.978 | *rpl22* | 48.2882 | 6.310195139 |
| MT239364 | 33.33 | 40.62 | 34.38 | 25 | 50.734 | *rpl23* | 48.65 | -2.084 |
| MT239364 | 35.78 | 35.29 | 48.53 | 23.53 | 47.675 | *rpl32* | 47.53844 | -0.136556515 |
| MT239364 | 36.99 | 38.36 | 35.62 | 36.99 | 54.783 | *rpl33* | 56.69207 | 1.909069653 |
| MT239364 | 36.84 | 39.47 | 50 | 21.05 | 31.625 | *rpl36* | 45.64835 | 14.02335129 |
| MT239364 | 34.42 | 43.64 | 35.64 | 24 | 43.537 | *rpoA* | 47.89491 | 4.357911839 |
| MT239364 | 38 | 48.45 | 36.81 | 28.73 | 48.054 | *rpoB* | 51.39967 | 3.345671782 |
| MT239364 | 37.9 | 49.27 | 36.88 | 27.55 | 48.293 | *rpoC1* | 50.54443 | 2.25143453 |
| MT239364 | 36.86 | 45.37 | 36.69 | 28.52 | 49.577 | *rpoC2* | 51.24869 | 1.671686881 |
| MT239364 | 37.41 | 41.77 | 40.51 | 29.96 | 50.514 | *rps2* | 52.272 | 1.758001449 |
| MT239364 | 34.75 | 44.92 | 34.32 | 25 | 49.38 | *rps3* | 48.65 | -0.73 |
| MT239364 | 34.14 | 46.06 | 30.91 | 25.45 | 48.731 | *rpS3* | 48.98795 | 0.25695253 |
| MT239364 | 37 | 46.89 | 38.76 | 25.36 | 47.179 | *rps4* | 48.92047 | 1.741465779 |
| MT239364 | 39.74 | 51.28 | 42.31 | 25.64 | 46.54 | *rps7* | 49.13024 | 2.590244317 |
| MT239364 | 33.83 | 39.85 | 39.1 | 22.56 | 39.636 | *rps8* | 46.80047 | 7.164474969 |
| MT239364 | 41.25 | 48.2 | 53.96 | 21.58 | 51.883 | *rps11* | 46.05296 | -5.830044933 |
| MT239364 | 41.67 | 50.81 | 46.77 | 27.42 | 44.217 | *rps12* | 50.44926 | 6.232255793 |
| MT239364 | 42.9 | 47.52 | 48.51 | 32.67 | 42.236 | *rps14* | 54.10633 | 11.87033203 |
| MT239364 | 33.33 | 43.01 | 29.03 | 27.96 | 46.014 | *rps15* | 50.84342 | 4.829423716 |
| MT239364 | 34.85 | 51.14 | 31.82 | 21.59 | 48.645 | *rps16* | 46.06059 | -2.584411915 |
| MT239364 | 33.33 | 33.63 | 41.59 | 24.78 | 37.484 | *rps18* | 48.48433 | 11.0003313 |
| MT239364 | 33.95 | 41.67 | 36.11 | 24.07 | 53.343 | *rps19* | 47.94793 | -5.395073823 |
| MT239364 | 35.68 | 43.66 | 36.62 | 26.76 | 51.66 | *rps19* | 49.96344 | -1.696562866 |
| MT239364 | 37.27 | 37.97 | 34.6 | 39.24 | 54.74 | *ycf1* | 57.82525 | 3.085247196 |
| MT239364 | 38.61 | 41.58 | 39.6 | 34.65 | 61 | *Ycf15* | 55.35089 | -5.649110688 |
| MT239364 | 51.79 | 48.76 | 52.07 | 54.55 | 51.452 | *ycf68* | 60.06915 | 8.617146682 |
| *Rhododendron_latoucheae* | 38.33 | 52.36 | 31.14 | 31.48 | 51.791 | *accD* | 53.31743 | 1.526434409 |
| *Rhododendron_latoucheae* | 39.9 | 54.33 | 40.16 | 25.2 | 43.934 | *atpA* | 48.80036 | 4.86635893 |
| *Rhododendron_latoucheae* | 41.82 | 56.31 | 41.68 | 27.45 | 46.307 | *atpB* | 50.47124 | 4.164235751 |
| *Rhododendron_latoucheae* | 38.31 | 48.51 | 38.06 | 28.36 | 45.286 | *atpE* | 51.13328 | 5.847280975 |
| *Rhododendron_latoucheae* | 38.2 | 45.95 | 34.59 | 34.05 | 45.559 | *atpF* | 54.9835 | 9.424496076 |
| *Rhododendron_latoucheae* | 45.53 | 60.98 | 48.78 | 26.83 | 50.468 | *atpH* | 50.01516 | -0.452839759 |
| *Rhododendron_latoucheae* | 38.71 | 48.79 | 37.1 | 30.24 | 46.118 | *atpI* | 52.46747 | 6.349467289 |
| *Rhododendron_latoucheae* | 33.23 | 35.51 | 37.38 | 26.79 | 47.57 | *ccsA* | 49.98561 | 2.415609664 |
| *Rhododendron_latoucheae* | 33.48 | 39.57 | 28.26 | 32.61 | 46.386 | *cemA* | 54.06724 | 7.681241141 |
| *Rhododendron_latoucheae* | 36.18 | 47.56 | 36.59 | 24.39 | 53.977 | *infA* | 48.19 | -5.787004783 |
| *Rhododendron_latoucheae* | 32.68 | 38.66 | 31.56 | 27.81 | 48.172 | *matK* | 50.73425 | 2.562251911 |
| *Rhododendron_latoucheae* | 34.34 | 41.21 | 39.56 | 22.25 | 42.101 | *ndhA* | 46.56416 | 4.463160136 |
| *Rhododendron_latoucheae* | 37.05 | 41.58 | 38.13 | 31.44 | 46.225 | *ndhB* | 53.29044 | 7.065439996 |
| *Rhododendron_latoucheae* | 34.71 | 46.28 | 31.4 | 26.45 | 57.13 | *ndhC* | 49.73385 | -7.396148423 |
| *Rhododendron_latoucheae* | 35.2 | 39.72 | 37.72 | 28.14 | 45.331 | *ndhD* | 50.97409 | 5.643091892 |
| *Rhododendron_latoucheae* | 33.33 | 40.2 | 34.31 | 25.49 | 39.896 | *ndhE* | 49.01793 | 9.121929252 |
| *Rhododendron_latoucheae* | 32.04 | 35.96 | 35.83 | 24.33 | 43.364 | *ndhF* | 48.14464 | 4.780643957 |
| *Rhododendron_latoucheae* | 32.77 | 41.57 | 33.71 | 23.03 | 47.492 | *ndhG* | 47.15837 | -0.333628448 |
| *Rhododendron_latoucheae* | 37.65 | 51.02 | 36.55 | 25.38 | 49.275 | *ndhH* | 48.93547 | -0.339532528 |
| *Rhododendron_latoucheae* | 35.77 | 42.7 | 36.52 | 28.09 | 48.433 | *ndhI* | 50.93783 | 2.504832752 |
| *Rhododendron_latoucheae* | 38.57 | 49.69 | 36.48 | 29.56 | 47.143 | *ndhJ* | 51.99068 | 4.847684669 |
| *Rhododendron_latoucheae* | 36.3 | 42.91 | 40.89 | 25.1 | 48.983 | *ndhK* | 48.72521 | -0.257789981 |
| *Rhododendron_latoucheae* | 38.66 | 47.93 | 38.46 | 29.59 | 51.586 | *pafI* | 52.01187 | 0.42586583 |
| *Rhododendron_latoucheae* | 38.74 | 47.57 | 39.46 | 29.19 | 51.084 | *pafII* | 51.72839 | 0.644390492 |
| *Rhododendron_latoucheae* | 41.67 | 54.55 | 40.91 | 29.55 | 53.121 | *pbf1* | 51.98362 | -1.137378631 |
| *Rhododendron_latoucheae* | 40.08 | 51.71 | 36.45 | 32.09 | 49.512 | *petA* | 53.72534 | 4.213338387 |
| *Rhododendron_latoucheae* | 39.51 | 48.61 | 42.13 | 27.78 | 44.326 | *petB* | 50.71239 | 6.386387453 |
| *Rhododendron_latoucheae* | 37.47 | 50.93 | 39.13 | 22.36 | 44.702 | *petD* | 46.64803 | 1.946033365 |
| *Rhododendron_latoucheae* | 33.33 | 50 | 26.32 | 23.68 | 43.133 | *petG* | 47.6523 | 4.519302359 |
| *Rhododendron_latoucheae* | 32.29 | 31.25 | 43.75 | 21.88 | 55.5 | *petL* | 46.28191 | -9.218087523 |
| *Rhododendron_latoucheae* | 42.22 | 50 | 43.33 | 33.33 | 30.698 | *petN* | 54.53121 | 23.83321187 |
| *Rhododendron_latoucheae* | 42.57 | 52.06 | 43.01 | 32.62 | 50.678 | *psaA* | 54.07376 | 3.395761548 |
| *Rhododendron_latoucheae* | 40.77 | 48.44 | 42.99 | 30.88 | 47.959 | *psaB* | 52.90948 | 4.950475383 |
| *Rhododendron_latoucheae* | 41.87 | 45.12 | 53.66 | 26.83 | 51.756 | *psaC* | 50.01516 | -1.740839759 |
| *Rhododendron_latoucheae* | 32.43 | 40.54 | 29.73 | 27.03 | 41.643 | *psaI* | 50.16269 | 8.519689698 |
| *Rhododendron_latoucheae* | 37.59 | 40.43 | 42.55 | 29.79 | 39.036 | *psaJ* | 52.15273 | 13.11673476 |
| *Rhododendron_latoucheae* | 41.34 | 49.44 | 43.5 | 31.07 | 40.459 | *psbA* | 53.03936 | 12.58035769 |
| *Rhododendron_latoucheae* | 44.07 | 54.62 | 45.97 | 31.63 | 48.964 | *psbB* | 53.4184 | 4.454397798 |
| *Rhododendron_latoucheae* | 44.16 | 54.7 | 46.58 | 31.2 | 45.138 | *psbC* | 53.12786 | 7.989857351 |
| *Rhododendron_latoucheae* | 42.37 | 51.98 | 43.22 | 31.92 | 45.571 | *psbD* | 53.61238 | 8.041382492 |
| *Rhododendron_latoucheae* | 41.67 | 44.05 | 47.62 | 33.33 | 50.06 | *psbE* | 54.53121 | 4.471211875 |
| *Rhododendron_latoucheae* | 39.17 | 47.5 | 45 | 25 | 44 | *psbF* | 48.65 | 4.65 |
| *Rhododendron_latoucheae* | 38.29 | 37.84 | 47.3 | 29.73 | 42.614 | *psbH* | 52.11054 | 9.496536435 |
| *Rhododendron_latoucheae* | 37.84 | 54.05 | 32.43 | 27.03 | 27.272 | *psbI* | 50.16269 | 22.8906897 |
| *Rhododendron_latoucheae* | 41.46 | 46.34 | 48.78 | 29.27 | 35.964 | *psbJ* | 51.78527 | 15.82126637 |
| *Rhododendron_latoucheae* | 35.48 | 38.71 | 33.87 | 33.87 | 51.871 | *psbK* | 54.87156 | 3.000564832 |
| *Rhododendron_latoucheae* | 32.48 | 35.9 | 25.64 | 35.9 | 52.793 | *psbL* | 56.08638 | 3.293383551 |
| *Rhododendron_latoucheae* | 29.52 | 45.71 | 25.71 | 17.14 | 47 | *psbM* | 42.67669 | -4.323313026 |
| *Rhododendron_latoucheae* | 34.26 | 38.89 | 30.56 | 33.33 | 56.893 | *psbT* | 54.53121 | -2.361788125 |
| *Rhododendron_latoucheae* | 35.98 | 41.27 | 41.27 | 25.4 | 46.568 | *psbZ* | 48.95047 | 2.382466527 |
| *Rhododendron_latoucheae* | 43.7 | 58.4 | 43.49 | 29.2 | 47.119 | *rbcL* | 51.7355 | 4.616504828 |
| *Rhododendron_latoucheae* | 42.29 | 48.51 | 48.13 | 30.22 | 51.322 | *rpl2* | 52.45355 | 1.131546387 |
| *Rhododendron_latoucheae* | 37.75 | 52.94 | 35.29 | 25 | 47.001 | *rpl14* | 48.65 | 1.649 |
| *Rhododendron_latoucheae* | 40.2 | 48.53 | 52.21 | 19.85 | 36.233 | *rpl16* | 44.73269 | 8.499686853 |
| *Rhododendron_latoucheae* | 38.3 | 37.72 | 48.25 | 28.95 | 40.816 | *rpl20* | 51.55724 | 10.74123702 |
| *Rhododendron_latoucheae* | 34.41 | 39.35 | 39.35 | 24.52 | 42.675 | *rpl22* | 48.2882 | 5.613195139 |
| *Rhododendron_latoucheae* | 32.99 | 40.62 | 34.38 | 23.96 | 49.323 | *rpl23* | 47.86461 | -1.458391504 |
| *Rhododendron_latoucheae* | 35.78 | 35.29 | 48.53 | 23.53 | 47.675 | *rpl32* | 47.53844 | -0.136556515 |
| *Rhododendron_latoucheae* | 37.44 | 39.73 | 35.62 | 36.99 | 53.171 | *rpl33* | 56.69207 | 3.521069653 |
| *Rhododendron_latoucheae* | 37.72 | 39.47 | 50 | 23.68 | 36.35 | *rpl36* | 47.6523 | 11.30230236 |
| *Rhododendron_latoucheae* | 34.68 | 42.28 | 35.66 | 26.1 | 45.763 | *rpoA* | 49.47366 | 3.71066211 |
| *Rhododendron_latoucheae* | 38.09 | 48.83 | 36.81 | 28.64 | 48.444 | *rpoB* | 51.33503 | 2.891032429 |
| *Rhododendron_latoucheae* | 37.85 | 48.69 | 36.88 | 27.99 | 48.518 | *rpoC1* | 50.86523 | 2.347227613 |
| *Rhododendron_latoucheae* | 36.74 | 45.22 | 36.54 | 28.45 | 49.34 | *rpoC2* | 51.19824 | 1.858235567 |
| *Rhododendron_latoucheae* | 37.29 | 42.37 | 39.83 | 29.66 | 49.979 | *rps2* | 52.06124 | 2.08223729 |
| *Rhododendron_latoucheae* | 35.36 | 46.09 | 34.35 | 25.65 | 49.304 | *rps3* | 49.13773 | -0.166273587 |
| *Rhododendron_latoucheae* | 35.2 | 47.83 | 31.68 | 26.09 | 52.545 | *rpS3* | 49.46621 | -3.078786414 |
| *Rhododendron_latoucheae* | 36.36 | 46.41 | 38.28 | 24.4 | 47.286 | *rps4* | 48.19755 | 0.911552051 |
| *Rhododendron_latoucheae* | 39.32 | 50.64 | 41.67 | 25.64 | 46.097 | *rps7* | 49.13024 | 3.033244317 |
| *Rhododendron_latoucheae* | 33.33 | 39.85 | 38.35 | 21.8 | 41.299 | *rps8* | 46.22086 | 4.921864738 |
| *Rhododendron_latoucheae* | 41.49 | 48.2 | 53.96 | 22.3 | 51.45 | *rps11* | 46.60229 | -4.84771319 |
| *Rhododendron_latoucheae* | 40.05 | 50 | 45.16 | 25 | 42.266 | *rps12* | 48.65 | 6.384 |
| *Rhododendron_latoucheae* | 42.57 | 47.52 | 47.52 | 32.67 | 43.475 | *rps14* | 54.10633 | 10.63133203 |
| *Rhododendron_latoucheae* | 32.26 | 43.01 | 25.81 | 27.96 | 52.83 | *rps15* | 50.84342 | -1.986576284 |
| *Rhododendron_latoucheae* | 30 | 41.43 | 22.86 | 25.71 | 47.691 | *rps15* | 49.1826 | 1.491604079 |
| *Rhododendron_latoucheae* | 34.47 | 51.14 | 31.82 | 20.45 | 47.93 | *rps16* | 45.19036 | -2.739643088 |
| *Rhododendron_latoucheae* | 33.33 | 33.63 | 40.71 | 25.66 | 37.765 | *rps18* | 49.14521 | 11.3802078 |
| *Rhododendron_latoucheae* | 33.64 | 41.67 | 35.19 | 24.07 | 49.242 | *rps19* | 47.94793 | -1.294073823 |
| *Rhododendron_latoucheae* | 36.15 | 42.25 | 39.44 | 26.76 | 56.289 | *rps19* | 49.96344 | -6.325562866 |
| *Rhododendron_latoucheae* | 36.86 | 33.74 | 38.21 | 38.62 | 55.458 | *ycf1* | 57.52967 | 2.071671142 |
| *Rhododendron_latoucheae* | 37.04 | 34.72 | 38.89 | 37.5 | 60.567 | *ycf2* | 56.96324 | -3.603764706 |
| *Rhododendron_latoucheae* | 39.27 | 41.58 | 39.6 | 36.63 | 61 | *Ycf15* | 56.49589 | -4.504114743 |
| *Rhododendron_latoucheae* | 51.79 | 48.76 | 52.07 | 54.55 | 51.452 | *ycf68* | 60.06915 | 8.617146682 |
| *Rhododendron_ovatum* | 37.96 | 51.53 | 30.63 | 31.71 | 51.011 | *accD* | 53.47207 | 2.461071658 |
| *Rhododendron_ovatum* | 39.96 | 54.33 | 40.16 | 25.39 | 44.372 | *atpA* | 48.94297 | 4.57096733 |
| *Rhododendron_ovatum* | 41.95 | 56.51 | 41.68 | 27.66 | 46.32 | *atpB* | 50.62483 | 4.304831035 |
| *Rhododendron_ovatum* | 38.31 | 49.25 | 38.06 | 27.61 | 45.338 | *atpE* | 50.5883 | 5.250303186 |
| *Rhododendron_ovatum* | 38.02 | 45.95 | 34.59 | 33.51 | 45.383 | *atpF* | 54.64541 | 9.262410099 |
| *Rhododendron_ovatum* | 45.12 | 60.98 | 48.78 | 25.61 | 50.468 | *atpH* | 49.10779 | -1.360206193 |
| *Rhododendron_ovatum* | 38.71 | 49.19 | 37.1 | 29.84 | 46.134 | *atpI* | 52.18786 | 6.053858722 |
| *Rhododendron_ovatum* | 33.23 | 35.51 | 37.38 | 26.79 | 47.491 | *ccsA* | 49.98561 | 2.494609664 |
| *Rhododendron_ovatum* | 33.23 | 35.51 | 37.38 | 26.79 | 47.491 | *ccsA* | 49.98561 | 2.494609664 |
| *Rhododendron_ovatum* | 33.48 | 39.57 | 28.26 | 32.61 | 46.464 | *cemA* | 54.06724 | 7.603241141 |
| *Rhododendron_ovatum* | 33.48 | 39.57 | 28.26 | 32.61 | 46.464 | *cemA* | 54.06724 | 7.603241141 |
| *Rhododendron_ovatum* | 36.18 | 46.34 | 37.8 | 24.39 | 51.463 | *infA* | 48.19 | -3.273004783 |
| *Rhododendron_ovatum* | 32.68 | 39.25 | 31.16 | 27.61 | 49.85 | *matK* | 50.5883 | 0.738303186 |
| *Rhododendron_ovatum* | 34.34 | 41.48 | 39.56 | 21.98 | 41.8 | *ndhA* | 46.35821 | 4.558212705 |
| *Rhododendron_ovatum* | 34.34 | 41.48 | 39.56 | 21.98 | 41.8 | *ndhA* | 46.35821 | 4.558212705 |
| *Rhododendron_ovatum* | 36.98 | 41.38 | 38.34 | 31.24 | 45.872 | *ndhB* | 53.15503 | 7.283027132 |
| *Rhododendron_ovatum* | 35.26 | 47.11 | 31.4 | 27.27 | 56.338 | *ndhC* | 50.33922 | -5.998782304 |
| *Rhododendron_ovatum* | 35.13 | 40.12 | 37.72 | 27.54 | 44.463 | *ndhD* | 50.53712 | 6.074119377 |
| *Rhododendron_ovatum* | 35.13 | 40.12 | 37.72 | 27.54 | 44.463 | *ndhD* | 50.53712 | 6.074119377 |
| *Rhododendron_ovatum* | 33.33 | 40.2 | 34.31 | 25.49 | 39.896 | *ndhE* | 49.01793 | 9.121929252 |
| *Rhododendron_ovatum* | 33.33 | 40.2 | 34.31 | 25.49 | 39.896 | *ndhE* | 49.01793 | 9.121929252 |
| *Rhododendron_ovatum* | 31.95 | 35.96 | 35.43 | 24.47 | 43.052 | *ndhF* | 48.25044 | 5.198435995 |
| *Rhododendron_ovatum* | 31.95 | 35.96 | 35.43 | 24.47 | 43.052 | *ndhF* | 48.25044 | 5.198435995 |
| *Rhododendron_ovatum* | 32.77 | 41.57 | 33.71 | 23.03 | 48.185 | *ndhG* | 47.15837 | -1.026628448 |
| *Rhododendron_ovatum* | 32.77 | 41.57 | 33.71 | 23.03 | 48.185 | *ndhG* | 47.15837 | -1.026628448 |
| *Rhododendron_ovatum* | 37.73 | 51.02 | 36.55 | 25.63 | 49.504 | *ndhH* | 49.12276 | -0.381238483 |
| *Rhododendron_ovatum* | 37.73 | 51.02 | 36.55 | 25.63 | 49.504 | *ndhH* | 49.12276 | -0.381238483 |
| *Rhododendron_ovatum* | 35.96 | 42.7 | 36.52 | 28.65 | 49.599 | *ndhI* | 51.34222 | 1.743219685 |
| *Rhododendron_ovatum* | 35.96 | 42.7 | 36.52 | 28.65 | 49.599 | *ndhI* | 51.34222 | 1.743219685 |
| *Rhododendron_ovatum* | 38.57 | 49.69 | 36.48 | 29.56 | 46.506 | *ndhJ* | 51.99068 | 5.484684669 |
| *Rhododendron_ovatum* | 36.96 | 44.35 | 42.17 | 24.35 | 49.325 | *ndhK* | 48.15976 | -1.165237014 |
| *Rhododendron_ovatum* | 38.66 | 47.93 | 38.46 | 29.59 | 51.586 | *pafI* | 52.01187 | 0.42586583 |
| *Rhododendron_ovatum* | 38.92 | 48.11 | 40 | 28.65 | 50.179 | *pafII* | 51.34222 | 1.163219685 |
| *Rhododendron_ovatum* | 38.92 | 48.11 | 40 | 28.65 | 50.179 | *pafII* | 51.34222 | 1.163219685 |
| *Rhododendron_ovatum* | 42.42 | 54.55 | 40.91 | 31.82 | 46.02 | *pbf1* | 53.54567 | 7.525673867 |
| *Rhododendron_ovatum* | 40.29 | 52.34 | 36.45 | 32.09 | 50.219 | *petA* | 53.72534 | 3.506338387 |
| *Rhododendron_ovatum* | 40.29 | 52.34 | 36.45 | 32.09 | 50.219 | *petA* | 53.72534 | 3.506338387 |
| *Rhododendron_ovatum* | 39.51 | 48.61 | 42.13 | 27.78 | 44.245 | *petB* | 50.71239 | 6.467387453 |
| *Rhododendron_ovatum* | 37.68 | 50.93 | 39.13 | 22.98 | 44.953 | *petD* | 47.12032 | 2.167323468 |
| *Rhododendron_ovatum* | 34.21 | 50 | 28.95 | 23.68 | 43.133 | *petG* | 47.6523 | 4.519302359 |
| *Rhododendron_ovatum* | 32.29 | 31.25 | 43.75 | 21.88 | 55.5 | *petL* | 46.28191 | -9.218087523 |
| *Rhododendron_ovatum* | 41.11 | 50 | 43.33 | 30 | 30.698 | *petN* | 52.3 | 21.602 |
| *Rhododendron_ovatum* | 42.61 | 52.06 | 43.14 | 32.62 | 50.212 | *psaA* | 54.07376 | 3.861761548 |
| *Rhododendron_ovatum* | 40.86 | 48.44 | 42.99 | 31.16 | 48.418 | *psaB* | 53.10066 | 4.682658818 |
| *Rhododendron_ovatum* | 41.87 | 45.12 | 53.66 | 26.83 | 51.756 | *psaC* | 50.01516 | -1.740839759 |
| *Rhododendron_ovatum* | 41.87 | 45.12 | 53.66 | 26.83 | 51.756 | *psaC* | 50.01516 | -1.740839759 |
| *Rhododendron_ovatum* | 31.53 | 40.54 | 29.73 | 24.32 | 33.643 | *psaI* | 48.13708 | 14.49408372 |
| *Rhododendron_ovatum* | 31.53 | 40.54 | 29.73 | 24.32 | 33.643 | *psaI* | 48.13708 | 14.49408372 |
| *Rhododendron_ovatum* | 37.59 | 40.43 | 42.55 | 29.79 | 39.036 | *psaJ* | 52.15273 | 13.11673476 |
| *Rhododendron_ovatum* | 41.34 | 49.44 | 43.79 | 30.79 | 40.75 | *psbA* | 52.84773 | 12.09773339 |
| *Rhododendron_ovatum* | 44.01 | 54.62 | 45.97 | 31.43 | 48.083 | *psbB* | 53.28369 | 5.200686767 |
| *Rhododendron_ovatum* | 44.37 | 54.27 | 46.58 | 32.26 | 45.53 | *psbC* | 53.83772 | 8.307720079 |
| *Rhododendron_ovatum* | 42.47 | 51.98 | 43.22 | 32.2 | 45.766 | *psbD* | 53.79812 | 8.032122179 |
| *Rhododendron_ovatum* | 41.27 | 44.05 | 47.62 | 32.14 | 45.12 | *psbE* | 53.75845 | 8.638451903 |
| *Rhododendron_ovatum* | 39.17 | 47.5 | 45 | 25 | 44 | *psbF* | 48.65 | 4.65 |
| *Rhododendron_ovatum* | 38.29 | 37.84 | 47.3 | 29.73 | 42.115 | *psbH* | 52.11054 | 9.995536435 |
| *Rhododendron_ovatum* | 37.84 | 54.05 | 32.43 | 27.03 | 27.272 | *psbI* | 50.16269 | 22.8906897 |
| *Rhododendron_ovatum* | 41.46 | 46.34 | 48.78 | 29.27 | 35.964 | *psbJ* | 51.78527 | 15.82126637 |
| *Rhododendron_ovatum* | 36.02 | 38.71 | 33.87 | 35.48 | 51.871 | *psbK* | 55.84394 | 3.972944876 |
| *Rhododendron_ovatum* | 32.48 | 35.9 | 25.64 | 35.9 | 52.793 | *psbL* | 56.08638 | 3.293383551 |
| *Rhododendron_ovatum* | 30.48 | 45.71 | 25.71 | 20 | 50 | *psbM* | 44.84706 | -5.152941176 |
| *Rhododendron_ovatum* | 34.26 | 38.89 | 30.56 | 33.33 | 56.893 | *psbT* | 54.53121 | -2.361788125 |
| *Rhododendron_ovatum* | 35.98 | 41.27 | 41.27 | 25.4 | 47.965 | *psbZ* | 48.95047 | 0.985466527 |
| *Rhododendron_ovatum* | 43.63 | 58.4 | 43.28 | 29.2 | 46.975 | *rbcL* | 51.7355 | 4.760504828 |
| *Rhododendron_ovatum* | 42.04 | 48.51 | 48.13 | 29.48 | 49.282 | *rpl2* | 51.93414 | 2.652137689 |
| *Rhododendron_ovatum* | 39.51 | 54.81 | 37.04 | 26.67 | 50.135 | *rpl14* | 49.89687 | -0.238129289 |
| *Rhododendron_ovatum* | 40.2 | 48.53 | 52.21 | 19.85 | 36.381 | *rpl16* | 44.73269 | 8.351686853 |
| *Rhododendron_ovatum* | 38.3 | 37.72 | 48.25 | 28.95 | 43.697 | *rpl20* | 51.55724 | 7.860237022 |
| *Rhododendron_ovatum* | 34.19 | 38.71 | 39.35 | 24.52 | 44.183 | *rpl22* | 48.2882 | 4.105195139 |
| *Rhododendron_ovatum* | 33.33 | 40.62 | 34.38 | 25 | 50.734 | *rpl23* | 48.65 | -2.084 |
| *Rhododendron_ovatum* | 36.27 | 35.29 | 48.53 | 25 | 47.179 | *rpl32* | 48.65 | 1.471 |
| *Rhododendron_ovatum* | 36.27 | 35.29 | 48.53 | 25 | 47.179 | *rpl32* | 48.65 | 1.471 |
| *Rhododendron_ovatum* | 36.99 | 38.36 | 35.62 | 36.99 | 54.783 | *rpl33* | 56.69207 | 1.909069653 |
| *Rhododendron_ovatum* | 38.6 | 42.11 | 50 | 23.68 | 32.946 | *rpl36* | 47.6523 | 14.70630236 |
| *Rhododendron_ovatum* | 35.03 | 43.64 | 36 | 25.45 | 45.234 | *rpoA* | 48.98795 | 3.75395253 |
| *Rhododendron_ovatum* | 37.96 | 48.73 | 36.83 | 28.3 | 48.354 | *rpoB* | 51.08992 | 2.735923178 |
| *Rhododendron_ovatum* | 37.95 | 49.42 | 36.88 | 27.55 | 48.47 | *rpoC1* | 50.54443 | 2.07443453 |
| *Rhododendron_ovatum* | 36.79 | 45.22 | 36.47 | 28.67 | 49.564 | *rpoC2* | 51.35659 | 1.79259038 |
| *Rhododendron_ovatum* | 37.57 | 41.95 | 41.1 | 29.66 | 50.048 | *rps2* | 52.06124 | 2.01323729 |
| *Rhododendron_ovatum* | 35.01 | 45.19 | 33.05 | 26.78 | 51.357 | *rps3* | 49.97822 | -1.378780268 |
| *Rhododendron_ovatum* | 34.55 | 46.67 | 31.52 | 25.45 | 48.913 | *rpS3* | 48.98795 | 0.07495253 |
| *Rhododendron_ovatum* | 34.55 | 46.67 | 31.52 | 25.45 | 48.913 | *rpS3* | 48.98795 | 0.07495253 |
| *Rhododendron_ovatum* | 36.84 | 46.41 | 38.28 | 25.84 | 46.768 | *rps4* | 49.27975 | 2.511749898 |
| *Rhododendron_ovatum* | 39.74 | 51.28 | 42.31 | 25.64 | 46.54 | *rps7* | 49.13024 | 2.590244317 |
| *Rhododendron_ovatum* | 34.34 | 39.85 | 39.85 | 23.31 | 45.47 | *rps8* | 47.37131 | 1.901308681 |
| *Rhododendron_ovatum* | 41.49 | 48.92 | 53.96 | 21.58 | 50.58 | *rps11* | 46.05296 | -4.527044933 |
| *Rhododendron_ovatum* | 42.2 | 51.61 | 46.77 | 28.23 | 47.483 | *rps12* | 51.03928 | 3.556284396 |
| *Rhododendron_ovatum* | 41.91 | 45.54 | 46.53 | 33.66 | 44.995 | *rps14* | 54.74001 | 9.745008231 |
| *Rhododendron_ovatum* | 33.33 | 44.09 | 27.96 | 27.96 | 49.569 | *rps15* | 50.84342 | 1.274423716 |
| *Rhododendron_ovatum* | 33.33 | 44.09 | 27.96 | 27.96 | 49.569 | *rps15* | 50.84342 | 1.274423716 |
| *Rhododendron_ovatum* | 37.23 | 54.55 | 36.36 | 20.78 | 48.656 | *rps16* | 45.44223 | -3.213768361 |
| *Rhododendron_ovatum* | 37.23 | 54.55 | 36.36 | 20.78 | 48.656 | *rps16* | 45.44223 | -3.213768361 |
| *Rhododendron_ovatum* | 33.33 | 33.63 | 41.59 | 24.78 | 37.484 | *rps18* | 48.48433 | 11.0003313 |
| *Rhododendron_ovatum* | 26.92 | 30.77 | 36.54 | 13.46 | 27.549 | *rps18* | 39.94256 | 12.39355623 |
| *Rhododendron_ovatum* | 33.95 | 41.67 | 36.11 | 24.07 | 47.927 | *rps19* | 47.94793 | 0.020926177 |
| *Rhododendron_ovatum* | 35.68 | 42.25 | 38.03 | 26.76 | 50.233 | *rps19* | 49.96344 | -0.269562866 |
| *Rhododendron_ovatum* | 36.56 | 37.9 | 32.66 | 39.11 | 56.373 | *ycf1* | 57.76437 | 1.391367184 |
| *Rhododendron_ovatum* | 36.56 | 37.9 | 32.66 | 39.11 | 56.373 | *ycf1* | 57.76437 | 1.391367184 |
| *Rhododendron_ovatum* | 40.48 | 50 | 28.57 | 42.86 | 27.891 | *ycf2* | 59.26951 | 31.37850927 |
| *Rhododendron_ovatum* | 51.49 | 54.47 | 47.15 | 52.85 | 61 | *ycf68* | 60.34067 | -0.659331735 |
| *Rhododendron_ovatum* | 51.49 | 54.47 | 47.15 | 52.85 | 61 | *ycf68* | 60.34067 | -0.659331735 |
| *Rhododendron_ripense* | 37.01 | 51.1 | 29.71 | 30.22 | 49.968 | *accD* | 52.45355 | 2.485546387 |
| *Rhododendron_ripense* | 40.03 | 54.33 | 40.16 | 25.59 | 44.396 | *atpA* | 49.09282 | 4.696823297 |
| *Rhododendron_ripense* | 41.88 | 56.31 | 41.68 | 27.66 | 46.197 | *atpB* | 50.62483 | 4.427831035 |
| *Rhododendron_ripense* | 38.31 | 48.51 | 38.06 | 28.36 | 45.286 | *atpE* | 51.13328 | 5.847280975 |
| *Rhododendron_ripense* | 38.02 | 45.95 | 34.59 | 33.51 | 44.695 | *atpF* | 54.64541 | 9.950410099 |
| *Rhododendron_ripense* | 45.53 | 60.98 | 48.78 | 26.83 | 50.468 | *atpH* | 50.01516 | -0.452839759 |
| *Rhododendron_ripense* | 38.84 | 49.19 | 37.1 | 30.24 | 46.118 | *atpI* | 52.46747 | 6.349467289 |
| *Rhododendron_ripense* | 33.33 | 35.2 | 37.38 | 27.41 | 47.885 | *ccsA* | 50.44193 | 2.556927074 |
| *Rhododendron_ripense* | 33.62 | 39.57 | 28.26 | 33.04 | 46.487 | *cemA* | 54.34569 | 7.858694936 |
| *Rhododendron_ripense* | 35.77 | 46.34 | 37.8 | 23.17 | 50.637 | *infA* | 47.26487 | -3.372130897 |
| *Rhododendron_ripense* | 32.61 | 39.05 | 31.36 | 27.42 | 47.32 | *matK* | 50.44926 | 3.129255793 |
| *Rhododendron_ripense* | 34.34 | 41.21 | 39.56 | 22.25 | 41.855 | *ndhA* | 46.56416 | 4.709160136 |
| *Rhododendron_ripense* | 36.98 | 41.38 | 38.13 | 31.44 | 45.878 | *ndhB* | 53.29044 | 7.412439996 |
| *Rhododendron_ripense* | 35.54 | 47.11 | 31.4 | 28.1 | 57.129 | *ndhC* | 50.94509 | -6.183913089 |
| *Rhododendron_ripense* | 35.33 | 40.12 | 37.92 | 27.94 | 45.185 | *ndhD* | 50.82888 | 5.643882101 |
| *Rhododendron_ripense* | 33.01 | 40.2 | 34.31 | 24.51 | 40.342 | *ndhE* | 48.28064 | 7.93864433 |
| *Rhododendron_ripense* | 31.82 | 35.83 | 35.29 | 24.33 | 43.044 | *ndhF* | 48.14464 | 5.100643957 |
| *Rhododendron_ripense* | 32.77 | 41.57 | 33.71 | 23.03 | 47.73 | *ndhG* | 47.15837 | -0.571628448 |
| *Rhododendron_ripense* | 37.65 | 51.02 | 36.55 | 25.38 | 49.275 | *ndhH* | 48.93547 | -0.339532528 |
| *Rhododendron_ripense* | 35.98 | 42.61 | 36.93 | 28.41 | 48.052 | *ndhI* | 51.16938 | 3.117379095 |
| *Rhododendron_ripense* | 38.78 | 49.69 | 36.48 | 30.19 | 47.744 | *ndhJ* | 52.43265 | 4.688653105 |
| *Rhododendron_ripense* | 36.81 | 43.91 | 42.17 | 24.35 | 49.309 | *ndhK* | 48.15976 | -1.149237014 |
| *Rhododendron_ripense* | 38.66 | 47.93 | 38.46 | 29.59 | 51.586 | *pafI* | 52.01187 | 0.42586583 |
| *Rhododendron_ripense* | 38.38 | 48.11 | 39.46 | 27.57 | 48.808 | *pafII* | 50.55906 | 1.751061663 |
| *Rhododendron_ripense* | 42.42 | 54.55 | 40.91 | 31.82 | 46.02 | *pbf1* | 53.54567 | 7.525673867 |
| *Rhododendron_ripense* | 39.98 | 51.71 | 36.45 | 31.78 | 49.443 | *petA* | 53.51894 | 4.075936284 |
| *Rhododendron_ripense* | 39.51 | 48.15 | 42.13 | 28.24 | 44.175 | *petB* | 51.04652 | 6.87152209 |
| *Rhododendron_ripense* | 37.47 | 50.93 | 39.13 | 22.36 | 44.702 | *petD* | 46.64803 | 1.946033365 |
| *Rhododendron_ripense* | 34.21 | 50 | 28.95 | 23.68 | 43.133 | *petG* | 47.6523 | 4.519302359 |
| *Rhododendron_ripense* | 31.25 | 28.12 | 43.75 | 21.88 | 55.5 | *petL* | 46.28191 | -9.218087523 |
| *Rhododendron_ripense* | 41.11 | 50 | 43.33 | 30 | 30.698 | *petN* | 52.3 | 21.602 |
| *Rhododendron_ripense* | 42.52 | 52.06 | 43.01 | 32.49 | 50.515 | *psaA* | 53.98883 | 3.47383317 |
| *Rhododendron_ripense* | 40.91 | 48.44 | 42.99 | 31.29 | 48.467 | *psaB* | 53.18895 | 4.721948718 |
| *Rhododendron_ripense* | 41.87 | 45.12 | 53.66 | 26.83 | 51.756 | *psaC* | 50.01516 | -1.740839759 |
| *Rhododendron_ripense* | 32.43 | 40.54 | 29.73 | 27.03 | 41.643 | *psaI* | 50.16269 | 8.519689698 |
| *Rhododendron_ripense* | 36.88 | 40.43 | 40.43 | 29.79 | 37.61 | *psaJ* | 52.15273 | 14.54273476 |
| *Rhododendron_ripense* | 41.24 | 49.44 | 43.79 | 30.51 | 40.206 | *psbA* | 52.65477 | 12.44876803 |
| *Rhododendron_ripense* | 44.14 | 54.81 | 45.97 | 31.63 | 48.625 | *psbB* | 53.4184 | 4.793397798 |
| *Rhododendron_ripense* | 44.37 | 54.7 | 46.58 | 31.84 | 45.957 | *psbC* | 53.55903 | 7.602031092 |
| *Rhododendron_ripense* | 42.47 | 51.98 | 43.22 | 32.2 | 45.766 | *psbD* | 53.79812 | 8.032122179 |
| *Rhododendron_ripense* | 41.67 | 44.05 | 47.62 | 33.33 | 45.12 | *psbE* | 54.53121 | 9.411211875 |
| *Rhododendron_ripense* | 39.17 | 47.5 | 45 | 25 | 44 | *psbF* | 48.65 | 4.65 |
| *Rhododendron_ripense* | 37.84 | 37.84 | 47.3 | 28.38 | 41.647 | *psbH* | 51.14772 | 9.50072388 |
| *Rhododendron_ripense* | 36.94 | 54.05 | 32.43 | 24.32 | 25.494 | *psbI* | 48.13708 | 22.64308372 |
| *Rhododendron_ripense* | 41.46 | 46.34 | 48.78 | 29.27 | 35.964 | *psbJ* | 51.78527 | 15.82126637 |
| *Rhododendron_ripense* | 35.48 | 38.71 | 33.87 | 33.87 | 50.008 | *psbK* | 54.87156 | 4.863564832 |
| *Rhododendron_ripense* | 32.48 | 35.9 | 25.64 | 35.9 | 52.793 | *psbL* | 56.08638 | 3.293383551 |
| *Rhododendron_ripense* | 29.52 | 45.71 | 25.71 | 17.14 | 47 | *psbM* | 42.67669 | -4.323313026 |
| *Rhododendron_ripense* | 34.26 | 38.89 | 30.56 | 33.33 | 56.893 | *psbT* | 54.53121 | -2.361788125 |
| *Rhododendron_ripense* | 36.51 | 41.27 | 41.27 | 26.98 | 46.925 | *psbZ* | 50.12584 | 3.200842687 |
| *Rhododendron_ripense* | 43.49 | 58.4 | 43.28 | 28.78 | 47.347 | *rbcL* | 51.43554 | 4.088537557 |
| *Rhododendron_ripense* | 42.29 | 47.76 | 48.88 | 30.22 | 50.449 | *rpl2* | 52.45355 | 2.004546387 |
| *Rhododendron_ripense* | 39.6 | 54.89 | 37.59 | 26.32 | 48.262 | *rpl14* | 49.63733 | 1.375327271 |
| *Rhododendron_ripense* | 37.43 | 36.84 | 47.37 | 28.07 | 43 | *rpl20* | 50.92332 | 7.923320953 |
| *Rhododendron_ripense* | 33.55 | 38.71 | 38.06 | 23.87 | 41.498 | *rpl22* | 47.7964 | 6.298401515 |
| *Rhododendron_ripense* | 33.33 | 40.62 | 34.38 | 25 | 50.734 | *rpl23* | 48.65 | -2.084 |
| *Rhododendron_ripense* | 35.78 | 35.29 | 48.53 | 23.53 | 47.675 | *rpl32* | 47.53844 | -0.136556515 |
| *Rhododendron_ripense* | 36.53 | 36.99 | 35.62 | 36.99 | 52.088 | *rpl33* | 56.69207 | 4.604069653 |
| *Rhododendron_ripense* | 36.84 | 39.47 | 50 | 21.05 | 31.625 | *rpl36* | 45.64835 | 14.02335129 |
| *Rhododendron_ripense* | 34.98 | 44.11 | 36.12 | 24.71 | 42.861 | *rpoA* | 48.43156 | 5.570561184 |
| *Rhododendron_ripense* | 37.84 | 48.17 | 36.81 | 28.54 | 48 | *rpoB* | 51.26309 | 3.263090358 |
| *Rhododendron_ripense* | 37.8 | 49.13 | 36.88 | 27.41 | 48.247 | *rpoC1* | 50.44193 | 2.194927074 |
| *Rhododendron_ripense* | 36.83 | 45.15 | 36.69 | 28.67 | 49.539 | *rpoC2* | 51.35659 | 1.81759038 |
| *Rhododendron_ripense* | 37.41 | 41.77 | 40.51 | 29.96 | 50.514 | *rps2* | 52.272 | 1.758001449 |
| *Rhododendron_ripense* | 34.89 | 44.92 | 34.75 | 25 | 49.019 | *rps3* | 48.65 | -0.369 |
| *Rhododendron_ripense* | 34.14 | 46.06 | 30.91 | 25.45 | 48.905 | *rpS3* | 48.98795 | 0.08295253 |
| *Rhododendron_ripense* | 36.84 | 46.89 | 38.76 | 24.88 | 47.043 | *rps4* | 48.55967 | 1.51666973 |
| *Rhododendron_ripense* | 39.74 | 51.28 | 42.31 | 25.64 | 46.54 | *rps7* | 49.13024 | 2.590244317 |
| *Rhododendron_ripense* | 34.09 | 39.85 | 39.1 | 23.31 | 44.244 | *rps8* | 47.37131 | 3.127308681 |
| *Rhododendron_ripense* | 41.25 | 48.2 | 53.96 | 21.58 | 51.754 | *rps11* | 46.05296 | -5.701044933 |
| *Rhododendron_ripense* | 41.67 | 50.81 | 46.77 | 27.42 | 44.217 | *rps12* | 50.44926 | 6.232255793 |
| *Rhododendron_ripense* | 43.23 | 47.52 | 48.51 | 33.66 | 42.883 | *rps14* | 54.74001 | 11.85700823 |
| *Rhododendron_ripense* | 33.33 | 43.01 | 29.03 | 27.96 | 46.014 | *rps15* | 50.84342 | 4.829423716 |
| *Rhododendron_ripense* | 34.85 | 51.14 | 31.82 | 21.59 | 48.645 | *rps16* | 46.06059 | -2.584411915 |
| *Rhododendron_ripense* | 32.74 | 32.74 | 41.59 | 23.89 | 37.451 | *rps18* | 47.81156 | 10.36056151 |
| *Rhododendron_ripense* | 33.95 | 41.67 | 35.19 | 25 | 50.818 | *rps19* | 48.65 | -2.168 |
| *Rhododendron_ripense* | 35.68 | 43.66 | 36.62 | 26.76 | 52.033 | *rps19* | 49.96344 | -2.069562866 |
| *Rhododendron_ripense* | 36.55 | 37.75 | 32.93 | 38.96 | 55.311 | *ycf1* | 57.69339 | 2.382394039 |
| *Rhododendron_ripense* | 36.22 | 35.43 | 36.22 | 37.01 | 61 | *ycf2* | 56.70285 | -4.297145754 |
| *Rhododendron_ripense* | 38.61 | 41.58 | 39.6 | 34.65 | 61 | *Ycf15* | 55.35089 | -5.649110688 |
| *Rhododendron_ripense* | 51.2 | 50.4 | 48 | 55.2 | 61 | *ycf68* | 59.93138 | -1.068615424 |

**Supplementary Table 15.** Plot of ENC of genes in 15 *Rhododendron* species chloroplast genomes

| Subgenus | Section | Species | Genebank number | ENC plot analysis | | | | |
| --- | --- | --- | --- | --- | --- | --- | --- | --- |
|  |  |  |  | ENC values of gene on or above standard curve line | |  | ENC values of gene below standard curve line | |
|  |  |  |  | Gene number | Gene list |  | Gene number | Gene list |
| Tsutsusi (G.Don) Pojarkova | Tsutsusi | *R. × pulchrum* | MN182619.2 | 18 | *atpH, infA, ndhC, ndhG, ndhH, ndhK, petL, psaC, psbM, psbT, rpl23, rpl32, rps11, rps16, rps19, rps3, Ycf15, ycf68* |  | 62 | *accD, atpA, atpB, atpE, atpI, ccsA, cemA, matK, ndhA, ndhB, ndhD, ndhE, ndhF, ndhI, ndhJ, pafI, pafII, pbf1, petA, petB, petD, petG, petN, psaA, psaB, psaI, psaJ, psbA, psbB, psbC, psbD, psbE, psbF, psbH, psbI, psbJ, psbK, psbL, psbZ, rbcL, rpl14, rpl16, rpl2, rpl20, rpl22, rpl33, rpl36, rpoA, rpoB, rpoC1, rpoC2, rps12, rps14, rps15, rps18, rps2, rpS3, rps4, rps7, rps8, ycf1* |
|  |  | *R. ripense* | DRR298903-DRR298907 | 19 | *atpH, infA, ndhC, ndhG, ndhH, ndhK, petL, psaC, psbM, psbT, rpl23, rpl32, rps11, rps16, rps19, rps3, ycf2, ycf15, ycf68* |  | 61 | *accD, atpA, atpB, atpE, atpF, atpI, ccsA, cemA, matK, ndhA, ndhB, ndhD, ndhE, ndhF, ndhI, ndhJ, pafI, pafII, pbf1, petA, petB, petD, petG, petN, psaA, psaB, psaI, psaJ, sbA, psbB, psbC, psbD, psbE, psbF, psbH, psbI, psbJ, psbK, psbL, psbZ, rbcL, rpl14, rpl2, rpl20, rpl22, rpl33, rpl36, rpoA, rpoB, rpoC1, rpoC2, rps12, rps14, rps15, rps18, rps2, rps3 rps4, rps7, rps8, ycf1* |
|  |  | *R. simsii* | MT239364 | 17 | *atpH, infA, ndhC, ndhG, ndhH, ndhK, petL, psaC, psbM, psbT, rpl23, rpl32, rps11, rps16, rps19, rps3, ycf15* |  | 63 | *accD, atpA, atpB, atpE, atpI, ccsA, cemA, matK, ndhA, ndhB, ndhD, ndhE, ndhF, ndhI, ndhJ, pafI, pafII, pbf1, petA, petB, petD, petG, petN, psaA, psaB, psaI, psaJ, psbA, psbB, psbC, psbD, psbE, psbF, psbH, psbI, psbJ, psbK, psbL, psbZ, rbcL, rpl14, rpl16, rpl2, rpl20, rpl22, rpl33, rpl36, rpoA, rpoB, rpoC1, rpoC2, rps12, rps14, rps15, rps18, rps2, rpS3, rps4, rps7, rps8, ycf1, ycf68* |
| Hymenanthes (BL) K. Koch | Ponticum G. Don | *R. delavayi* | MN711645 | 17 | *atpH, infA, ndhC, ndhG, ndhH, ndhK, petL, psaC, psbM, psbT, rpl23, rpl32, rps11, rps15, rps16, rps3, ycf3* |  | 56 | *accD, atpA, atpB, atpE, atpI, ccsA, cemA, matK, ndhA, ndhB, ndhD, ndhE, ndhF, ndhI, ndhJ, petA, petB, petD, petG, petN, psaA, psaB, psaI, psbA, psbB, psbC, psbD, psbE, psbF, psbH, psbI, psbJ, psbK, psbL, psbN, psbZ, rbcL, rpl14, rpl2, rpl20, rpl22, rpl33, rpl36, rpoA, rpoB, rpoC1, rpoC2, rps12, rps14, rps18, rps2, rps4, rps7, rps8, ycf4* |
|  |  | *R. henanense* subsp. Lingbaoense | MT239363 | 15 | *atpH, ndhC, ndhG, ndhH, ndhK, petL, psaC, psbM, rpl23, rpl32, rps11, rps15, rps16, rps19, ycf3* |  | 56 | *atpA, atpB, atpE, atpF, atpI, ccsA, cemA, matK, ndhA, ndhB, ndhD, ndhE, ndhF, ndhI, ndhJ, petA, petB, petD, petG, petN, psaA, psaB, psaI, psaJ, psbA, psbB, psbC, psbD, psbE, psbF, psbJ, psbK, psbL, psbN, psbZ, rbcL, rpl14, rpl16, rpl2, rpl20, rpl22, rpl33, rpl36, rpoA, rpoB, rpoC1, rpoC2, rps12, rps14, rps18, rps2, rps3, rps4, rps7, rps8, ycf4* |
|  |  | *Rhododendron delavayi* var. delavayi | NC_047438 | 13 | *atpH, ndhC, ndhH, petL, psaC, psbM, psbT, rpl23, rpl32, rps11, rps15, rps19, ycf3* |  | 55 | *atpA, atpB, atpE, atpI, cemA, lhbA, matK, ndhA, ndhB, ndhD, ndhE, ndhF, ndhI, ndhJ, petA, petB, petD, petG, petN, psaA, psaB, psaI, psaJ, psbA, psbB, psbC, psbD, psbE, psbF, psbJ, psbH, psbI, psbK, psbL, psbN, rbcL, rpl14, rpl16, rpl2, rpl22, rpl33, rpl36, rpoA, rpoB, rpoC1, rpoC2, rps12, rps14, rps18, rps2, rps3, rps4, rps7, rps8, ycf4* |
|  |  | *R. platypodum* | MT985162 | 17 | *atpH, infA, ndhC, ndhH, ndhK, petL, psaC, psbM, psbT, rpl23, rpl32, rpl33, rps11, rps15, rps16, rps3, ycf19* |  | 59 | *accD, atpA, atpB, atpE, atpI, ccsA, cemA, matK, ndhA, ndhB, ndhD, ndhE, ndhF, ndhI, ndhJ, petA, petB, petD, petG, petN, psaA, psaB, psaI, psaJ, psbA, psbB, psbC, psbD, psbE, psbF, psbH, psbI, psbJ, psbK, psbL, psbN, psbZ, rbcL, rpl16, rpl2, rpl14, rpl20, rpl22, rpl36, rpoA, rpoB, rpoC1, rpoC2, rps12, rps14, rps18, rps2, rpS3, rps4, rps7, rps8, ycf1, ycf4* |
|  |  | *R. griersonianum* | NC_050162 | 19 | *atpH, infA, ndhC, ndhG, ndhH, ndhK, petL, psaC, psbM, psbT, rpl23, rpl32, rps11, rps15, rps16, rps19, ycf1, ycf3, ycf15* |  | 60 | *accD, atpA, atpB, atpE, atpI, ccsA, cemA, matK, ndhA, ndhB, ndhD, ndhE, ndhF, ndhI, ndhJ, petA, petB, petD, petG, petN, psaA, psaB, psaI, psaJ, psbA, psbB, psbC, psbD, psbE, psbF, psbH, psbI, psbJ, psbK, psbL, psbN, psbZ, rbcL, rpl16, rpl2, rpl14, rpl20, rpl22, rpl33, rpl36, rpoA, rpoB, rpoC1, rpoC2, rps12, rps14, rps18, rps2, rpS3, rps4, rps7, rps8, ycf4, ycf68* |
| Rhododendron | Rhododendron | *R. micranthum* | MT239365 | 17 | *atpH, ndhG, ndhH, ndhK, petL, psaC, psbM, psbT, rpl14, rpl23, rpl32, rps11, rps15, rps16, rps19, rps3, ycf3* |  | 55 | *atpA, atpB, atpE, atpF, atpI, ccsA, cemA, matK, ndhA, ndhB, ndhD, ndhE, ndhI, ndhJ, petA, petB, petD, petG, petN, psaA, psaB, psaI, psaJ, psbA, psbB, psbC, psbD, psbE, psbF, psbH, psbI, psbJ, psbK, psbL, psbN, psbZ, rbcL, rpl16, rpl2, rpl20, rpl22, rpl33, rpl36, rpoA, rpoB, rpoC1, rpoC2, rps12, rps14, rps18, rps2, rps4, rps7, rps8, ycf4* |
|  |  | *R. concinnum* | MT239366 | 17 | *atpH, ndhG, ndhH, ndhK, petL, psaC, psbM, psbT, rpl14, rpl23, rpl32, rps11, rps15, rps16, rps19, rps7, ycf3* |  | 55 | *atpA, atpB, atpE, atpF, atpI, ccsA, cemA, matK, ndhA, ndhB, ndhD, ndhE, ndhI, ndhJ, petA, petB, petD, petG, petN, psaA, psaB, psaI, psaJ, psbA, psbB,psbC, psbD, psbE, psbF, psbH, psbI, psbJ, psbK, psbL, psbN, psbZ, rbcL, rpl16, rpl2, rpl20, rpl22, rpl33, rpl36, rpoA, rpoB, rpoC1, rpoC2, rps12, rps14, rps18, rps2, rps3, rps4, rps8, ycf4* |
|  | [Vireya](https://www.cfh.ac.cn/Taxon/ShowTaxonSystem.aspx?sysid=1137) | *R. datiandingense* | NC_057644 | 17 | *atpH, infA, ndhC, ndhG, ndhH, ndhK, petL, psaC, psbM, psbT, rpl14, rpl23, rpl32, rps11, rps15, rps16, rps19* |  | 59 | *accD, atpA, atpB, atpE, atpI, ccsA, cemA, matK, ndhA, ndhB, ndhD, ndhE, ndhF, ndhI, ndhJ, petA, petB, petD, petG, petN, psaA, psaB, psaI, psaJ, psbA, psbB, psbC, psbD, psbE, psbH, psbI, psbJ, psbK, psbL, psbN, psbZ, rbcL, rpl16, rpl2, rpl20, rpl22, rpl33, rpl36, rpoA, rpoB, rpoC1, rpoC2, rps12, rps14, rps18, rps2, rpS3, rps4, rps7, rps8, ycf3, ycf4, ycf15* |
|  |  | *R. kawakamii* | NC_058233 | 17 | *atpH, infA, ndhC, ndhG, ndhH, ndhK, petL, psaC, psbM, psbT, rpl14, rpl23, rpl32, rps11, rps15, rps16, rps19* |  | 59 | *accD, atpA, atpB, atpE, atpI, ccsA, cemA, matK, ndhA, ndhB, ndhD, ndhE, ndhF, ndhI, ndhJ, petA, petB, petD, petG, petN, psaA, psaB, psaI, psaJ, psbA, psbB, psbC, psbD, psbF, psbH, psbI, psbJ, psbK, psbL, psbN, psbZ, rbcL, rpl16, rpl2, rpl20, rpl22, rpl33, rpl36, rpoA, rpoB, rpoC1, rpoC2, rps12, rps14, rps18, rps2, rpS3, rps4, rps7, rps8, ycf3, ycf4, ycf15* |
| Azaleastrum | Azaleastrum | *R. ovatum* | SRR12917131-SRR12917132 | 18 | *atpH, infA, ndhC, ndhG, ndhG, ndhH, ndhK, petL, psaC, psbM, psbT, rpl14, rpl23, rps11, rps16, rps19, rps3, ycf68* |  | 64 | *accD, atpA, atpB, atpE, atpF, atpI, ccsA, cemA, matK, ndhA, ndhB, ndhD, ndhE, ndhF, ndhI, ndhJ, pafI, pafII, pbf1, petA, petB, petD, petG, petN, psaA, psaB, psaI, psaJ, psbA, psbB, psbC, psbD, psbE, psbF, psbH, psbI, psbJ, psbK, psbL, psbZ, rbcL, rpl16, rpl2, rpl20, rpl22, rpl32, rpl33, rpl36, rpoA, rpoB, rpoC1, rpoC2, rps12, rps14, rps15, rps18, rps19, rps2, rps3, rps4, rps7, rps8, ycf1, ycf2* |
|  | Choniastrum | *R. latoucheae* | SRR13425299 | 20 | *atpH, infA, ndhC, ndhG, ndhH, ndhK, pbf1, petL,psaC, psbM, psbT, rpl23, rpl32, rps11, rps15, rps16, rps19, rps3, ycf15, ycf2* |  | 61 | *accD, atpA, atpB, atpE, atpF, atpI, ccsA, cemA, matK, ndhA, ndhB, ndhD, ndhE, ndhF, ndhI, ndhJ, pafI, pafII, petA, petB, petD, petG, petN, psaA, psaB, psaI, psaJ, psbA, psbB, psbC, psbD, psbE, psbF, psbH, psbI, psbJ, psbK, psbL, psbZ, rbcL, rpl14, rpl16, rpl2, rpl20, rpl22, rpl33, rpl36, rpoA, rpoB, rpoC1, rpoC2, rps12, rps14, rps15, rps18, rps2, rps4, rps7, rps8, ycf1, ycf68* |
| Pentanthera | Pentanthera | *R. molle* | MZ073672 | 16 | *atpH, infA, ndhC, ndhH, ndhK, petL, psaC, psbM, psbT, psbZ, rpl14, rpl23, rpl32, rps11, rps15, rps16* |  | 57 | *accD, atpA, atpB, atpE, atpI, ccsA, cemA, matK, ndhA, ndhB, ndhD, ndhE, ndhI, ndhJ, petA, petB, petD, petG, petN, psaA, psaB, psaI, psaJ, psbA, psbB, psbC, psbD, psbE, psbF, psbH, psbI, psbJ, psbK, psbL, psbN, rbcL, rpl16, rpl2, rpl20, rpl22, rpl33, rpl36, rpoA, rpoB, rpoC1, rpoC2, rps12, rps14, rps18, rps2, rpS3, rps4, rps7, rps8, ycf3, ycf4* |

**Supplementary Table 16 Primers used for PCR in our research.**

| **Primer** | **MN182619.2  （plastid genomes fasta using third-generation combined with second-generation sequencing methods）** | | |
| --- | --- | --- | --- |
|  | **Forward (5'to3')** | **Reverse (5'to3')** | **Length of products （bp）** |
| P1 | CCTCTTCGCAAATTCGGCTTAT | CGCACCATATTTAACCCTCCCAT | 355 |
| P2 | TCCTAAAAGTTTGAAGTGGAGGAA | CCCACAGTCATCCCACCTAG | 444 |
| P3 | GCGGATTTGGTCAGGGAGAT | GGTAAACGACCCAAAGCAATTTG | 300 |
| P4 | GTTGTTCCGATTAGTGCGCG | CATGCCTGTTCGAAATGCCA | 581 |
| P5 | ATGGGGGAGATCTAGGGTGG | TGGGATCCGGACTCTTTTGG | 201 |
| P6 | GTTGTTCCGATTAGTGCGCG | TCGGAGACCCAGGAAAACAG | 348 |
